# Characterization and analysis of *CCR* and *CAD* gene families at the whole-genome level for lignin synthesis of stone cells in pear (*Pyrus bretschneideri*) fruit

**DOI:** 10.1242/bio.026997

**Published:** 2017-11-15

**Authors:** Xi Cheng, Manli Li, Dahui Li, Jinyun Zhang, Qing Jin, Lingling Sheng, Yongping Cai, Yi Lin

**Affiliations:** 1 School of Life Science, Anhui Agricultural University, No. 130, Changjiang West Road, Hefei 230036, China; 2 Horticultural Institute, Anhui Academy of Agricultural Sciences, Hefei, Anhui 230031, China

**Keywords:** *Pyrus bretschneideri*, Stone cells, Lignin synthesis, *CCR* and *CAD* gene families, Expression analysis, Subcellular localization

## Abstract

The content of stone cells has significant effects on the flavour and quality of pear fruit. Previous research suggested that lignin deposition is closely related to stone cell formation. In the lignin biosynthetic pathway, cinnamoyl-CoA reductase (CCR) and cinnamyl alcohol dehydrogenase (CAD), dehydrogenase/reductase family members, catalyse the last two steps in monolignol synthesis. However, there is little knowledge of the characteristics of the *CCR* and *CAD* families in pear and their involvement in lignin synthesis of stone cells. In this study, 31 *CCR*s and 26 *CAD*s were identified in the pear genome. Phylogenetic trees for *CCR*s and *CAD*s were constructed; key amino acid residues were analysed, and three-dimensional structures were predicted. Using quantitative real-time polymerase chain reaction (qRT-PCR), *PbCAD2*, *PbCCR1*, *-2* and -*3* were identified as participating in lignin synthesis of stone cells in pear fruit. Subcellular localization analysis showed that the expressed proteins (PbCAD2, PbCCR1, -2 and -3) are found in the cytoplasm or at the cell membrane. These results reveal the evolutionary features of the *CCR* and *CAD* families in pear as well as the genes responsible for regulation of lignin synthesis and stone cell development in pear fruit.

## Abstract (precis)

**Summary:** The characteristics of *CCR* and *CAD* families were systematically analyzed, and candidate members related to lignin synthesis and stone cell development were screened out in this study.

## INTRODUCTION

Pear, an important fruit species of Rosaceae, is widely distributed throughout the world. ‘Dangshan Su’ pear (*Pyrus bretschneideri* cv. Dangshan Su), originating in Dangshan County, Anhui Province, China, is a well-known diploid cultivar (2*n*=34) due to its high value in the fresh fruit market as well as for its medicinal properties (Konarska, 2013; Wu et al., 2013; Yan et al., 2014).

Factors such as the size, content and density of stone cells in pear are thought to greatly affect pear quality (Jin et al., 2013; Cheng et al., 2016). It has been shown that the lignin content of mature stone cells is approximately 18% (Lee et al., 2007). Moreover, two peaks in lignin content appear during fruit development of ‘Dangshan Su’ pear: at the peak of stone cell content and the point at which stone cell pellets reach their maximum diameter. These findings suggest that large-scale synthesis of lignin occurs to prepare material for stone cell development. As stone cell development is a process of secondary cell wall thickening and lignifying on flesh parenchyma (Cai et al., 2010; Jin et al., 2013), the formation of stone cells is closely related to the synthesis and deposition of lignin (Lee et al., 2007; Wu et al., 2013; Yan et al., 2014).

Cai et al. (2010) have shown that pear fruit lignin synthesis mainly derives from cinnamic acid and coumaric acid via the lignin pathway. Both cinnamoyl-CoA reductase [CCR (EC 1.2.1.44)] and cinnamyl alcohol dehydrogenase [CAD (EC 1.1.1.195)], enzymes belonging to the medium- or short-chain dehydrogenase/reductase (MDR or SDR) superfamilies (Pan et al., 2014; Barros et al., 2015), play key roles in specific steps of lignin monomer biosynthesis. CCR converts five types of hydroxycinnamoyl-CoA (p-coumaryl-CoA, caffeoyl-CoA, feruloyl-CoA, 5-hydroxyferuloyl-CoA and sinapoyl-CoA) into cinnamaldehyde, and CAD deoxidizes five types of cinnamaldehyde (p-coumaraldehyde, caffeyl aldehyde, coniferaldehyde, 5-hydroxyconiferaldehyde and sinapaldehyde) to the corresponding monolignol (coumaryl alcohol, caffeyl alcohol, coniferyl alcohol, 5-hydroxyconiferyl alcohol and sinapyl alcohol, respectively) (Thévenin et al., 2011). Due to their close relationship in the metabolic pathway, co-regulation of *CCR* and *CAD* might result in a more significant effect (Thévenin et al., 2011).

Various studies have shown that downregulation of *CCR* can lead to a decrease in lignin content and accumulation of monolignol precursors (Van der Rest et al., 2006; Dauwe et al., 2007; Tu et al., 2010). Known members of the *CCR* family and their functions vary greatly depending on plant species. For example, 11 *CCR*s were identified in *Populus trichocarpa*, but only *PtrCCR2* was found to be specifically expressed in xylem (Shi et al., 2010). The *Arabidopsis thaliana* genome contains 11 *CCR*s, and research has suggested that only *AtCCR1* and *AtCCR2* can encode an active cinnamoyl-CoA reductase. *AtCCR1* and *AtCCR2* play roles in plant development and pathogen defence response, respectively. *MtCCR1* and *MtCCR2* from alfalfa are both involved in lignin biosynthesis via different metabolic branches (Raes et al., 2003; Zhou et al., 2010).

The *CAD* family can be generally divided into three classes (Guo et al., 2010). All Class I *CAD* members, and also a bona fide *CAD* evolutionary branch, are associated with lignin synthesis, such as *AtCAD4*, *AtCAD5*, *BdCAD5* and *OsCAD2* (Sibout et al., 2005; Zhang et al., 2006; Bukh et al., 2012). Class II *CAD*s, known as the sinapyl alcohol dehydrogenase (SAD) evolutionary branch, are associated with stress resistance. Class III *CAD*s members may be redundant to Class I and Class III *CADs*, though their functions remain unclear (Guo et al., 2010; Yi et al., 2011).

Regardless, not all members of the *CCR* and *CAD* families are involved in lignin synthesis due to the functional diversity that occurs during evolution. Thus, it is necessary to screen and identify those *CCR* and *CAD* genes that are associated with lignin synthesis to provide effective target genes for subsequent research on the regulation of lignin metabolism. Genome sequencing of ‘Dangshan Su’ pear has been completed, and a genome size of approximately 512.0 Mb has been reported (Wu et al., 2013). However, there are few reports of pear *CCR* and *CAD* families at the whole-genome level. The first aim of the present study was to identify *CCR* and *CAD* family members in ‘Dangshan Su’ pear based on genome data. Family characteristics, including phylogenetic trees, exon-intron structures, conserved motifs, cis-elements, chromosome locations and Ka/Ks ratios, were then analysed. Analysis of catalytic sites, advanced structures and expression patterns of these members was also conducted to select candidate *PbCCR*s and *PbCAD*s related to lignin synthesis in pear fruit. Finally, the subcellular localization of the protein products of candidate genes was performed. This study elucidated the molecular characteristics, evolutionary relationships and expression patterns of the *CCR* and *CAD* subfamilies of the SDR/MDR superfamily. The findings provide candidate genes involved in regulating pear lignin synthesis and a theoretical basis for further understanding of the relationship between lignin synthesis and stone cells.

## RESULTS

### Identification and analysis of sequence attributes of *CCR*/*CAD* genes in the pear genome

Thirty-one *CCR* sequences were retained from the pear genome based on the signature motif KNWYCYGK (Zhou et al., 2010). Most of the sequences with a partial CCR-characteristic motif or mutation sites in the signature motif were named *CCR-like* (Table S1). Although their isoelectric point (pI) and protein molecular weight (MW) values range from 4.93 to 8.13 and 19.5 kDa to 110.4 kDa, respectively, the majority of PbCCRs are predicted to have an acidic pI and a MW of 34-37 kDa.

Twenty-six members of the pear *CAD* family (named as *PbCAD1-PbCAD26*, Table S2) were identified from the pear genome database based on the conserved domains of nine members of the *CAD* family in *Arabidopsis* (Raes et al., 2003).

Further analysis of their structural characteristics showed that most PbCADs have an acidic pI, though pI values do range from 5.33 to 8.77. The length of amino acid sequences encoded by *PbCAD*s varies strongly, with open reading frames (ORFs) ranging from 200 aa (PbCAD23) to 458 aa (PbCAD7). This result was consistent with the highly diverse MWs of PbCAD proteins, which range from 22.3 kDa to 49.1 kDa.

### Analysis of the gene structures, conserved motifs and phylogenetic trees of *CCR* and *CAD* family members in pear

**Fig. 1.**
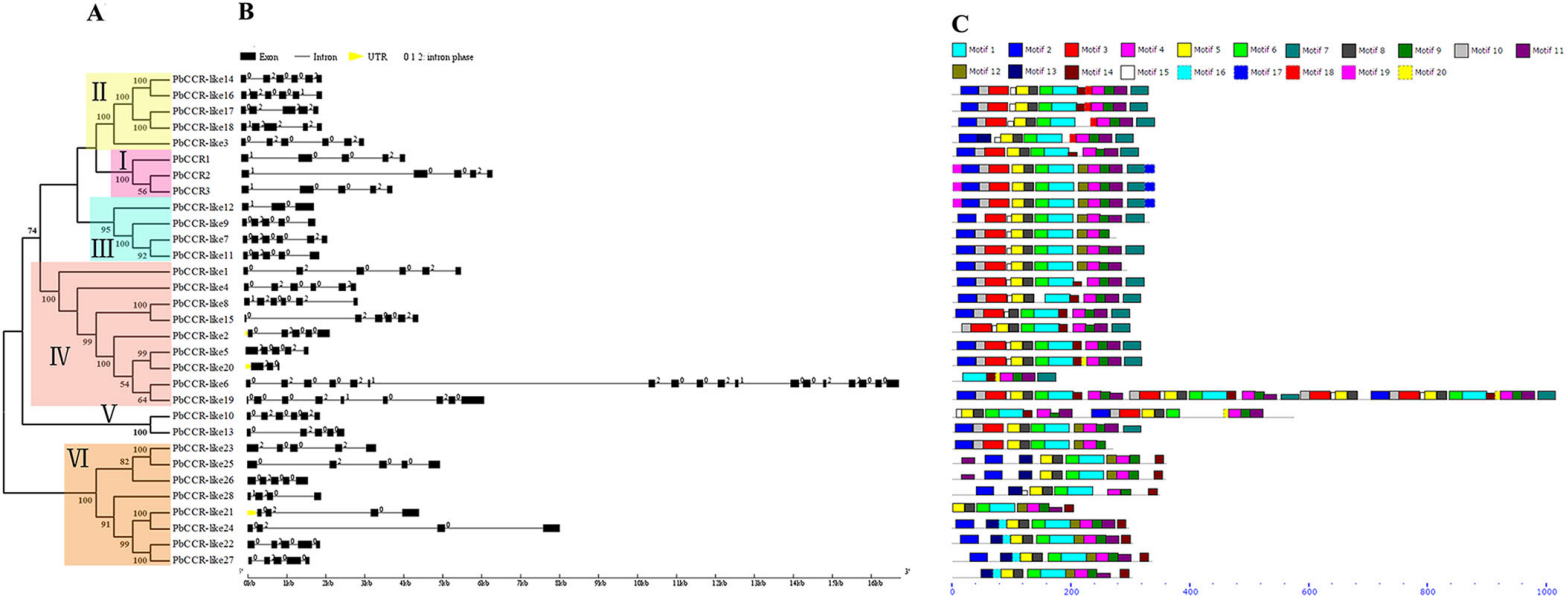
**The gene structures and conserved motifs of PbCCR/CCR-likes based on evolutionary relationships.** (A) Phylogenetic relationships of PbCCR/CCR-like proteins. (B) Exon-intron organization of *PbCCR/CCR-like* genes. (C) Distribution of twenty putative conserved motifs in PbCCR/CCR-like proteins.

**Fig. 2.**
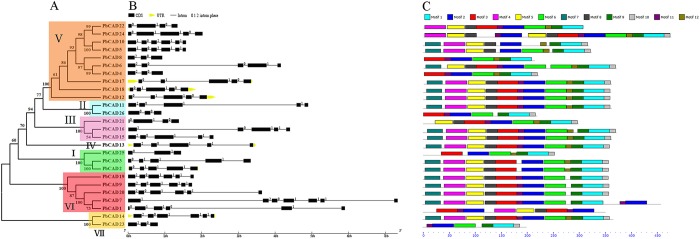
**The gene structures and conserved motifs of PbCADs based on evolutionary relationships.** (A) Phylogenetic relationships of PbCADs. (B) Exon-intron organization of *PbCAD* genes. (C) Distribution of twelve putative conserved motifs in PbCAD proteins.

To clarify evolutionary relationships among members of the *CCR* and *CAD* families in pear, phylogenetic trees were constructed using the neighbour-joining (N-J) method. As shown in Fig. 1A, 31 *PbCCR*s were assigned to six subfamilies (I-VI); among these, subfamily IV was the largest, with nine *PbCCR*s, and subfamily V was the smallest, with two members. Eleven gene pairs were formed among *PbCCR*s with higher bootstrap values (≥99), except for the lower bootstrap values of three pairs: *PbCCR2*/*3*, *PbCCR*-*like6/19* and *PbCCR*-*like7/11*. For the *CAD* family, 26 *PbCAD*s were divided into seven subfamilies (I-VII) (Fig. 2A). Eight gene pairs of *PbCAD*s were found among *PbCAD*s with higher bootstrap values (≥99), except for *PbCAD1*/*7*, *PbCAD15*/*16*.

Gene structural analysis using the online Gene Structure Display Server showed that intron numbers among the 31 *PbCCR*s are unevenly distributed and ranged from 2 to 17 (Fig. 1B). For example, there are 4 introns in subfamily I *PbCCR*s, 4 or 5 introns in subfamilies II and V, 2-5 introns in subfamily III, and 3 or 4 introns in subfamily VI. Seventeen introns were found in *PbCCR-like6* from subfamily IV. Except for two genes (*PbCCR*-*like20* and *PbCCR*-*like21*), untranslated regions (UTRs) were not found for the remaining *PbCCR*s. As shown in Fig. 2B, except for *PbCAD7* with six introns, the remaining *PbCAD*s can be divided into four classes: the first type has two introns, including *PbCAD4*, *8*, *23*, *25* and *26*; the second type has three introns with similar lengths as each exon, including *PbCAD18*, *22*; the third class has four introns, including *PbCAD2*, *3*, *6*, *11*, *13*, *15*, *16*, *17*, *18* and *24*; the last type, including *PbCAD14*, *15*, *9*, *10*, *19* and *20*, has five introns, and the length of the sixth exon is conserved. Of these *PbCAD*s, the UTRs were only found for *PbCAD2*, *12*, *13*, *14*, *17* and *18*.

Twenty and twelve conserved motifs among PbCCRs and PbCADs, respectively, were captured using the MEME online software program (Figs 1C and 2C). SMART and Pfam software were used to further analyse each motif, and the best match of each motif is summarized in Tables S3 and S4.

As shown in Fig. 1C, motifs 1-11 are present in most PbCCRs in subfamilies I to V, among which motif 20 is unique to PbCCR-like 5, 19 and 20. Both motifs 17 and 19 are unique to subfamily I, and motif 18 is unique to subfamily II. Furthermore, subfamily VI PbCCRs do not contain motif 3. These results indicate that most PbCCRs in the same subfamily have highly similar gene structures and motifs, which supports their close evolutionary relationship and the reliability of the phylogenetic trees constructed. For most of the 26 PbCAD sequences, motifs 4, 5 and 7 were found at the N-terminal region, whereas motifs 1, 9 and 10 are located at the C-terminal region (Fig. 2C). In addition, motif 2, encoding an ADH_zinc_N domain, was found in all 26 PbCADs. Motif 4 or 5, encoding an ADH_N domain, was found in 21 PbCADs. Only PbCAD1 does not contain motif 1, the function of which is unclear.

### Chromosomal distribution and duplication of *PbCCR*/*PbCAD* genes

To clarify the chromosomal distribution and expansion mechanism at the genomic scale for members of the *PbCCR* and *PbCAD* families, chromosomal location analysis was performed. The results showed that the 31 *PbCCR*s are randomly distributed on 11 chromosomes (Fig. S1), among which chromosome 2 harbours the maximum number of genes, at 5 *PbCCR*s, with chromosomes 6, 7 and 17 containing 4 genes each. Three genes are located on chromosome 11 and 2 each on chromosomes 9 and 13. Twenty-five of 26 *PbCAD*s are found at the terminal regions of chromosomes 5, 10, 13, 14, 15 and 17 (Fig. S2). In addition, 6 *PbCAD*s are carried on chromosomes 10 and 13, 7 on chromosome 15, and 4 on chromosome 17.

Additionally, except for one *CCR* or *CAD* gene on chromosomes 1, 3, 5, 8, 14 and 16, most *PbCCR*s and *PbCAD*s are closely distributed in the form of gene clusters at the end regions of chromosomes.

According to the phylogenetic tree and chromosomal localization analyses, five pairs of *PbCCR*s (*PbCCR10*/*13*, *PbCCR23*/*25*, *PbCCR22*/*27*, *PbCCR8*/*15* and *PbCCR21*/*24*) and two pairs of *PbCAD*s (*PbCAD2*/*3* and *PbCAD5*/*10*) were found to be associated with gene duplication events. In addition to tandem duplication events among *PbCCR10/13*, *PbCAD2/3* and *PbCAD5/10*, fragment replication events were found for other gene pairs. As shown in Table S5 and Fig. S3, the non-synonymous substitution rates (Ka) of five pairs of *PbCCRs* and two pairs of *PbCAD*s were lower than their synonymous substitution rates (Ks), suggesting that Ks is more advantageous than Ka during evolution. Furthermore, the Ka/Ks ratios of these seven replication events are <1, indicating no obvious differentiation of gene function through functional purification and selection (Ouyang et al., 2009; Kong et al., 2013; Wei et al., 2013).

### Analysis of cis-regulatory elements in *PbCCR*s and *PbCAD*s

In this study, the sequence 2000 bp upstream from the initiation codon in *PbCCR*s and *PbCAD*s was analysed to understand the regulatory mechanisms that control expression of these genes.

As shown in Tables S6 and S7, most members of the *PbCCR* and *PbCAD* families contain the *spl* light-responsive element. Interestingly, 29 *spl* elements were identified in the upstream regulatory sequence of *PbCAD2*, suggesting that expression of *PbCCR*s and *PbCAD*s is closely related to light.

AC elements are usually identified in promoter regions of genes involved in phenylpropanoid pathways and are thought to be cis-regulatory elements for binding by MYB and other transcription factors to regulate phenylpropanoid metabolism (Raes et al., 2003; Zhao and Dixon, 2011). In our analysis, PLANT CARE software identified AC elements in the promoters of 10 *PbCCR*s (*PbCCR1*, *-2* and *PbCCR-like3*, *-6*, *-11*, *-17*, *-21*, *-22*, *-25*, *-27*) and six *PbCAD*s (*PbCAD2*, *-3*, *-4*, *-8*, *-14*, *-17*), suggesting that these genes may be involved in the metabolism of phenylpropanoids under regulation by MYB transcription factors.

Other cis-regulatory elements such as the ABRE, TCA-element and TGACG-motif are present in most *PbCCR*s and *PbCAD*s, among which the TGACG-motif was found in all *PbCAD*s examined. These three elements are related to the signal pathways of abscisic acid (ABA), jasmonic acid (SA) and methyl jasmonic acid (MeJA), respectively. Because ABA, SA and MeJA are important signalling molecules in plant responses to stress, it is likely that most of members of the *PbCCR* and *PbCAD* families are involved in responses to biotic and abiotic stresses. Additionally, the promoters of *PbCCR*s and *PbCAD*s contain many HSE and LTR elements, indicating that they are also subject to temperature stress regulation.

### Evolutionary analysis of *CCR*/*CAD* proteins from pear and other plants

**Fig. 3.**
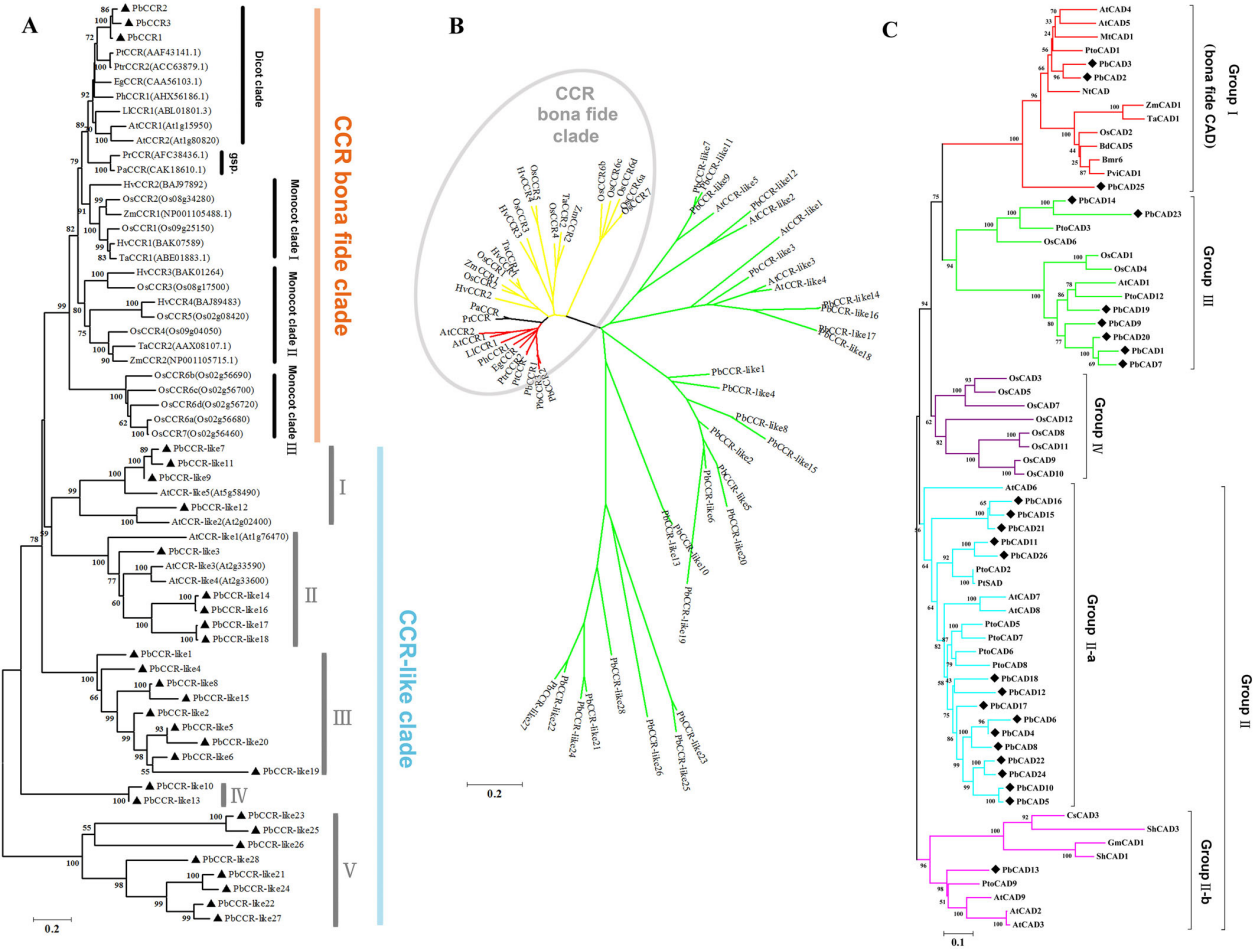
**Phylogenetic tree of *CCR* and *CAD* protein sequences of various plants.** (A) Traditional phylogenetic tree of CCR protein sequences of various plants. Black triangles indicate PbCCRs. (B) Radiation tree of CCR protein sequences of various plants. (C) Phylogenetic tree of CAD proteins sequences of various plants. Black diamonds indicate PbCADs. A and B are phylogenetic trees of 31 CCRs from pear with 32 CCRs from other species. C is a phylogenetic tree of 26 CADs from pear with 38 CADs from other species. Protein sequences were aligned and a phylogenetic tree was constructed by the neighbour-joining (N-J) method using MEGA5.1 software. The species name and the accession number of sequences used to construct the phylogenetic tree are listed in Table S10.

To clarify the relationship between CCRs in different species and to predict the biological function of members of the *PbCCR* family, 31 PbCCRs in pear and 32 CCRs derived from other species were used to construct a phylogenetic tree (Fig. 3A,B). The phylogenetic tree could be obviously divided into two major categories: a bona fide CCR clade and a CCR-like clade. All CCR-likes of *Arabidopsis* and pear clustered together, and the remaining CCRs grouped into the bona fide CCR clade (Fig. 3B), indicating that functional differentiation may have occurred between CCR and CCR-like proteins. As shown in Fig. 3A, the bona fide CCR clade was divided into dicotyledon, monocotyledon and gymnosperm clades depending on the divergent species. PbCCR1, PbCCR2 and PbCCR3 were classified into the dicotyledon clade, in which some CCRs such as AtCCR1, AtCCR2 and PtrCCR2 have been shown to be related to lignin synthesis (Shi et al., 2010; Barros et al., 2015; Van Parijs et al., 2015). The three PbCCRs also clustered into this clade, suggesting that these pear proteins are likely to participate in lignin metabolism.

In addition, ZmCCR1, TaCCR1, ZmCCR2 and TaCCR2 in monocotyledon clade I or II are related to lignin metabolism (Van Parijs et al., 2015). Monocotyledon clade III contains all the CCRs of *Oryza sativa*, of which OsCCR7 is known to be catalytically active against feruloyl-CoA (Kawasaki et al., 2006). Moreover, inhibition of PrCCR, in the gymnosperm clade alters the composition of the plant cell wall and lignin content (Wagner et al., 2013). In general, members of each subfamily of the bona fide CCR clade have functions associated with lignin metabolism.

The CCR-like clade can be divided into five subfamilies (I-V). AtCCR-likes are distributed into subfamilies I and II, and PbCCR-likes into subfamilies III-V. Various isoforms of CCR may play roles in compensatory or autonomic regulation and other metabolic pathways (Zhou et al., 2010; Pan et al., 2014).

A CAD phylogenetic tree of different species origins was constructed using MEGA 5.1 software to understand relationships among different species (Fig. 3C). Sixty-four CADs were clearly divided into four subgroups (I-IV); AtCADs were classified into four groups in this study. This result was consistent with that of Raes et al. (2003) and confirmed the credibility of the phylogenetic tree. Group I (the bona fide *CAD* clade) contains CADs derived from dicotyledons and monocotyledons, and its members are associated with lignin synthesis. For example, AtCAD4, AtCAD5, BdCAD5, OsCAD2, TaCAD1, PviCAD1, NtCAD, MtCAD1, Bmr6 and PtoCAD1 catalyse the conversion of cinnamaldehyde to monolignol, and are *in vitro*. Moreover, further proof that these proteins are involved in lignin biosynthesis is derived from mutant and genetic transformation experiments (Kim et al., 2004; Zhang et al., 2006; Sattler et al., 2009; Ma, 2010; Barakate et al., 2011; Saathoff et al., 2012; Zhao et al., 2013; Van Parijs et al., 2015). Therefore, PbCAD2, -3 and -25, which clustered in this category with high bootstrap values, are likely to be involved in lignin metabolism in pear.

Members of the *CAD* family in Group II are composed of CADs derived from three dicotyledonous plants: pear, poplar and *Arabidopsis*. Group II can be divided into two subcategories: Group II-a and Group II-b. AtCAD7 and AtCAD8 in Group II-a and CsCAD3 in Group II-b are associated with plant resistance, but exhibit very low catalytic activity with lignin precursors (Kim et al., 2004, 2007; Deng et al., 2013). It was inferred that PbCADs of Group II are involved in the stress resistance process in pear and are not responsible for synthesis of lignin. Guo et al. (2010) reported that some of these members are similar to SAD (Li et al., 2001). For example, PtoCAD2 shows 99.4% identity to aspen SAD (PtSAD) (Chao et al., 2014). PbCAD11, PbCAD26, PtSAD and PtoCAD2 clustered into one clade with high bootstrap values, indicating that they may be orthologous to SAD and may have higher catalytic efficiency toward sinapaldehyde (Li et al., 2001; Bomati and Noel, 2005; Chao et al., 2014). In Group II-b, PbCAD13 grouped with AtCAD2, AtCAD3, AtCAD9 and PtoCAD9, which are not associated with lignin metabolism and show lower catalytic activity (Kim et al., 2004, 2007; Chao et al., 2014).

Group III is composed of CADs from pear, rice and poplar and *Arabidopsis*. Among them, PtoCAD3 and OsCAD6 are not involved in lignin metabolism (Tobias and Chow, 2005; Chao et al., 2014). Thus, PbCAD14 and PbCAD23 in Group III may also not be critical for lignin biosynthesis in pear. AtCAD1 plays a partial role in the synthesis of lignin in *Arabidopsis* (Eudes et al., 2006). Thus, it remains to be determined whether PbCADs (PbCAD1, -7, -9, -19 and -20) are related to lignin synthesis. All members of Group IV are CADs from rice, consistent with a previous study (Hirano et al., 2012).

We constructed two phylogenetic trees with the same sequences using ML (maximum likelihood) and MP (maximum parsimony) methods to further support the reliability of the N-J phylogenetic tree (Figs S4, S5, S6 and S7). We found that the evolutionary relationships revealed by these two types of phylogenetic trees are consistent with those of the N-J phylogenetic tree; moreover, the members in the bona fide clade of these three phylogenetic trees are identical. This evidence is sufficient to show that construction of the N-J phylogenetic tree was credible.

### Sequence and three-dimensional structure analysis of pear *CCR*/*CAD* family members

**Fig. 4.**
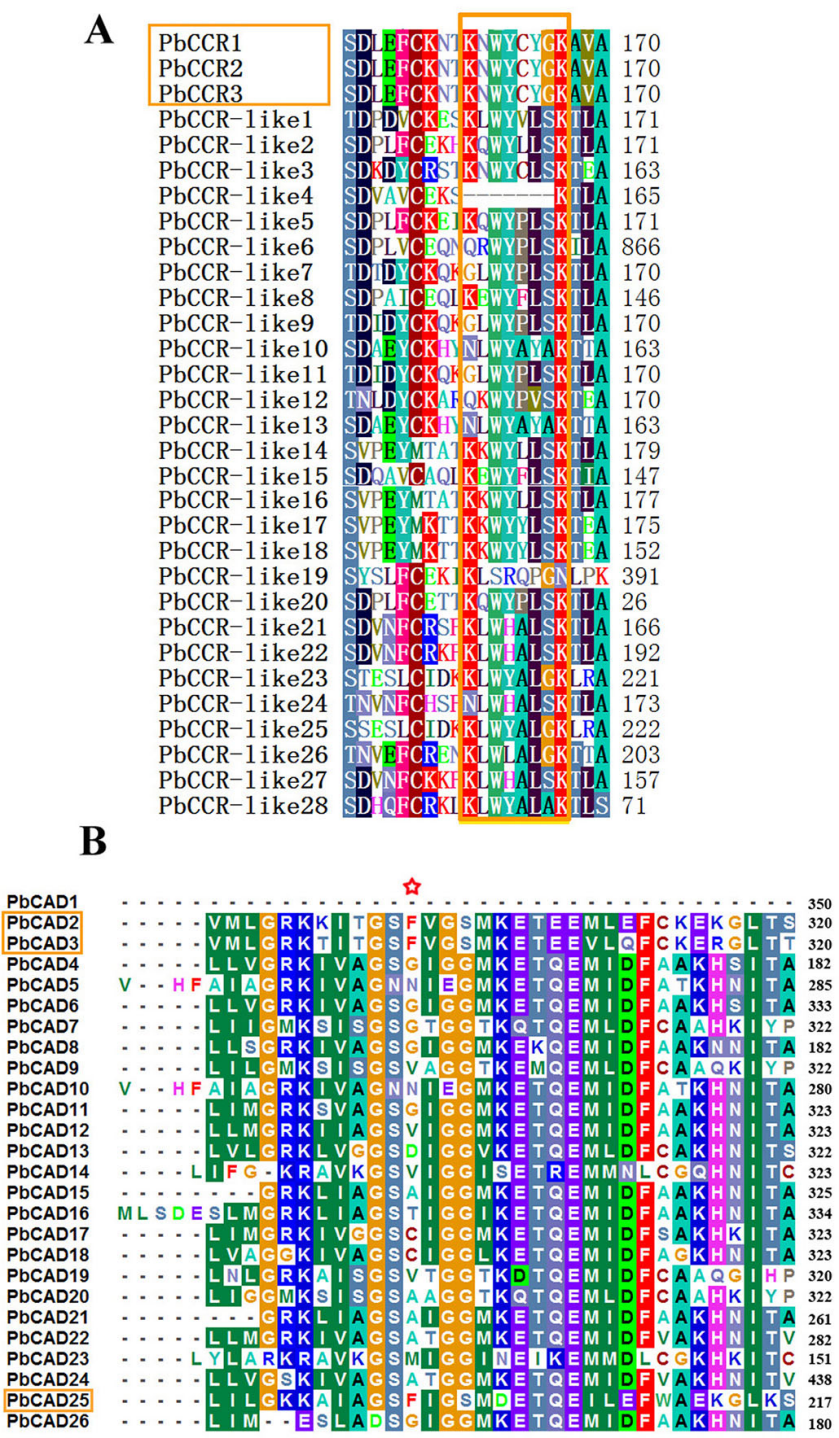
**Multiple sequence alignment of PbCCR/CCR-likes and PbCADs.** (A) Partial sequence alignment of PbCCR/CCR-likes. The conserved motif is marked by a yellow rectangle. (B) Partial sequence alignment of PbCADs. The key determinant residue site is marked with a star.

The conserved motif KNWYCYGK is a signature of CCR, the three-dimensional conformation of which is directly related to the recognition of coenzyme A (Pan et al., 2014). As shown in Fig. 4A, this motif is highly conserved in PbCCR1, -2 and -3, suggesting that the three PbCCRs possibly interact with hydroxycinnamoyl-CoA in lignin metabolism. The motifs of PbCCR-like proteins contain diverse polymorphisms, indicating their functional differentiation. Some members exhibit a partial deletion of the conserved motif, which may have been caused by loss during *CCR* family expansion in the genome.

Bomati and Noel (2005) found the phenylalanine (Phe) residue at position 302 of bona fide CADs to be important for their activity. Therefore, BioEdit software was used to align PbCAD sequences in this study (Fig. 4B). The corresponding site is Phe for PbCAD2, -3 and -25 only, suggesting that these proteins may involve in pear lignin synthesis.

First, we compared CCR and CAD proteins between *Arabidopsis* and pear using the online software Sequence Manipulation Suite to predict lignin-specific PbCCRs and PbCADs (Figs S8 and S9). The results showed that PbCCRs and PbCADs have 13–75% and 11–80% identity (Iden) and 18–88% and 29–88% similarity (Sim) to *Arabidopsis* proteins, respectively. Among them, PbCCR1, -2, -3 and PbCAD2, -3 show higher similarity and identity with AtCCR1 and -2 and AtCAD4 and -5, respectively.

**Fig. 5.**
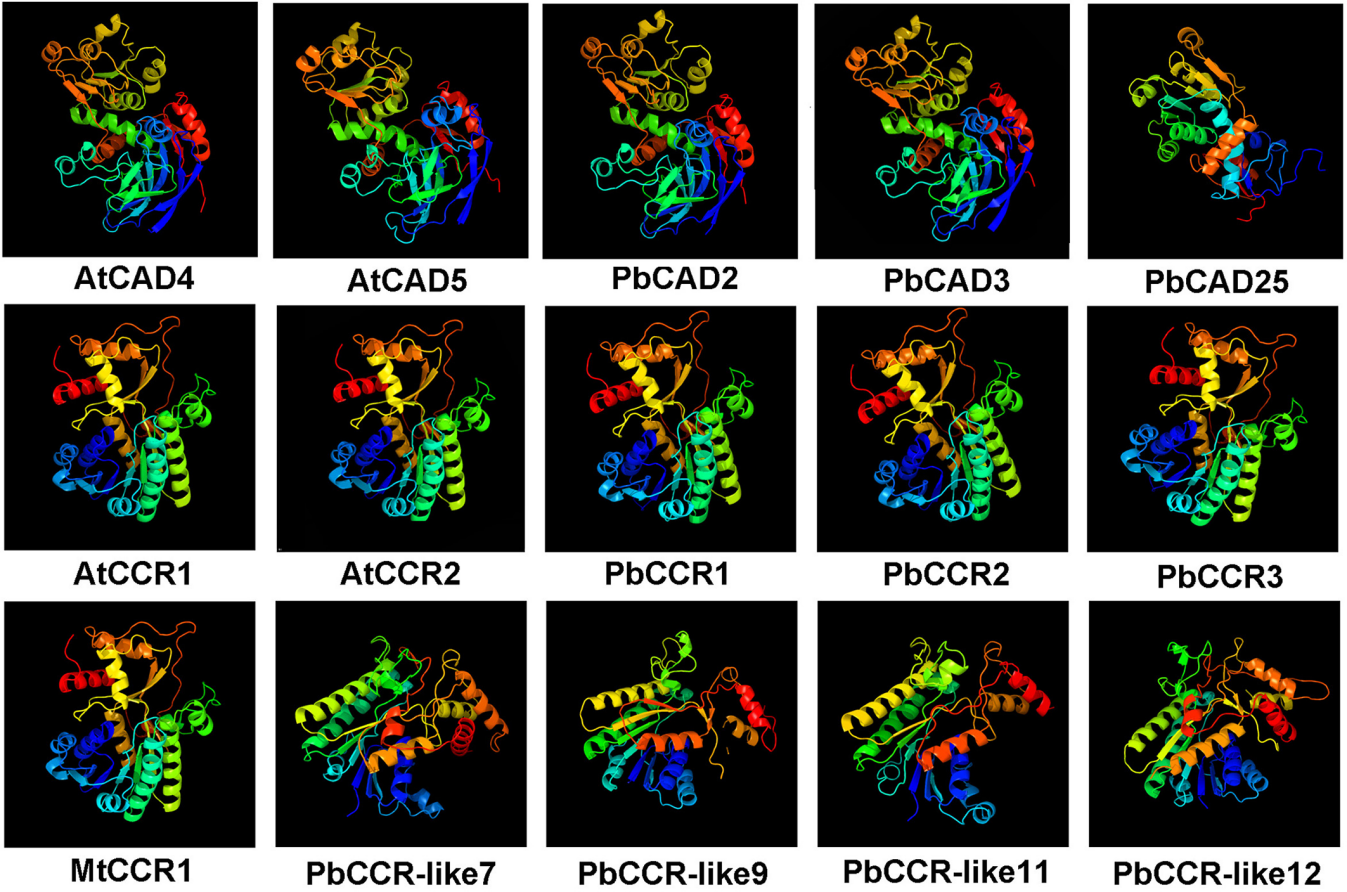
**Predicted three-dimensional structures of CCR/CCR-likes and CADs from pear and other species.** At, *Arabidopsis thaliana*; Mt, *Medicago truncatula*. AtCCR1, AtCCR2, AtCAD4, AtCAD5 and MtCCR1 have been proven to be responsible for lignin biosynthesis.

Subsequently, the three-dimensional structures of the six proteins (PbCCR1, -2, -3 and PbCAD2, -3, -25) in the bona fide clade were predicted and compared with other lignin-specific CADs and CCRs reported to be related to lignin synthesis. As shown in Fig. 5, the three-dimensional structures of PbCAD2 and -3 exhibit high similarity with those of AtCAD4 and -5, indicating that they may bind to the same substrate; in contrast, the three-dimensional structure of PbCAD25 differs greatly from those of the four above proteins. The three-dimensional structures of PbCCR1, -2 and -3 are highly consistent with those of bona fide CCRs from *Arabidopsis* (AtCCR1, AtCCR2) and alfalfa (MtCCR1), suggesting that they may have similar functions in lignin synthesis. The four PbCCR-like proteins (PbCCR-like7, -9, -11, -12) had significantly different structures from those in the CCR clade, which is consistent with the results of the phylogenetic relationship analysis.

Five members (PbCCR1, -2, -3 and PbCAD2, -3) of the *PbCCR* and *PbCAD* families were preliminarily identified as lignin-specific CCRs and CADs because they have high similarity with the amino acid sequence and three-dimensional structure of other lignin-specific CCRs and CADs and possess many key amino acid residues that are conserved among other species (Tables S8 and S9). Although belonging to the bona fide clade, PbCAD25 lacks approximately 100 aa at the N-terminal region, which results in a change in its three-dimensional structure. Additionally, the deletion of two substrate-binding sites and a Zn^2+^-binding site in PbCAD25 might lead to a change in its catalytic function (Table S9). Thus, PbCAD25 was excluded from the lignin-specific CADs.

### Analysis of changes in stone cells and lignin content during fruit development in ‘Dangshan Su’ pear

**Fig. 6.**
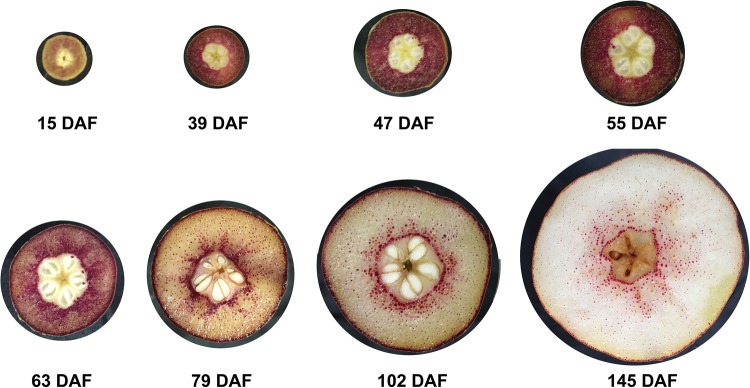
**Stone cell staining of ‘Dangshan Su’ pear at different developmental stages.** Transverse sections of fruit at eight developmental stages were stained using the Wiesner method. DAF, days after flowing.

To understand dynamic changes in stone cell and lignin contents during fruit development in ‘Dangshan Su’ pear, the stone cells in pear fruits from eight periods after flowering were stained and observed to determine their distribution and lignin content. The lignin components of stone cells stained with phloroglucinol appear as a purplish-red colour (Fig. 6). As shown in Fig. 6, there was almost no stone cell formation in fruits at 15 days after flowering (DAF). During the period from 39 to 55 DAF, rapid formation of stone cells was observed in stained fruit flesh. From 63 to 145 DAF, it was obvious that the stone cells were primarily distributed near the centre of the fruit. As stone cell formation decreased with development, the density of stone cells in the fruit centre was higher than that in the outer region. Moreover, stone cell formation first increased and then decreased during fruit development, with a peak content from 39 to 55 DAF.

Stone cell development is a process associated with secondary thickening and lignification of parenchyma cells; indeed, lignin is an important raw material for stone cell formation. Thus, we assessed changes in lignin content during pear fruit growth (Fig. S10). The results showed that the lignin content was very low at the early stage of fruit development, then increased from 39 DAF and was maintained at a higher level from 47 to 63 DAF, and then gradually decreased, which was consistent with the dynamic change in the stone cell content.

### Expression patterns of *PbCCR*/*CAD* genes

*CCR* and *CAD* exist in gene families in the higher plant genome (Pan et al., 2014). In addition to many members (bona fide *CCR*/*CAD*) that have major functions, other members may also play roles in compensatory effects, such as some *CCR-like* members, *PtoCAD2* and *AtCAD1* (Eudes et al., 2006; Zhou et al., 2010; Chao et al., 2014). Therefore, closely related *PbCAD*s (*PbCAD1*, *-7*, *-9*, *-11*, *-19*, *-20* and *-26*) and members of the *PbCCR-like* I clade were selected to analyse temporal and spatial patterns of gene expression.

**Fig. 7.**
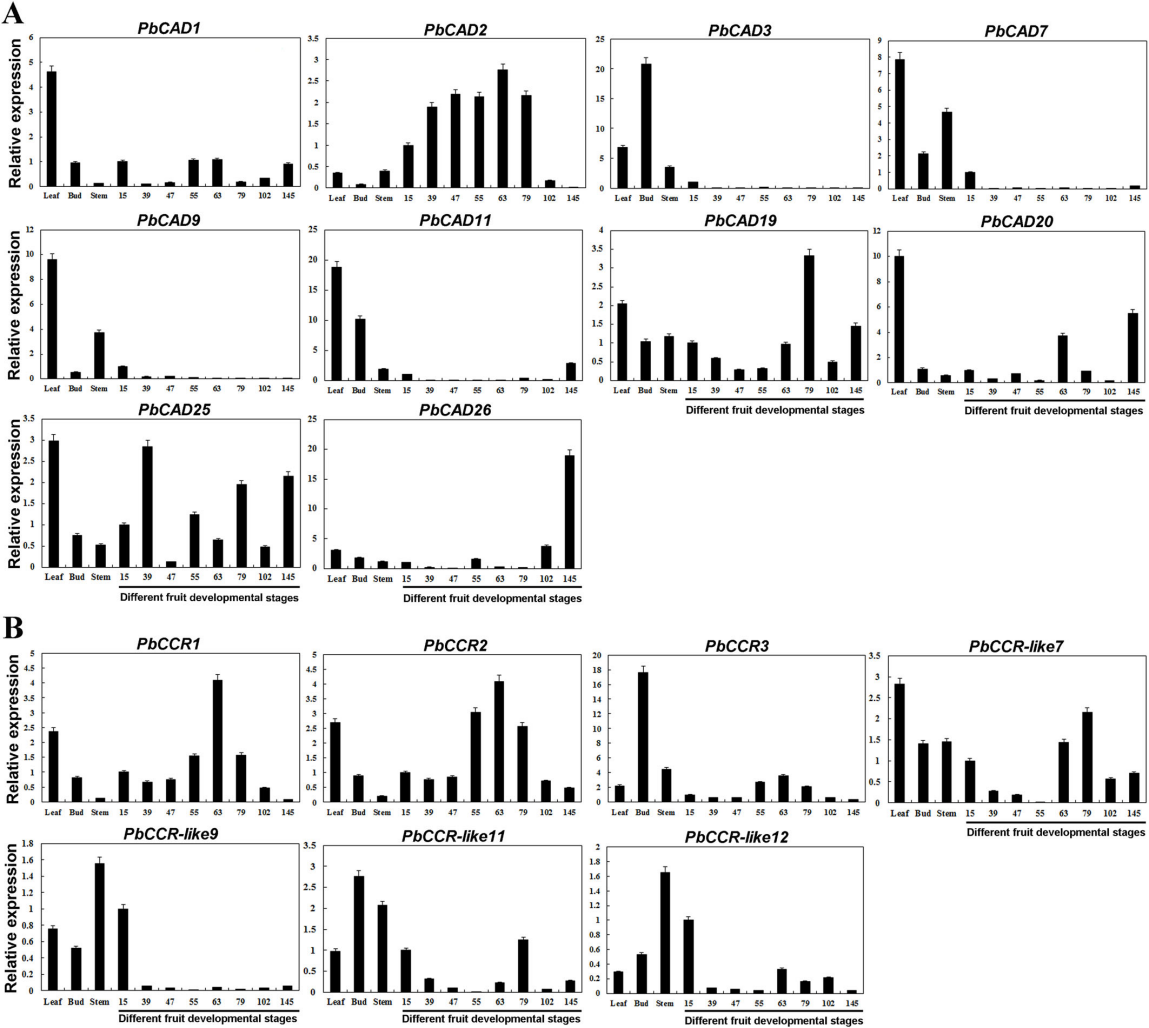
**Expression patterns of *PbCCR*/*CAD* genes.** (A) Spatiotemporal expression patterns of *PbCAD* genes. (B) Spatiotemporal expression patterns of *PbCCR* genes. The ordinate represents the relative expression level of genes. The abscissa represents different tissues of pear and developmental stages of pear fruit. 15 to 145, fruits from 15 to 145 DAF. Error bars represent the variability of qRT-PCR results from three replicates.

As shown in Fig. 7, these selected genes displayed non-specific expression patterns in different developmental stages of pear fruit, buds, stems and leaves. *PbCAD2* was predominantly expressed in fruit, with an expression level that was significantly higher than that in shoots, stems and leaves. This higher expression level was maintained from 39 to 79 DAF, suggesting that *PbCAD2* may be responsible for the increased lignin synthesis and stone cell development during this period. Although *PbCAD3* belongs to the bona fide *CAD* clade, its expression level was much weaker in fruits than in buds. Therefore, *PbCAD3* may not be responsible for lignin biosynthesis in fruit, or it may be induced under specific circumstances such as insect attack or other damage (Rong et al., 2016). Similar expression levels were found for *PbCAD7* and *-9* in fruit, and the trends of *PbCAD1*, *-19* and *-25* expression were not significantly correlated with lignin and stone cell contents. Although *PbCAD11* and *PbCAD26* were found to be closely related to *PtSAD*, their expression levels were inconsistent with the peak of lignin content, with up-regulated expression only in the fruit-ripening stage (145 DAF). In addition, *PbCAD19*, *-20*, *-25* and *-26* were also significantly up-regulated during fruit ripening, which may be related to tissue maturation or ageing (Singh et al., 2010; Gabotti et al., 2015).

For *PbCCR*s, three genes (*PbCCR1*, *PbCCR2* and *PbCCR3*) in pear fruit showed an expression pattern of significant up-regulation from 55 to 79 DAF, which was similar to the trends in lignin and stone cell contents (Fig. 7). These results suggest that they are closely related to lignin synthesis and stone cell development. In addition, expression of *PbCCR1* and *PbCCR2* was also higher in leaves, indicating their function in leaf development (Xue et al., 2015). Expression of *PbCCR3* was higher in buds than that in other tissues, which suggests that *PbCCR3* plays diverse roles in tissue development. The expression levels of *PbCCR-like7*, *-9*, *-11* and *-12* were all higher in shoots, stems, leaves and in early fruit developmental stages (15 DAF). Because the expression levels of *PbCCR-like7* showed an opposite trend to that of lignin and stone cell contents and because the level of *PbCCR-like9* expression was significantly weaker than that of the other genes during fruit development, it was inferred that *PbCCR-like7* and *-9* are not likely to be involved in lignin biosynthesis or stone cell formation (Fig. 7).

In summary, qRT-PCR, phylogenetic tree, key amino acid residue sites, sequence similarity, and protein structure prediction analyses showed that *PbCAD2* and *PbCCR1* and *-2* may have major roles in pear lignin biosynthesis and that *PbCCR3* may have a complementary role. *PbCAD25* showed a poor correlation with changes in fruit lignin content; therefore, this gene was not associated with fruit lignin synthesis.

Interestingly, the lignin content in fruit gradually decreased from 79 DAF, whereas the expression levels of *PbCAD2* and *PbCCR1*, *-2* and *-3* were still higher in this period, which indicates that these genes may be involved in lignin monomer synthesis as well as the synthesis of other metabolites, such as sinapoyl malate and lignan (Barros et al., 2015; Pascual et al., 2016).

### Subcellular localization analysis of candidate genes for pear lignin synthesis

**Fig. 8.**
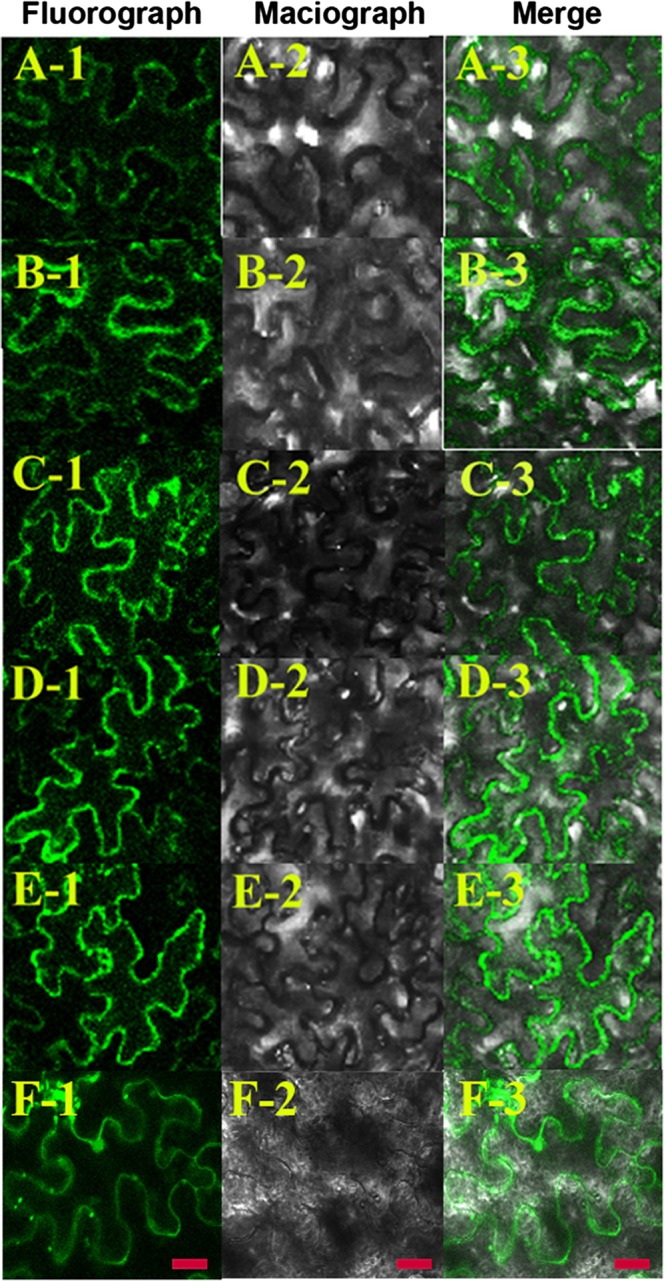
**Subcellular localization of lignin-specific PbCCRs and PbCADs.** Subcellular localization of pCAMBIA1304-*PbCCR1*; B1-3, subcellular localization of pCAMBIA1304-*PbCCR2*; C1-3, subcellular localization of pCAMBIA1304-*PbCCR3*; D1-3, subcellular localization of pCAMBIA1304-*PbCAD2*; E1-3, subcellular localization of pCAMBIA1304-*PbCAD3*; F1-3, subcellular localization of pCAMBIA1304. Scale bar: 10 μm.

Through subcellular localization using transient expression driven by the *CaMV*35S promoter in *Nicotiana benthamiana* leaves, five proteins (PbCAD2, -3 and PbCCR1, -2, -3) were detected in the cytoplasm and at the cytoplasmic membrane, in contrast to the random distribution of green fluorescent protein (GFP) throughout cells transformed with the empty plasmid (Fig. 8). Both *CCR* and *CAD* are structural genes involved in monolignol synthesis. Moreover, monolignols are mainly synthesized in the cytoplasm and then transported outside the membrane for polymerization (Barros et al., 2015), which is consistent with the subcellular localization results.

## DISCUSSION

The developmental process of stone cells in pear fruit is closely related to the metabolism of lignin, the content and structure of which have important influences on the formation of stone cells (Yan et al., 2014; Barros et al., 2015). CCR and CAD are enzymes that catalyse the last two steps of lignin monomer synthesis. Suppression of their expression can reduce the total lignin content as well as its structure, such as the G/S ratio, and allow for ferulic acid, hydroxy cinnamic aldehyde and other lignin precursors to be incorporated into the lignin polymer, resulting in lignin with looser structural features which are easily degraded (Thévenin et al., 2011; Wagner et al., 2013; Jin et al. 2013; Wilkerson et al., 2014). Thus, CCR and CAD are two important targets. Inhibiting their expression in pear fruit may prevent secondary thickening of the cell wall and reduce the content and size of stone cells.

With the completion of genome sequencing of several species, screening and identification of the two families of lignin biosynthesis-related members have been performed in *P. trichocarpa*, *O. sativa*, *Lolium perenne* and other plants (Shi et al., 2010; Hirano et al., 2012; Carocha et al., 2015; Van Parijs et al., 2015). However, there is no such systematic study of *CCR* and *CAD* families in pear. In this study, 31 *CCR*s and 26 *CAD*s members were identified in the pear genome. More *PbCCR*s were found in pear than in *Arabidopsis* (11), *L. perenne* (13), *P. trichocarpa* (11) and *Eucalyptus grandis* (27) but fewer than in *Malus pumila* (47). There were also more *PbCAD*s in pear than in *Arabidopsis* (9), *L. perenne* (6) and *P. trichocarpa* (16) and fewer than in *E. grandis* (46) and *M. pumila* (50) (Raes et al., 2003; Shi et al., 2010; Wu et al., 2013; Carocha et al., 2015; Van Parijs et al., 2015). These results suggest the occurrence of different replication events and the existence of different expansion mechanisms among *CCR* and *CAD* families of different species.

Via functional verification experiments that reflect the accuracy of phylogenetic tree functional prediction, studies of *Liriodendron chinense*, *P. trichocarpa*, *Brachypodium distachyon* and *O. sativa* have verified that the members classified into the bona fide clade of phylogenetic trees play a primary role in lignin biosynthesis (Shi et al., 2010; Yi et al., 2011; Bukh et al., 2012; Hirano et al., 2012; Carocha et al., 2015). We selected six candidate genes (*PbCCR1*, *-2*, *-3* and *PbCAD2*, *-3*, *-25*) to further analyse which genes have a close relation to lignin synthesis-related CCRs/CADs.

Analysis of the expression patterns of selected members of the *PbCCR* family revealed *PbCCR-like7* and *PbCCR-like11* to be highly expressed in leaves and buds, respectively (Fig. 7). However, *PbCCR-like9* and *PbCCR-like12* were found to be highly expressed in the stem and weakly expressed in the fruit. Similar expression patterns were found in maize and wheat. Although expression was usually found in roots or leaves, *ZmCCR2* and *TaCCR2* were reported to be up-regulated throughout the entire plant under stress (Van Parijs et al., 2015). Additionally, ethylene-responsive elements (EREs) are present in the 5′ UTR of *PbCCR-like7*, *-9* and *-12* (Table S6), suggesting that these genes play an important role in ageing and stress responses. We also identified three elements that respond to low temperature (LTR) in the upstream UTR of *PbCCR-like11* (Table S6), which may be related to plant antifreeze. This evidence indicates these genes function in certain compensatory roles or in response to stress. The expression trends of *PbCCR1*, *-2* and *-3* were correlated with the accumulation of fruit stone cell and lignin contents, suggesting that their functions are related to lignin synthesis in pear fruit (Fig. 7).

Interestingly, we found that 87% and 73% members of the *CCR* and *CAD* families harbour *Spl* elements, respectively, indicating that these two family members are widely regulated by light. Fruit bagging can affect the content of stone cells in pear cultivation, which is likely because the bag alters the light intensity. This directly affects expression of *CCR* and *CAD* members with light response elements in fruit and changes lignin metabolism, which ultimately affects the development of stone cells (Tao et al., 2015; Wang et al., 2017b). Although light cannot reach the middle region of pear fruit, the effect of light may be transduced from photoreceptors in the peel, similar to what occurs in underground tissues (roots, storage roots), to internal cells by phytochrome and cryptochrome. Such signalling directly affects transcription of lignin metabolism-related transcription factors and structural genes and ultimately influences lignin monomer synthesis and stone cell development (Hemm et al., 2004; Hou et al., 2010; Zoratti et al., 2014). In addition, light indirectly affects lignin synthesis in pear fruit. For example, light can change saccharide metabolism in fruit; saccharide not only provides precursors and energy for lignin metabolism but also acts as a signalling molecule. Thus, the light signal can indirectly control lignin synthesis through saccharide metabolism (Rogers et al., 2005; Pascual et al., 2016).

Due to the loss of the key amino acid residues and subsequent conformational change compared to other bona fide CADs (Table S9; Fig. 4B), PbCAD25 is suggested to be unable to bind to cinnamaldehyde. Furthermore, an RY-element, which is involved in seed-specific expression, was found in the upstream UTR of *PbCAD25*, instead of AC elements related to lignin synthesis in *PbCAD2* and *-3* (Table S7), suggesting that *PbCAD25* may not play a major role in fruit. Moreover, as its expression was not correlated with the lignin content, it does not appear to be related to fruit lignin biosynthesis. Although PbCAD3 possesses the characteristics of bona fide CADs, its expression level in pear fruit was very low; accordingly, it was considered a key enzyme responsible for lignin synthesis in other plant parts. Only PbCAD2 contains the same substrate-binding site as other bona fide CADs, and its expression pattern was consistent with lignin and stone cell contents in pear fruits (Fig. 7).

In *Arabidopsis*, *AtCAD4* (*AtCAD-C*) and *AtCAD5* (*AtCAD-D*) are both responsible for lignin synthesis. *AtCAD5* is primarily expressed in roots and *AtCAD4* predominantly in flowers and leaves. Although changes in the lignin content of the single mutants *cad-c* or *cad-d* was not obvious, the double mutant *cad-c cad-d* showed a phenotype of stem lodging, with 40% decreased lignin content compared to wild type (Sibout et al., 2005; Thévenin et al., 2011). However, single mutants of *CAD*, such as the maize mutant *bmr1* and the alfalfa mutant *cad1-1*, showed decreased lignin contents. This result suggests that there is functional complementation between *AtCAD4* and *AtCAD5* in *Arabidopsis* and that *CAD* family members have no functional redundancy in maize and alfalfa (Halpin et al., 1998; Zhou et al., 2010). Therefore, it must be experimentally verified whether there is complementation, although the expression sites of *PbCAD2* and *PbCAD3* are different.

Additionally, two PbCADs (PbCAD11, -26) with a close genetic relationship to PtSAD were identified by our phylogenetic tree (Fig. 3C). PtSAD is thought to be the key enzyme for synthesis of S-lignin in angiosperms (Li et al., 2001). However, our qRT-PCR results showed that expression of *PbCAD11* and *PbCAD26* lagged behind lignin accumulation during pear fruit development, indicating their diversified functions rather than specific responsibility for S-lignin synthesis (Bukh et al., 2012; Chao et al., 2014).

In conclusion, we analysed the characteristics of *CCR* and *CAD* family members, including sequence features, gene structures, conserved motifs, chromosome locations, replication events, cis-regulatory elements and evolutionary relationships. Candidate genes related to lignin metabolism were determined using a phylogenetic tree, multiple sequence alignment, comparison of the key amino acid residues and three-dimensional modelling. *PbCAD2* and *PbCCR1*, *-2* and *-3* were selected as target genes for lignin synthesis in pear fruits. These results provide the foundation for further research on the roles of *CCR* and *CAD* in lignin metabolism and stone cell development in pear fruits.

## MATERIALS AND METHODS

### Identification and classification of *CCR*/*CAD* family genes in pear

Diploid pear genomic data were downloaded from a website (http://gigadb.org/dataset/100083), and a local protein database was established using DNATOOLS software (Wang et al., 2017a). Two *Arabidopsis* CCRs (At1g15950 and At1g80820) determined to be relevant to the biosynthesis of lignin precursors (Thévenin et al., 2011) were used as query sequences for searching against the local protein database to obtain candidate *CCR* sequences in pear using BLASTp program (E-value=0.001). The sequences in the screening were confirmed using BLASTp from the National Center for Biotechnology Information (www.ncbi.nlm.nih.gov/) and Conserved Domain Search (www.ncbi.nlm.nih.gov/Structure/cdd/wrpsb.cgi) to exclude sequences with no conserved CCR domain. The signature domains ADH_zinc_N (PF00107) and ADH_N (PF08240) were identified in the *Arabidopsis* CAD family using Pfam (http://pfam.sanger.ac.uk/search) databases, and these domains were used as the target sequence to screen *PbCAD* candidate genes using the same method. We removed repetitive sequences from the screening results. The MW and pI of the amino acid sequence of family members were analysed using the online ExPASy-Compute pI/Mw tool (http://web.expasy.org/compute_pi/). The chromosomal location information for each family member was directly obtained from the pear genome database.

### Phylogenetic analysis and amino acid sequence alignment

Phylogenetic trees were constructed using the N-J method (bootstrap=1000) in MEGA5.1 software (Tamura et al., 2011). The amino acid sequence alignment was performed using BioEdit software (www.mbio.ncsu.edu/bioedit/bioedit.html). Sequence similarity and identity analyses were conducted using Iden and Sim in Sequence Manipulation Suite (https://sites.ualberta.ca/∼stothard/javascript/ident_sim.html; Stothard, 2000). The GenBank accession numbers and amino acid sequences used for constructing the phylogenetic tree and sequence alignment are listed in Tables S10 and S11.

### Conserved motifs and gene structure prediction

Conserved motifs were confirmed using Multiple Em for Motif Elicitation (MEME) (http://meme-suite.org/; Bailey et al., 2006). The parameters were set as follows: an optimum motif width of no less than 6 and no greater than 200 and a maximum number of motifs of 20. Exon-intron structures were generated using GSDS (http://gsds.cbi.pku.edu.cn/).

### Chromosomal locations and gene duplications

The location information of each *CCR* or *CAD* was obtained from the pear genome database and mapped onto the diagram using MapInspect software (http://mapinspect.software.informer.com/).

We decided that gene duplication occurred if the length of the matching portion of the two gene sequences was greater than 80% of the longer one and the similarity of the two gene sequences was greater than 80%. If the two genes are located on the same chromosome and the number of genes between the two given genes is less than 5, they are considered tandem duplicates; otherwise, they are segmental duplicates (Ouyang et al., 2009; Kong et al., 2013; Wei et al., 2013). Ka and Ks were calculated using DnaSP v5.0 software (Rozas and Rozas, 1995). Sliding window analysis was also performed using this software.

### Cis-regulatory element analysis and three-dimensional structure prediction

We analysed the 2000-bp sequence upstream from each gene's initiation codon (ATG) in the pear genome and evaluated the type and number of cis-acting regulatory DNA elements (cis-elements) using the PLANT CARE program (http://bioinformatics.psb.ugent.be/webtools/plantcare/html/; Lescot et al. 2002).

The Protein Fold Recognition server (www.sbg.bio.ic.ac.uk/~phyre2/html/page.cgi?id=index) was used to predict the three-dimensional structures of PbCCRs or PbCADs.

### RNA extraction and reverse transcription

To examine expression of *PbCCR* and *PbCAD* genes, buds, stems, leaves and fruit were obtained from 50-year-old pear trees grown on a farm in Dangshan, Anhui, China. We collected fruit at 8 time points: 15 DAF, 23 DAF, 39 DAF, 47 DAF, 63 DAF, 79 DAF, 102 DAF and 145 DAF. Sixty pear fruit were collected at each time point, and the samples were stored at −80°C until use.

Total RNA was isolated from each sample using a total RNA isolation kit (Tiangen, China). Reverse transcription was performed using a PrimeScript RT Reagent Kit with gDNA Eraser (Perfect Real Time, Takara, China).

### qRT-PCR analysis for *PbCCR*s/*CAD*s

qRT-PCR was performed using CFX96 Touch™ Real-Time PCR Detection System (Singapore) to examine gene expression in cDNA samples for 8 developmental stages of pear fruit (15 DAF, 39 DAF, 47 DAF, 55 DAF, 63 DAF, 79 DAF, 102 DAF and 145 DAF) and in buds (B), stems (S) and leaves (L). Each reaction was performed in triplicate. *Tubulin* (GenBank accession no. AB239680.1) (Wu et al., 2012) was used as an internal reference, and the relative expression levels of genes were calculated using the 2^−ΔΔCT^ method (Livak and Schmittgen, 2001). Primers were designed using Primer Premier 5.0, and the primer sequences are listed in Table S12. The reactions contained the following: 10 μl SYBR Premix Ex Taq II (2x), 2 μl template cDNA, 0.8 μl forward and reverse primers, and ddH_2_O to 20 μl. The qRT-PCR reaction was carried out under the following conditions: 95°C for 3 min, followed by 40 cycles of 95°C for 10 s, 52°C for 15 s, and 72°C for 30 s.

### Measurement of lignin content

The lignin content of pear fruit was measured using the Cai et al. (2010) method. After peeling, the fruit was ground into a uniform powder and passed through a mesh sieve. The powder was extracted with methanol and oven dried. A small amount (0.2 g) of this powder was extracted with 15 ml 72% H_2_SO_4_ at 30°C for 1 h, combined with 115 ml distilled water and boiled for 1 h. The volume was kept constant during boiling. The liquid mixture was filtered, and the residue was rinsed with 500 ml hot water until the rinse solution was neutral. The lignin was then dried, after which it was weighed three times.

### Staining of stone cells in pear fruit

Cross-sections of pear fruit were stained with 1.0% phloroglucinol and 1.0 mol/l HCl as described in the Wiesner lignin staining method (Thévenin et al., 2011; Pradhan and Loqué, 2014). We photographed the sections after chromogenesis to observe the stone cell mass distribution.

### Subcellular localization analysis of candidate PbCCRs and PbCADs

Primers containing enzyme cleavage sites (Table S12) were designed to amplify the coding sequence (CDS) of candidate genes (*PbCAD2*, *-3* and *PbCCR1*, *-2*, *-3*) according to the multiple cloning site of binary vector pCAMBIA1304 (GenBank accession no. AF234300.1). We constructed the recombinant eukaryotic expression plasmids pCAMBIA1304-*PbCCR*/*CAD*-*GFP*, which were introduced into *Agrobacterium* EHA105 via electroporation. Positive monoclonal cells were selected and cultured in LB liquid medium with 50 mg/l Kan and 50 mg/l Rif and shaken overnight at 28°C and 180 rpm. We expanded the culture and collected the bacteria after PCR confirmation; the bacteria were then resuspended in infection solution [10 mM 2-(N-Morpholino) ethanesulfonic acid, 10 mM MgCl_2_, 0.1 mM acetosyringone]. The bacterial suspension was aged in the dark for 3 h after dilution to an OD_600_ of approximately 0.8. The lower epidermis of *Nicotiana benthamiana* leaves was removed using a sterile syringe needle. After removing the needle, with the fingers against the back of the wound, the infection fluid was injected into the wound until it fully penetrated the leaves; the plants were co-cultured in darkness for 3 days. *Agrobacterium* containing the empty plasmid was used as the control. GFP fluorescence was observed using a confocal laser microscope (OLYMPUS, Japan) after preparation of microscope slides of tobacco leaves at 3 days post-infection.

## Supplementary Material

Supplementary information
