# Correction: Characterization and analysis of *CCR* and *CAD* gene families at the whole-genome level for lignin synthesis of stone cells in pear (*Pyrus bretschneideri*) fruit

**DOI:** 10.1242/bio.052985

**Published:** 2020-05-21

**Authors:** Xi Cheng, Manli Li, Dahui Li, Jinyun Zhang, Qing Jin, Lingling Sheng, Yongping Cai, Yi Lin

There were errors in Biology Open (2017) 6, bio026997 (doi:10.1242/bio.026997).

The wrong Fig. 8 was used in the published paper. The correct and original figure are shown below and both the online full-text and PDF versions of the article have been updated.

**Fig. 8 (correct image).**

**Figure:**
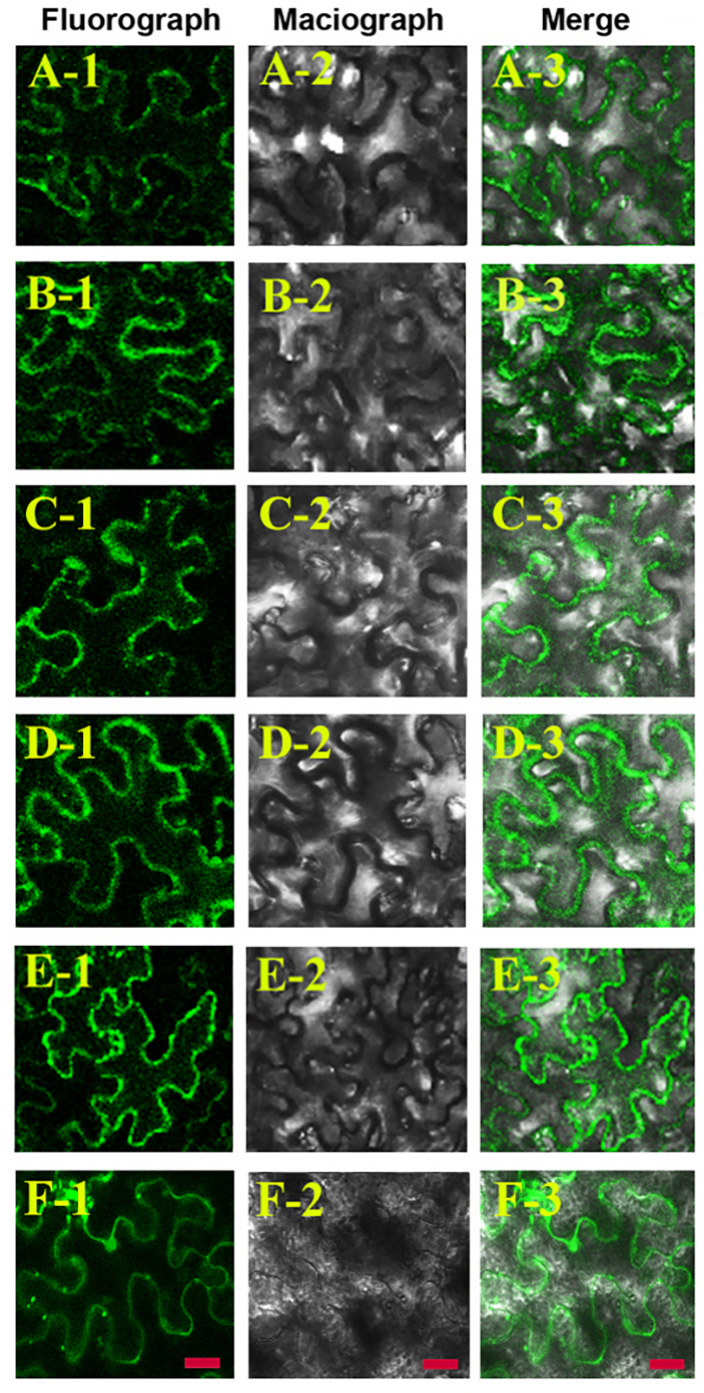

**Fig. 8 (original image).**

**Figure:**
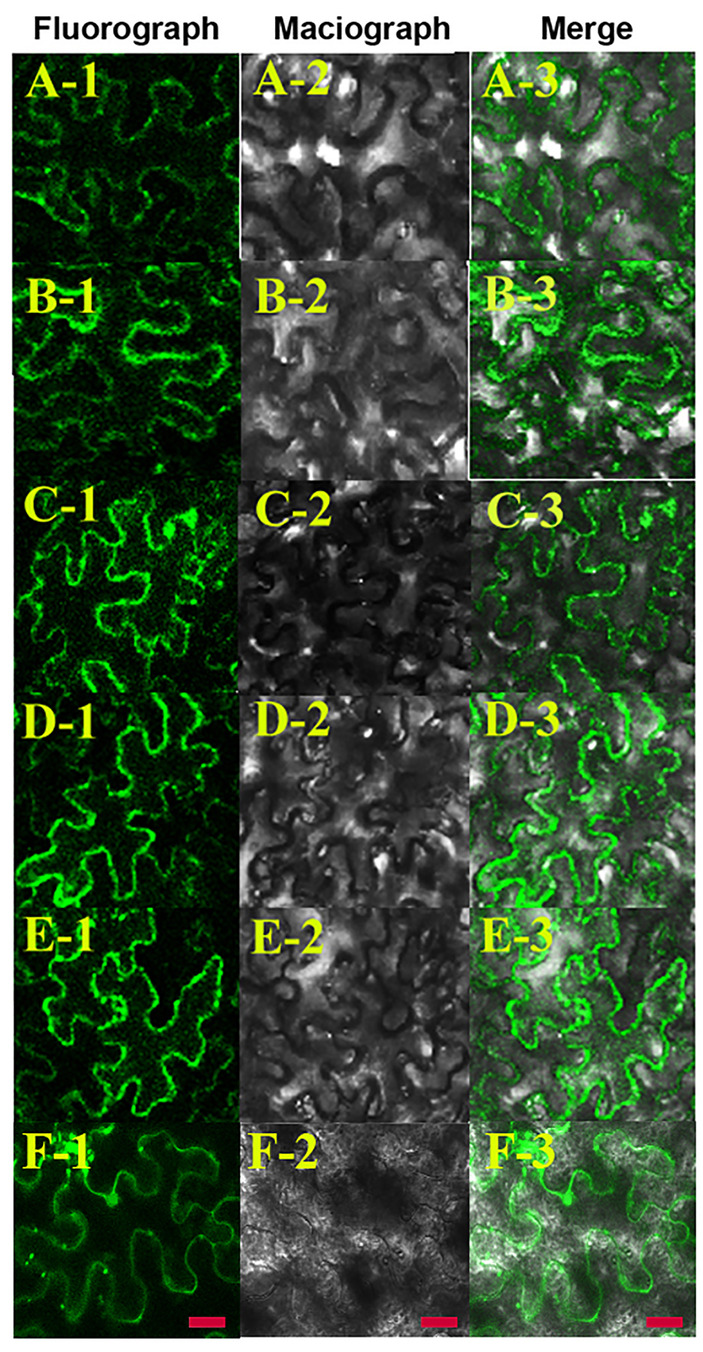

The authors apologise for these errors and any inconvenience they may have caused.

This correction does not affect the results in the article or the conclusions of this study.

