# Supplementary material for: Characterization and analysis of CCR and CAD gene families at the whole-genome level for lignin synthesis of stone cells in pear (Pyrus bretschneideri) fruit
Source: Biol Open. 2017 Nov 15;6(11):1602–13. doi: 10.1242/bio.026997 (PMC5703608; doi:10.1242/bio.026997)
Supplement: Supplementary information [file biolopen-6-026997-s1.pdf]

Supplementary Material

Table S1 Sequence characteristics of 31 *CCR*/*CCR-like* genes identified in the pear genome.

Gene name	Gene code	Chromosome	Molecular weight (kDa)	pI	ORF (bp)
PbCCR1	Pbr022402.1	Chr17	37.2	6.51	1017
PbCCR2	Pbr022405.1	Chr17	37.3	6.16	1023
PbCCR3	Pbr022403.1	Chr17	37.2	6.32	1017
PbCCR-like1	Pbr017203.1	Chr3	35.4	6.12	975
PbCCR-like2	Pbr022903.1	Chr2	34.9	5.80	960
PbCCR-like3	Pbr015109.1	Chr6	34.9	6.27	939
PbCCR-like4	Pbr022905.1	Chr2	34.5	6.02	951
PbCCR-like5	Pbr009662.1	Chr7	35.2	6.95	963
PbCCR-like6	Pbr022902.1	Chr2	110.4	5.74	3048
PbCCR-like7	Pbr011590.1	Chr11	35.5	5.67	972
PbCCR-like8	Pbr009660.1	Chr7	33.0	5.35	900
PbCCR-like9	Pbr011595.1	Chr11	30.5	5.93	831
PbCCR-like10	Pbr028835.1	Chr13	35.0	5.78	951
PbCCR-like11	Pbr011592.1	Chr11	32.5	6.31	888
PbCCR-like12	Pbr026638.1	Chr8	36.2	5.89	999
PbCCR-like13	Pbr028834.2	Chr13	30.0	6.17	813
PbCCR-like14	Pbr015104.1	Chr6	36.7	6.05	996
PbCCR-like15	Pbr022901.1	Chr2	33.1	5.65	903
PbCCR-like16	Pbr007971.1	/	36.5	8.08	990
PbCCR-like17	Pbr015106.1	Chr6	38.5	7.06	1026
PbCCR-like18	Pbr007973.1	/	34.3	6.67	918
PbCCR-like19	Pbr022904.1	Chr2	63.7	8.13	1728
PbCCR-like20	Pbr042690.1	Chr7	19.5	4.93	528
PbCCR-like21	Pbr013866.1	Chr6	33.4	5.22	897
PbCCR-like22	Pbr013435.1	Chr1	37.7	5.38	1011
PbCCR-like23	Pbr027042.1	Chr17	39.8	5.82	1083
PbCCR-like24	Pbr031402.1	Chr16	34.4	5.29	918
PbCCR-like25	Pbr024414.1	Chr9	40.0	6.28	1080
PbCCR-like26	Pbr030621.1	Chr9	38.7	6.68	1050
PbCCR-like27	Pbr039962.1	Chr7	34.3	5.42	912
PbCCR-like28	Pbr007658.1	/	23.2	5.43	615

Note: "/" indicates that the specific chromosome is not known.

Biology Open • Supplementary information

Table S2 Sequence characteristics of 26 *CAD* genes identified in the pear genome.

Gene name	Gene code	Chromosome	Molecular weight (kDa)	pI	ORF (bp)
PbCAD1	Pbr040236.1	Chr17	37.5	7.97	1053
PbCAD2	Pbr026287.1	Chr10	38.4	5.76	1068
PbCAD3	Pbr026289.1	Chr10	38.5	5.83	1065
PbCAD4	Pbr003622.1	Chr13	23.5	6.16	660
PbCAD5	Pbr024188.1	Chr10	35.6	6.41	969
PbCAD6	Pbr003623.1	Chr13	39.8	6.26	1113
PbCAD7	Pbr040238.1	Chr17	49.1	7.89	1374
PbCAD8	Pbr003619.1	Chr13	22.7	5.42	639
PbCAD9	Pbr040250.1	Chr17	39.0	6.02	1074
PbCAD10	Pbr014932.1	Chr10	35.3	6.60	954
PbCAD11	Pbr003617.1	Chr13	38.8	6.86	1086
PbCAD12	Pbr025679.1	Chr5	38.7	7.20	1077
PbCAD13	Pbr006899.1	/	39.0	6.89	1074
PbCAD14	Pbr005802.1	Chr15	39.9	7.62	1098
PbCAD15	Pbr003627.1	Chr13	39.3	6.00	1089
PbCAD16	Pbr003626.1	Chr13	40.4	5.33	1116
PbCAD17	Pbr004676.1	Chr15	39.2	6.89	1083
PbCAD18	Pbr004677.1	Chr15	38.8	8.00	1083
PbCAD19	Pbr010181.1	Chr14	38.2	6.82	1065
PbCAD20	Pbr040249.1	Chr17	38.6	6.41	1071
PbCAD21	Pbr004679.1	Chr15	32.4	6.05	897
PbCAD22	Pbr014931.1	Chr10	33.1	7.17	918
PbCAD23	Pbr005801.1	Chr15	22.3	8.77	600
PbCAD24	Pbr024187.1	Chr10	51.2	8.26	1428
PbCAD25	Pbr015311.1	Chr15	27.5	5.36	759
PbCAD26	Pbr004675.1	Chr15	23.3	8.47	657

Note: “/” indicates that the specific chromosome is not known.

Table S3 Motif composition of CCR proteins from pear.

Motif No.	Width	Best match
Motif 1	41	WYCLSKTLAEKAAWKFAKENGIDMVTINPGLVMGPLLQPTN
Motif 2	29	CVTGASGFIGSWLVKLLLQRGYTVHATVR
Motif 3	33	ERLHLFKADLLDYNSFDAAVNGCQGVFHTASPC
Motif 4	21	VHVRDVAEAHIQAFENPSASG
Motif 5	19	MIEPAVKGTLNVLQACAQA
Motif 6	21	PPDQVIDETWWSDPEYCKQTK
Motif 7	29	EKAQRLGWTFRPLEETLRDTIESLKEKGF
Motif 8	15	VKRVVFTSSMAAVIW
Motif 9	15	RYCCFNQVIHWGDFV
Motif 10	15	NDDGKTEHLWELDGA
Motif 11	21	EILRKLYPTYPYPEKCNDNQM
Motif 12	15	YLKGCTQMYQNGFMG
Motif 13	21	DPFDYHSIMDAFKGCSGVFHT
Motif 14	14	NISNEMLNNLMNGF
Motif 15	8	SVTDPQAE
Motif 16	11	FEPPQDQPDYD
Motif 17	15	LPVPTKQEQDSIKIQ
Motif 18	11	FESIPCNYWTF
Motif 19	15	MPADSSSVSGHGQTI
Motif 20	8	TPFINYTF

Biology Open • Supplementary information

Table S4 Motif composition of CAD proteins from pear.

Motif No.	Width	Best match
Motif 1	40	ETQEMIDFCAKHNITADIEVIPMDYVNTAMERLAKNDVRY
Motif 2	40	GGLGHMAVKFAKAMGHKVTVISTSPNKKEEAIEHLGADSF
Motif 3	40	MVANERFCVRIPENLPLDQAAPLLCAGITTYSPMRYFGLN
Motif 4	40	KVLYCGICHSDLHWAKNEWGMSNYPMVPGHEIVGVVTEVG
Motif 5	33	QKFKVGDHVGVGCMVGSCRSCEYCNQNLENYCP
Motif 6	40	SRDQEQMQAAMGTLDFIIDTVSADHPLLPWIGLLKTHGKL
Motif 7	29	QEHPQKCFGWAARDSSGVLSPFKFSRRNT
Motif 8	21	IWTYNSHYWDGTTTYGGYSDI
Motif 9	21	PVFPLIMGRKIIAGSFIGGMK
Motif 10	11	RFVIDIANTLK
Motif 11	11	QPGKHLGVVGL
Motif 12	11	MVGAPEQPLEL

Table S5 Ka/Ks analysis for *PbCCR*/*CAD* duplicated genes of pear.

Duplicated gene pairs		Ka	Ks	Ka/Ks	Purifying selection	Duplicated type
PbCCR10	***PbCCR13***	**0.0115**	**0.0356**	**0.32**	**Yes**	**Tandem**
PbCCR23	***PbCCR25***	**0.0742**	**0.3002**	**0.25**	**Yes**	**Segmental**
PbCCR22	***PbCCR27***	**0.0675**	**0.2145**	**0.31**	**Yes**	**Segmental**
PbCCR8	***PbCCR15***	**0.0916**	**0.1369**	**0.67**	**Yes**	**Segmental**
PbCCR21	***PbCCR24***	**0.0608**	**0.2660**	**0.23**	**Yes**	**Segmental**
PbCAD2	***PbCAD3***	**0.0744**	**0.4008**	**0.19**	**Yes**	**Tandem**
PbCAD5	***PbCAD10***	**0.0123**	**0.0281**	**0.44**	**Yes**	**Tandem**

Biology Open • Supplementary information

Table S6 Putative cis-acting regulatory elements in *PbCCR* promoters.

Cis-element	AC	HSE	ERE	TCA-element	TGACG-motif	Box-W1	Spl	GARE-motif	ABRE	LTR
Function	MYB binding	heat stress	ethylene	SA	MeJA	fungal elicitor	light	GA	ABA	low-temperature
PbCCR1	**3**	**2**		**1**	**2**	**1**	**1**		**1**	**1**
PbCCR2	**2**	**4**			**1**		**2**	**2**	**2**	
PbCCR3				**2**	**1**		**2**		**2**	**1**
PbCCR-like1		**2**	**2**	**1**		**1**	**1**	**1**	**1**	**1**
PbCCR-like2		**3**			**3**		**1**		**2**	**1**
PbCCR-like3	**1**				**3**	**2**	**6**			**1**
PbCCR-like4		**2**		**3**		**1**	**7**		**7**	**3**
PbCCR-like5		**1**	**1**		**2**	**2**	**1**		**2**	**1**
PbCCR-like6	**1**			**2**	**1**		**1**	**2**	**5**	**1**
PbCCR-like7		**2**	**1**		**1**	**2**	**4**	**2**	**1**	**1**
PbCCR-like8		**4**		**2**	**1**		**1**			
PbCCR-like9			**2**	**2**		**1**	**4**	**2**		
PbCCR-like10		**3**			**2**	**1**	**3**		**3**	
PbCCR-like11	**3**	**1**		**2**	**1**		**3**	**2**		**3**
PbCCR-like12		**1**		**1**	**1**				**2**	**1**
PbCCR-like13		**1**		**1**	**1**	**2**	**3**	**1**	**4**	**1**
PbCCR-like14		**3**			**4**		**3**			
PbCCR-like15		**1**		**1**		**1**	**4**	**1**	**3**	
PbCCR-like16					**5**	**1**	**3**		**3**	**1**
PbCCR-like17	**1**				**1**	**1**	**1**	**2**	**1**	**1**
PbCCR-like18										
PbCCR-like19					**3**		**1**	**2**	**1**	
PbCCR-like20		**1**	**1**		**3**	**1**		**1**	**1**	**1**
PbCCR-like21	**1**	**3**	**1**	**2**	**4**	**1**	**4**		**5**	
PbCCR-like22	**2**				**4**		**5**	**1**	**2**	**1**
PbCCR-like23		**4**		**3**			**19**		**2**	
PbCCR-like24		**3**		**1**		**1**	**2**	**2**		
PbCCR-like25	**1**	**2**			**1**		**1**	**1**		
PbCCR-like26		**3**	**1**	**2**	**5**		**1**	**1**		
PbCCR-like27	**2**		**1**	**2**		**2**	**3**	**1**	**3**	
PbCCR-like28										

Table S7 Putative cis-acting regulatory elements in *PbCAD* promoters.

Cis-element	AC	HSE	ERE	TCA-element	TGACG-motif	Box-W1	Spl	RY-element	ABRE	LTR
Function	MYB binding	heat stress	ethylene	SA	MeJA	fungal elicitor	light	seed-specific	ABA	low-temperature
PbCAD1		**6**	**2**	**3**	**6**	**2**	**1**		**2**	**1**
PbCAD2	**2**	**1**		**2**	**1**	**1**	**29**			
PbCAD3	**1**	**2**		**1**	**2**	**1**				
PbCAD4	**1**				**2**		**2**		**3**	
PbCAD5				**1**	**4**	**3**	**5**		**3**	**2**
PbCAD6				**1**	**4**		**1**			
PbCAD7		**6**	**3**	**1**	**3**	**3**	**1**		**4**	
PbCAD8	**1**	**1**	**1**	**2**	**4**					**1**
PbCAD9		**1**		**1**	**1**	**1**				
PbCAD10				**1**	**4**	**3**	**5**		**3**	**2**
PbCAD11		**2**	**1**	**1**	**1**	**2**			**3**	**2**
PbCAD12		**3**		**1**	**4**				**4**	**2**
PbCAD13				**1**	**3**	**1**	**5**		**5**	
PbCAD14	**1**	**1**		**1**	**2**		**2**		**1**	**1**
PbCAD15			**2**	**3**	**3**		**1**			**1**
PbCAD16		**3**	**1**		**2**				**1**	
PbCAD17	**1**	**1**	**1**	**4**	**4**	**1**	**2**			
PbCAD18		**4**			**3**	**2**	**1**		**4**	
PbCAD19		**4**			**3**				**2**	
PbCAD20		**4**	**4**	**1**	**1**	**2**	**1**		**5**	**1**
PbCAD21		**2**		**4**	**1**	**2**	**2**			
PbCAD22		**1**		**3**	**2**	**1**	**1**		**1**	**2**
PbCAD23		**3**	**1**	**1**	**2**	**1**	**1**		**6**	
PbCAD24		**1**		**3**	**2**	**1**	**1**			**2**
PbCAD25					**4**		**1**	**1**	**1**	
PbCAD26		**1**			**1**		**2**		**3**	

Biology Open • Supplementary information

Table S8 Comparison of predictive residues involved in CCR enzymatic activity. AtCCR1 (At1g15950); AtCCR2 (At1g80820); PhCCR1 (AHX56186); TaCCR1 (ABE01883).

Position	AtCCR1	AtCCR2	PbCCR1	PbCCR2	PbCCR3	PhCCR1	TaCCR1	Residue function
**17**	F	F	F	F	F	Y	Y	Substrate Binding
**123**	S	S	S	S	S	S	S	Substrate Binding
**124**	I	I	I	I	I	I	I	Substrate Binding
**125**	G	G	G	G	G	G	G	Substrate Binding
**157**	Y	Y	Y	Y	Y	Y	Y	Substrate Binding
**158**	C	C	C	C	C	C	C	Substrate Binding
**161**	K	K	K	K	K	K	KV	Substrate Binding
**185**	V	V	V	V	V	V	V	Substrate Binding
**199**	S	S	S	S	S	S	S	NADPH Binding
**202**	H	H	H	H	H	H	H	Substrate Binding
**205**	K	K	K	K	K	K	K	Substrate Binding
**220**	A	V	A	A	G	A	A	Substrate Binding
**253**	R	R	R	R	R	R	R	Substrate Binding
**284**	Y	Y	Y	Y	Y	Y	Y	Substrate Binding

Table S9 Comparison of predictive residues involved in CAD/SAD enzymatic activity. AtCAD4 (AY302081); AtCAD5 (AY302082); OsCAD2 (Os02g09490); PtSAD (AF273256).

Position	AtCAD4	AtCAD5	PbCAD2	PbCAD3	PbCAD25	OsCAD2	PtSAD	Residue function
**49**	T	T	S	T	S	T	S	$NADP^+$ Binding / H^+ shuttle
**52**	H	H	H	H	H	H	H	$NADP^+$ Binding
**53**	Q	Q	Q	Q	Q	Q	S	Substrate Binding
**57**	D	D	D	D	E	H	D	H^+ shuttle
**58**	L	L	L	L	I	L	W	Substrate Binding
**60**	M	M	M	M	S	A	F	Substrate Binding
**70**	E	E	E	E	/	E	E	Zn^{2+} Binding
**95**	V	C	L	L	/	V	C	Substrate Binding
**119**	W	W	W	W	/	W	L	Substrate Binding
**192**	V	V	V	V	V	V	L	$NADP^+$ Binding
**211**	S	S	S	S	S	S	S	$NADP^+$ Binding
**212**	S	S	S	S	S	S	T	$NADP^+$ Binding
**213**	S	S	S	S	S	S	S	$NADP^+$ Binding
**216**	K	K	K	K	K	K	K	$NADP^+$ Binding
**276**	V	V	V	A	A	V	A	Substrate Binding
**286**	P	P	P	P	V	P	F	Substrate Binding
**289**	I	M	M	M	I	M	I	Substrate Binding
**290**	L	L	L	L	L	L	A	Substrate Binding
**299**	F	F	F	F	F	F	G	Substrate Binding
**300**	I	I	V	V	I	I	I	Substrate Binding

Biology Open • Supplementary information

Table S10 GenBank accession codes used for constructing phylogenetic trees.

Gene Name	GenBank No.	Species	Gene Name	GenBank No.	Species
ZmCAD1	AJ005702	*Zea mays*	***GmCAD1***	HQ651807	*Glycine max*
TaCAD1	GU563724	*Triticum aestivum*	***MtCAD1***	KEH44099	*Medicago truncatula*
AtCAD1	AY288079	*Arabidopis thaliana*	***NtCAD***	CAA44217	*Nicotiana tabacum*
AtCAD2	AY302077	*Arabidopis thaliana*	***PviCAD***	GU045611	*Panicum virgatum*
AtCAD3	AY302078	*Arabidopis thaliana*	***Bmr6***	Sb04g005950	*Sorghum bicolor*
AtCAD4	AY302081	*Arabidopis thaliana*	***PrCCR***	AFC38436.1	*Pinus radiata*
AtCAD5	AY302082	*Arabidopis thaliana*	***PaCCR***	CAK18610.1	*Picea abies*
AtCAD6	AY302075	*Arabidopis thaliana*	***AtCCR1***	At1g15950	*Arabidopis thaliana*
AtCAD7	AY302079	*Arabidopis thaliana*	***AtCCR2***	At1g80820	*Arabidopis thaliana*
AtCAD8	AY302080	*Arabidopis thaliana*	***AtCCR-like1***	At1g76470	*Arabidopis thaliana*
AtCAD9	AY302076	*Arabidopis thaliana*	***AtCCR-like2***	At2g02400	*Arabidopis thaliana*
OsCAD1	Os10g11810	*Oryza sativa*	***AtCCR-like3***	At2g33590	*Arabidopis thaliana*
OsCAD2	Os02g09490	*Oryza sativa*	***AtCCR-like4***	At2g33600	*Arabidopis thaliana*
OsCAD3	Os10g29470	*Oryza sativa*	***AtCCR-like5***	At5g58490	*Arabidopis thaliana*
OsCAD4	Os11g40690	*Oryza sativa*	***HvCCR1***	BAK07589	*Hordeum vulgare*
OsCAD5	Os08g16910	*Oryza sativa*	***HvCCR2***	BAJ97892	*Hordeum vulgare*
OsCAD6	Os04g15920	*Oryza sativa*	***HvCCR3***	BAK01264	*Hordeum vulgare*
OsCAD7	Os04g52280	*Oryza sativa*	***HvCCR4***	BAJ89483	*Hordeum vulgare*
OsCAD8	Os09g23530	*Oryza sativa*	***OsCCR1***	Os09g25150	*Oryza sativa*
OsCAD9	Os09g23540	*Oryza sativa*	***OsCCR2***	Os08g34280	*Oryza sativa*
OsCAD10	Os09g23550	*Oryza sativa*	***OsCCR3***	Os08g17500	*Oryza sativa*
OsCAD11	Os09g23560	*Oryza sativa*	***OsCCR4***	Os09g04050	*Oryza sativa*
OsCAD12	Os03g12270	*Oryza sativa*	***OsCCR5***	Os02g08420	*Oryza sativa*
PtoCAD1	KF145200	*Populus tomentosa*	***OsCCR6a***	Os02g56680	*Oryza sativa*
PtoCAD2	KF145201	*Populus tomentosa*	***OsCCR6b***	Os02g56690	*Oryza sativa*
PtoCAD3	KF145202	*Populus tomentosa*	***OsCCR6c***	Os02g56700	*Oryza sativa*
PtoCAD5	KF145203	*Populus tomentosa*	***OsCCR6d***	Os02g56720	*Oryza sativa*
PtoCAD6	KF145199	*Populus tomentosa*	***OsCCR7***	Os02g56460	*Oryza sativa*
PtoCAD7	KJ159966	*Populus tomentosa*	***TaCCR1***	ABE01883.1	*Triticum aestivum*
PtoCAD8	KF145195	*Populus tomentosa*	***TaCCR2***	AAX08107.1	*Triticum aestivum*
PtoCAD9	KF145196	*Populus tomentosa*	***ZmCCR1***	NP001105488.1	*Zea mays*
PtoCAD12	KJ159967	*Populus tomentosa*	***PtCCR***	AAF43141.1	*Populus tremuloides*
PtSAD	AF273256	*Populus tremuloides*	***EgCCR***	CAA56103.1	*Eucalyptus gunnii*
BdCAD5	XP_003570974.1	*Brachypodium distachyon*	***LlCCR1***	ABL01801.3	*Leucaena leucocephala*
CsCAD3	HQ880208	*Camellia sinensis*	***PhCCR1***	AHX56186.1	*Petunia x hybrida*
ShCAD1	L36823	*Stylosanthes humilis*	***PtrCCR2***	ACC63879.1	*Populus trichocarpa*
ShCAD3	L36456	*Stylosanthes humilis*	***ZmCCR2***	NP001105715.1	*Zea mays*

Biology Open • Supplementary information

Table S11 Amino acid sequences of CCRs and CADs used in this study.

>PbCCR1

MPAVSSSVSGHGQTICVTGAGGFIASWMVKLLLERGYTVRGTLRNPDDPKNAHLRELEGAAERLTLCRADLLYESL
KEAINGCDGVFHTASPVTDDPEQMVEPAVNGTKNVIQAAAEAKVRRVVFTSSIGAVYMDPTRGPDVVVDEACWSDL
EFCKNTKNWYCYGKAVAEQAAWAEAKEKGVDLVVVNPVLVLGPLLQPTVNASIIHILKYLTGSTQTYANSVQAYVH
VRDVALAHILVYETPTASGRYLCAESVLHRGDVVEILAKFFPEYPIPNKCKDNGKPRAKPYKFTNQKLRDLGLEFT
PVKHTLYETVKSLQEKGHLPVPTKQEQDSIKIQS

>PbCCR2

MPADSSSVSGHGQTICVTGAGGFIASWMVKLLLESGYTVRGTLRNPHDPKNAYLSELEGAAARLTLCRADLLDYES
LKEAINGCDGVFHTASPVTDDPEQMVEPAVNGTKNVIQAAAEAKVKRVVFTSSIGAVYMDPTRGPDVVVESCWSDL
EFCKNTKNWYCYGKAVAEQAAWAEAKEKGVDLVVVNPVLVLGPLLQPTVNASIFHILKYLTGSTQTYANSVQAYVH
VRDVALAHILVYETPSASGRYICAESVLHRGDVVEILAKFFPEYPIPNKCKDNGKPRAESYKFTNQKLRDLGLEFT
PVKHTLYETVKSLQEKGHLPVPTKQEQDSIKIRSCL

>PbCCR3

MPADSSSVSGHDQTICVTGAGGFIASWMVKLLLERGYTVRGTLRNPHDPKNAHLRELEGAAERLTLCRADLLDYES
LKEAINGCDGVFHAASPITDDPEQMVEPAVNGTKNVIQVAGEAKVKRVVFTSSIGAVYMDPTRGPDVVVDESCWSD
LEFCKNTKNWYCYGKAVAEQVAWVEAKEKGVDLVVVNPVLVLGPLLQSTVNASTIHILKYLTGSTQTYANSIQGYV
HVRDVALAHILVYETPSASGRYICAKSVLHRGDVVEILAKFFPKYPIPNKCKDNGKSRAEPYKFTNQKLLDLGLEF
TPVKDTLYETVKSLQEKGHLPVPPKQEQDSIKIQS

>PbCCR-like1

MSSGVGKVVCVTGASGYIASWLVKLLLQRGYTVKASIRDPNDPTKTEHLHALDGAQDRLQLFKANLLEEGSFDSAV
EGCEGVFHTASPFYHDVTDPKAELLEPAVKGTLNVLNSCAKSPSIKRVVLTSSIAAVAYNGKPRTPDVVIDETWFT
DPDVCKESKLWYVLSKTLAEDAAWKFVKEKGIDLVTINPAMVIGPLLQPTLNTSAAAVLNVIKGARTFPNASFGWI
NVKDVANAHIQAFESPTAGGRYCLVETVAHFSEVVRILRELYPTLQLPEKCADDKPFVPTYQVSKEKAKSLG
VEFIPLDVSLKETVESLKEKGFVSF

>PbCCR-like2

MSAVEGKTVCVTGASGYIASWLVKLLLQKGYTVKGTVRDPNDSKKTEHLRSLDGAKERFHLFKADLLEEGSFDAVV
DGCQGVFHTASPVLLSVTDPQGELLDPAVKGTLNVLQSCAKFPSIKRVVLTSSIASVMMTGVPLTSDAVLDETWYS
DPLFCEKHKQWYLLSKTLAEETAWKFAKGNGIDLVSLNPGLTIGPLLQPTLNFSVELLQNFMSGTQTTISNPFVDV
RDVASAHIQAFEIPSASGRYCLVGQVADDLDILKILRQLYPTLTLAEVVNPSDSKYQVSKEKAKGLGITFLPLETS
LRDTIESLKEKGFLKI

>PbCCR-like3

MAVEKGRYCVTGAGGFLASWVVKLLLSKDSIVHGTLRDPSNDRHAHLKKLDKASENLKLFKADLLDYESLRTAIQG
CDGVFHVASPVPYDPVELIAPAVKGTLNVLKACLEAKVKRVVFVSSAASLVMNPKWSQGQVLDETCWSDKDYCRST
KNWYCLSKTEAEYEALEYARINGLDLVTVCPTLIMGPILQSTVNASTLVLIKLLKEGYESLENNPRMIVDVRDVAE
AVVMVYEKPEAEGRYICTAHNIKTTALVEELKSIYPNYSYPKNFVEVEERPRLSSEKLQRLGLSFRPLKETLTDSV
ESYKEAGLL

>PbCCR-like4

MSDTRATKVVSVTGASGYIASWLVKLLLQRGYTVKASVRDPNDKKKTAHLLALDGAKERLQLFKADLLEEGSFDSL
VEGSEGVFHTASPFYHNVSDPQAELIDPALKGTLNVLRSCAKVPSIKRVVITSSMAAVAFNGKPLAPDVVVDESWF
SDVAVCEKSKTLAEEAAWKFAKENGIDIATINPGLVTGPLLQPTLNTSVEPVLKLVNGAERFPNTTYRWVDVRDVA
TAHILALENASASGRYCLVGRVIHSSDTVNILRDLFPALKLPEECADDKPFTPTYQVSNERAQSLGVKFTPLEVTL
KDTVESLKEKNFF

>PbCCR-like5

MSTGESKTVCVTGASGYIASWLVKLLLQKGYTVKATVRNPNDAKKKEHLLSLDGAKERLHVFKADLLEERSFDAVI
DGCVGVFHTASPAQFSATDPQVEIIEPAVKGTLNVLKSCAKFPAVKRVVITSSLASVRLSGKPLTSDVVMDETWYS
DPLFCKEIKQWYPLSKTLAEEAAWKFAKGNGIDLVTIHPGFVIGPLLQPSLNLSVEFLLNLMSGMETPFINYAFVD
VRDVAFAHIQAFEVPSASGRYCLVAQVADGPDTLKILRELYPTLSLCEPGNPSGSKFQVSREKAKCLGITFLPLET
SLKDTVESLMEKCFLKF
>PbCCR-like6
MSGGTKVVCVTGASGYIASWLVKLLLQRGYTVKATVRDPSDPKKSEHLLSLDGAKERLHLFRADLLEEGSFDTVVD
GCQGVFHTASPVPFSVTDPQAELVEPAVKGTLNVLKSCVKFPTVKRVVLTSSMASVLVNGKPLTSDVVLDETWYSD
PLVCEKNKRWYALSKTLAEEAACKYAKENGIDLVTMNPGFVIGPLLQPTLNVSVEMIQNLITGKVTKTLPLSNYRS
IDVRDVVSAHILAFEVPSASGRYCLVSNVTHASEVLKIIQDLYPTLHLPEKCEIGGTSDPKYPVSAEKAKGDPKKS
EHLLSLDGARERLHLFRADLLEEGSFDTVVDGCQGVFHTASPVSFSVTDPQAELVEPAVKGTLNVLKSCVKIPTVK
RVVLTSSMASVSVNGKPLTSDVVLDETWYSDPLVCEKNERWYALSKTLAEEAACKYAKENGIDLVTMNPGFVIGPL
LQPTLNLSVEMIQNLITGTGTKTLPISNYRSIDVRDVVSAHILAFEVPSASGRYCLVSNVTHASEVLKIIQDLYPT
LHLPEECEIGTTSDPKYPVSAEKAKGLGINFLPLEWGGCNPSSTHQFCLCNATDDPKKTGHLLSLDGAKERLHLFK
TGLLEEGAFDAVIDGCVCVFHTASPVQLSATDPQAEIIEPAVKGTLNVLKSCVKFPTVKRVVLTSSMSSVSHSRTP
ITSEMVLDETWHSDPLVCGQNKVGASGYIGSWLVKLLLQRGYTVRATVRDPNDPKKTEHLLSLDGAKERLHLFKAG
LLEEGAFDPVIDGCVGVFHTASPVQLSATDPQAEIVEPAVKGTLNVLKSCVKFPTVKRVVLTSSMSSVSFSRTPIT
SDMVLDETWYSDPLVCEQNQRWYPLSKILAEEAAWKVAKENGIDLVSMNPGIVIGPLLQPTLNGSVELIANIISGI
QTPFLNYTFVDVRDVASAHIQAFEVPSANGRYCLVGQVADGPDTLQILRQLYPTLCLPEFGNPSDSKYQVSKEKAK
RLGINFLPLEESLRDTIESLKEKGFLNI
>PbCCR-like7
MSKQGEVVCVTGGSGCIGSWLVRLLLHRDYTVHATVKDLKDEGETKHLEALVEGAESRLRLFQIDLLDYNSILAAV
NGCSGVFHLASPCIIDPVHDPEKELLDPAITGTLNVLTAAKQAGVGRVVLTSSTSAITPSPSWPSDKVNGEDCWTD
TDYCKQKGLWYPLSKTLAEKAAWEFAKEKGLDVVVVNPGAVMGPVISPRLNSSMLMLLRILEGCTETYEDFFMGSV
HFKDVALAHILVYENKSATGRHLCLEAISRYGDFVAKVAELYPEYTVRSLPKDTQPGLLREKNGAKKLMNLGLEFI
PMDQIIKDAVESLKSKGFIL
>PbCCR-like8
MTKVVCVTGASGYIASWLVKLLLQRGYIVKATVREPNDSMKTEHLLSLDGAKERLQLFKANLLEEGSFDSAVDGCE
GVFHTASPVQFSATDPQPTVKRVILTSSMAAVGVNGRPLNPDVVIDETWYSDPAICEQLKEWYFLSKTLAEEAAWK
FSKENGIDLVTINPSYVIGPLLQPTLNLTVEMILNLKNDIPSIISSNYPSSDVRDVASAHVQAFEVPSASGRYCLV
GHVTPTSKALSILHELHPTFFPPEKCDDDKPCEPGYQISKEKAKSLGVDFLPLKESLRDTIESLKEKGFLKL
>PbCCR-like9
MSKQGEVVCVTGGSGCIGSWLVRLLLHRDYTVHATVKDLKDEGETKHLEALVEGAESRLRLFQIDLLDYNSILAAV
NGCSGVFHLASPCIVDQVHDPEKELLDPAIKGTLNVLTAAKHAGVSRVVLTSSISAITPSRSWPSDKVKGEDCTDI
DYCKQKGLWYPLSKTLAEKAAWEFAKEKGLDVVVVNPGTVMGPVISPRLNASMLMLVRLLEGCTETYEDFFMGSVH
FKDVAQAHILVYENKSATGRHLCVEAISHYGDFVAKVAELYPEYKVPR
>PbCCR-like10
MPEYCVTGGTGFIAAYLVKALLDKGHVVRTTVRNPEDGGKVGYLWEFNGANERLKVLKADLMVEGSFDEAVRGVDG
VFHTASPVLVPYDDNANLIDPCIRGTLNVLNSCSKARVKRVVLTSSCSSIRYRYDAQQASPLNESHWSDAEYCKHY
NLWYAYAKTTAEKEAWRIAGESGMDLVVVNPSFVVGPLLAPQPTSTLYMILAILKGSRGEYPNTTVGFVHIDDVVA
AHILAMEESKASGRLICSSSVAHWSEIIEMLRAKYPSYPYENKCSSMEGDSNPHSMDTSKIAQLGFPPFKTLETMF
DDCITSFQQKGFL
>PbCCR-like11
MSKQGEVVCVTGGSGCIGSWLVRLLLHRDYTVHATVKDLKDEGETKHLEALVEGAESRLRLFQIDLLDYNSILAAV

Biology Open • Supplementary information

NGCSGVFHLASPCIVDQVHDPEKELLDPAIKGTLNVLTAAKQAGVSRVVLTSSISAITPSPSWPSDKIKGEDCWTD
IDYCKQKGLWYPLSKTLAGKAAWGFAKEKGLDVVVVNPGTVMGPVISPRLNASMLMLLRILEGCTETYEDFFMGSV
HFKDVALAHILVYENKSATGRHLCLEAISRYGDFVAKVAELYPEYTVRREGLVHKMPLQMKSGAYLMF
>PbCCR-like12
MDEKKKEEAVCVTGANGFIGSWLVKTLLEEGYTRIHASIFPASDPSHLFSLPGADCHTITVFEADLLDADAVARAV
QHCQGVFHVASPCTLDDPTDPQAELVLPAVQGTLNVLQAASKFGVSRVVLTSSISAMVPNPSWPPHKPFDESSWTN
LDYCKARQKWYPVSKTEAEKAAWEFAEKHGLDVVAIHPATCLGPLLQPSLNASSAVLLNLLHGSNDTQEHHWLGAV
HVQDVAKAQVLLFESRAASGRYLCTNGIFQFANFALTVSKLFPQFPIHRFSGETQPGLKECKDAAKRLIELGLVFK
PVEDAVQDTVESLKAKGFLKPEISPSNSK
>PbCCR-like13
MPEYCVTGGTGFLAAYLVKALLDKGHVVRTTVRNPEDVGKVGYLWEFNGARERLKMLKADLMVEGSFDEAVRGVDG
VFHTASPVLVPYDDNANLIDPCIRGTLNVLNSCSKARVKRVVLTSSCSSIRYRYDAQQASPLNESHWSDAEYCKHY
NLWYAYAKTTAEKEAWRIAGESGMDLVVVNPSFVVGPLLAPQPTSTLYMILAILKGSSGEYPNTTVGFVHIDDVVA
AHILAMEESKASGRLICSSSVAHWSEIIEMLRAKYPSYPYENK
>PbCCR-like14
MEMMEAPAEEEKERVCVTGAGGFVGSWIVKLLLSSDYLVHGTVRKPPGDSKYAHLSKLEKASENLKLFKADLLDYN
SLCSAVAGCSGVFHVASPVIPSASVTNPQARVELIEPAVKGTLNVLSACLEAKVKRVVVVSSISALFVSLDWPKGQ
VKDEACWSVPEYMTATKKWYLLSKTLAEREALEFGKRTGLEIITVLPSLNLGPILQPTMNASSWFLAKVLKGGFES
IGYNYWTVVAVRDVAEAVLLVYKKSEAGERYICTAYAIGIKDLVDNYLRVAYPNYNYPHDLSHPEEEEKSVSSDRL
QTLGWSYRPLKETLIDSIESYRKASVLD
>PbCCR-like15
MAGEALTGTRLYCQSHYDSKKTEHLLSLDGAKERLQLFKANLLEEGSFDAAVDGCEGVFHTASPVQFSAADPQAEL
IGPAVKGTLNVLKSCVKFPTVKRVVLTSSMAAVIVNGRPLNPDVVVDETWYSDQAVCAQLKEWYFLSKTIAEEAAW
KFSKENGIDLVTINPSYVIGPLLQPTLNLTVKMILNLKNDVHDVISSNYISSDVRDVAYAHVQAFEVPSASGRYCF
VGHVTPVSKVLNILHKLHPTFFPPEKCEDDKPCEPGFQISKEKAKSLGVDFLPLEVSLRDTIESLKEKGFLQL
>PbCCR-like16
MEMMAAPAEEGKERVCVTGAGGFVGSWIVKLLLSSDYLVHGTVRNPPSKYAHLSKLEKASENLKLFKADLLDYNSL
CSAVAGCSGVFHVASPVIPSASVTNPQARVELIEPAVKGTLNVLSACLKAKVKRVVVVSSISALFVSLDWPKGQVK
DETCWSVPEYMTATKKWYLLSKTLAEREASEFGKRTGLEIITVLPSLNLGPILQPTMNASSWFLAKVLKGGFESIG
YNYWTVVVVRDVAEAVLLVYKKSEAGERYICTAYAIGIKDLVDNYLRVAYPNYNYPHDLSHPEEEEKSVSSD
RLQRLGWSYRPLKETLIDSIESYRKASVLD
>PbCCR-like17
MAGGTTDQKEKVCVTGAGGFTGSWLVNLLLSKDYVVHGTVRQPGDSKYDHLNKLEKASENLKLFKADLLDYDSLRL
AIEGCSGVFHVASPIPTYLKDSDPEARIFQIMLSIKGTLNVLKACKEAKVKRVVVVSSLAAVVMNLEWPKDQVKDE
NCWSVPEYMKTTKKWYYLSKTEAEREALEFGKRNGLEVVTVCPSLILGPILQSTRKCSSFLIVMTIIVCVHTRATL
YECVGDLESLPCNYWTFVDVRDLAEALLLAYKKSEAAGERYICHSHSIGIRDVVEKYLRPTYPDYKYPKNLT
YAEEEVQHISSEKLQKLGWTFRPVEETLNDSIESHRKAGIVG
>PbCCR-like18
MAGGTTDQKEKVCVTGAGGFTGSWLVNLLLSKDYVVHGTVRQPDLLDYDSLRLAIEGCSGVFHVASPIPTYLKDSD
PEARIFQIMLSIKGTLNVLKACKEAKAKRVVVVSSLAAVVMNLEWPKDQVKDETCWSVPEYMKTTKKWYYLSKTEA
EREALEFGKRNGLEVVTVCPSLILGPILQSTRKCSSFLIVMTIIGDLESLPCNYWTFVDVRDLAEALLLAYKKSEA
AGERYICHSHSIGIRDVVEKYLRPTYPDYKYPKNLTHAEEEVQHISSEKLQKLGWTFRSVEETLNDSIESHR
KAGIVG
>PbCCR-like19

Biology Open • Supplementary information

MASPVLMSVTDPQAELIDPAVKGTLNVLRSCAKFPSIKRVVLTSSIVSIVFTGKPLSADTVVDESWFSDPAFCEKA
KLWYALSKTLAEEAAWKFAKENGIDLVALNPGMAIGPLLQPTLNSSSEPLLKLFDGTGKFPNVTHRWVDVRDIANA
HILAFENPSASGRYSIVGSVAHYSVAVKILRDLFPALSLPEKCADDQPFAPTYHISKERAETLGVVCTPLERENEC
RNKEVVCVTGASGYVASWLVKLLLQRGYAVKATVRDPNDTNKTEHLLSLGGAKEGLHLFKAYLLEEGSFDAVVDGC
VGVFHTASPAQFSVTDPQAAVKGTLNVLESCVKFPAVKRVVITSSMAAVMMTGKPLTSDVVLDETYYSYSLFCEKI
KLSRQPGNLPKEMGLPWLQYIQGLQLVHYYGQLLISPSSFFRISWVVQFIPFVFNNILCDASYKLCFVSRSWSRIE
TPFINYPFVDVRDIASAHIQTFEVPSAKGRYCLVAQVADGPDTLKILQQLYPTLSLPDFGNPPDSKFQVSIEKARG
LGITFLWRVSETLLKASRRRASSTFEFYYLQQFVTRVLDQLIGK

>PbCCR-like20

MDETWYSDPLFCETTKQWYPLSKTLAEEAAWKFAKGNGIDLVTIHPGNAIGPPLQPTLNLSVEFLLNLMSGIETPF
INYTFVDVRDVAFAHIQAFEVPSASGRYCLVAQVADGPDTLKILRELYPTLTLPEPGNPSGSKFQVSKEKAKCLGI
TFLPLETSLKDTVESLMEKGFLKF

>PbCCR-like21

MAPAAAVCVMDASGHVGASLTERLLQKGYTVHAARQRHGEARLGGICCDKTKLKVFDLDPFDYQSILDALKGCSGL
FYSFEPPQDQPDCDEYIAEVEVRAAHNVLEACARTETIDKIVFTSSAAAVFWRNDRKSTALDLDLDERHWSDVNFC
RSFKLWHALSKTLAEKTAWALAMDRSLNMVSMNVGLLMAPDLSITNPYMKGAAEMYEDGVLVTVDTDFLVDAHICV
FEDVSSYGRYLCFNNIINRAEDALELARKLTPSAPSYPQGQDQDMSIPQQRISNKKLNKLMVEFKSKSQEC

>PbCCR-like22

MPPAAEAIRSFDDDDDADETSPCSKTTTVCVMDASGRLGSTLVHRLLHRGYAVHAALQKHHTECFDDELVKKKKLR
VFRSDPFDYHSIVDALKGCSALFYSFEPPSDHPTYDEFMAEAEVRAAHNVLEACAQTDTIHKVVFTSSVTAVIWRD
DRNASSASSSSRGIDDERNWSDVNFCRKFKLWHALSKTLAERTAWALAMDRGLNMVSINGGLLLGPDLTITDPYLK
GTAEMYQDGLLVTVDLKSIVDAHISVFEDVSSYGRYVCFDRVINCYKDAVELARMLLPPSQTPSLQTQPQSQSQSL
EEAETMVGYQQKISNKKLNKLMVNFDTALQLRD

>PbCCR-like23

MGIVGLEERENIELQEFCRMLMASAGLHRRKDGRQQPNNKRFDLKGDDEEDMLVCVTSGVSFLGLALVDRLLRRGY
SVRLVVDNQEDVEKLREMRTGGSTDDRISAVMAKLTDDDDVESLSHAFDGCRGVFHTSAFVDPAGLSGYTKSMAEI
EVKASENVMKACAVTPSVRKCVFTSSLLACVWQDSTRNDLSPVINHDSWSTESLCIDKKLWYALGKLRAEKAAWKI
AEEKGVKLATICPALITGPEISTRNPTATLAYLKGAQQMYQSGVLATVDITRLAEAHVGVFEAMNKAAFGRYICFD
QVVDGEEEAEKLAEATSMLKNKFVGNGGSNIVQNRFELSNRKLTNLLSGRVHCCYSS

>PbCCR-like24

MAPAAASDSSNKAVCVMDASCHLGTSLAECLLQKGYTVHAALQKHGESRLGGISFDKKKLKVFNLDPFDYRSILDA
LIGCSGLFYSFEPPQDQPDYDEHMTEVEVRAAHNVLEACARTETIDKVVFTSSATAVLWRNDRKSTTLDLDLDERH
WTNVNFCHSFNLWHALSKTLAEKTAWALAMDTSLNMVSMNVGLLMAPDLSITNPYLKGAAEMYEDGVLVTVDTDFL
VDAHICVFEDVSPYGRHLCFNNIINRSEDAMELARKLTPATPSYPERQDQDMRFPQQRISNKKNKLMEEFK
SKSQEC

>PbCCR-like25

MGIVGLEERVNIEVQEFCSTLVASAGLHRRKDDHQQPDHKRFDSRGDYEQDMLVCVTSGVSFLGLALVDRLLHRGY
SVRLIVDKQEDVEILREMRTRGATSDRISAFIAKLTDTDVVESLSHAFHGCRGVFHTSAFVDPAGLSGYTKSMAEI
EVKASENVMKACAMTPSVRKCVLTSSLLACVWQDSYQNLNFSPVINHNHWSSESLCIDKKLWYALGKLRAEKAAWK
IAEEKGLKLTTICPALITGPEISTKNPTATLSYLKGAQEMYQSGVLATVDITRLAEAHVGVFEAMAAFGRYIWFDR
VIDEEEEAEKLAEETGMSKNKILGNGGSNVVQNRFELSNKKLTNLLTGRVHCCYNS

>PbCCR-like26

MAMEEMEVPKHLMSSSGEEHKICVKNAFSCSLDARDRRLVCVTSGNSHLGSHIVRALLARGYLVRVTIQNEVDFED
MKKLMRGEEISQLECVVVAKMGDLDNLCDAFRGCHAIFHTSSFIDPHGTSGYSEKMAFLETEGARNVIEACGRAAY

Biology Open • Supplementary information

AKRCIFTSSLLAAIWTGENYEEKKIIDEHSWTNVEFCRENKLWLALGKTTAEMACWRKSKEMKVNLVTVCPGLLMD
ASHPNAPISTSIPYLKGKFSRGKTMLKHGRLAVDDVKKAAKAHVCVYEAMDHGASGRYLSFGKVVTKMAEAIELEN
GLKIHGLLSGEGLEDLGEETDEELQSMLSNAKLARLIGASQKLSCK
>PbCCR-like27
MDASGCLGSSLVHRLLHRGYAVHAALQTDHRCFDDELVKKKKVSVFRSDLFDYHSIMDALKGCSALFYSFEPPSDH
PTYDEFMAEVEVRAAHNVLEACAQTDTIHKVVFTSSVTAVIWRDDRTTSPSSSHDIDERNWSDVNFCKKFKLWHAL
SKTLAEKTAWALAMDRGLNMVSVNGGLLMSPDLTITNPHLKGAAEMYQDGLLVTVDLEFIVDAHISVFEDASSYGR
YVCFDRVINCYKDAVELARMLLPPSQTSFLQTQIQSQSQTPSFEETETMVVYQQKISNEKLNKLMVDFDTAL
QLSR
>PbCCR-like28
MVDLEVRGAINVVEACAQTEKVEKVIFTSSLTAAIWRDNISYQKDVDEKSWSDHQFCRKLKLWYALAKTLSEQAAW
ALAMDRMLRMVSINSGLVLGPGVTQQNPRTTMSYLNGAAQMYENGVLAFVDVNFLVDIHIRALEDESTCGRYLCFN
QIVNTEDEAIKLAHSLSPLISLPPRYEYQGSEVHTERLRNEKLSKLVEAMPIN
>PrCCR(AFC38436.1)
MTAGKQTEEGQTVCVTGAGGFIASWLVKLLLERGYTVRGTVRNPEDQKNAHLKQLEGAEERLTLVKADLMDYNSLL
NAINGCQGVFHVASPVTDDPEEMVEPAVNGTKNVLDACAVAGVRRVVFTSSIGAVYMDPSRDYDALVDENCWSNLD
YCKETKNWYCYGKTVAEKAAWERAKDKGLDLVVVNPCVVLGPVLQSSINASIIHILKYLTGSAKTYANSVQAYVHV
RDVAEAHILVYESPSASGRYLCAESVLHRGDVVDLLASMFPQYPIPTKVKEDGKPRVKPWKVSNQKLKDLGLEFTP
AKQCLYETVISLQEKGHISK
>PaCCR(CAK18610.1)
MTAGKQTGAGQTVCVTGAGGFIASWLVKLLLERGYTVRGTVRNPEDQKNAHLRQLEGAEERLTLVKADLMDYNSLL
NAITGCQGVFHVASPVTDDPVQMVEPAVNGTKNVLDACAEAAVRRVVFTSSIGAVYMDPTRDYDALVDESCWSNLD
FCKDTKNWYCYGKAVAEKAAWDRAKEKGLDLVVVNPCVVLGPVLQSSINASILHILKYLTGSAKTYANSVQAYVHV
RDVAEAHILVYESPSASGRYLCAESVLHRGDVVELLEKMFPQYPIPTKCKDDGKPRVKPWKVSNQKLKDLGLEFTP
AKQCLYETVISLQEKGHI
>AtCCR1(At1g15950)
MPVDVASPAGKTVCVTGAGGYIASWIVKILLERGYTVKGTVRNPDDPKNTHLRELEGGKERLILCKADLQDYEALK
AAIDGCDGVFHTASPVTDDPEQMVEPAVNGAKFVINAAAEAKVKRVVITSSIGAVYMDPNRDPEAVVDESCWSDLD
FCKNTKNWYCYGKMVAEQAAWETAKEKGVDLVVLNPVLVLGPPLQPTINASLYHVLKYLTGSAKTYANLTQAYVDV
RDVALAHVLVYEAPSASGRYLLAESARHRGEVVEILAKLFPEYPLPTKCKDEKNPRAKPYKFTNQKIKDLGLEFTS
TKQSLYDTVKSLQEKGHLAPPPPPPSASQESVENGIKIGS
>AtCCR2(At1g80820)
MLVDGKLVCVTGAGGYIASWIVKLLLERGYTVRGTVRNPTDPKNNHLRELQGAKERLTLHSADLLDYEALCATIDG
CDGVFHTASPMTDDPETMLEPAVNGAKFVIDAAAKAKVKRVVFTSSIGAVYMNPNRDTQAIVDENCWSDLDFCKNT
KNWYCYGKMLAEQSAWETAKAKGVDLVVLNPVLVLGPPLQSAINASLVHILKYLTGSAKTYANLTQVYVDVRDVAL
GHVLVYEAPSASGRYIFAETALDRGEVVEILAKFFPEYPLPTKCSDEKNPRAKPYKFTTQKIKDLGLEFKPIKQSL
YESVKSLQEKGHLPLPQDSNQNEVIIES
>AtCCR-like1(At1g76470)
MAVKQKVCVTGAGGFIASWLVKFLLSRGYTVHGTVRDPCDEKNDHLRKLDNASKNLKLFKADLFDDEGLFSAIDGC
SGVFHIASPVPFEGVPLTEEELIKPALTGTKNVLEACTETKVQKVVVVSSIAAVVYNPKWPQDVAKDEDCWSDTQY
LHSLEGYWRYYYLAKTLTEREALEWSKRNFADVVTLCPSVIIGPRLQSTLNSSSLGLLKFIKGGIKSLLSDELYLV
DVRDVADALLLVYENREATGRYICNSHSLYTDSLMEKLKNMYPKRNFPESFTEVKEKEVRPLSAEKLKNLGWKFRP
LEETIDDSVVSFEAAGDLPKA
>AtCCR-like2(At2g02400)

Biology Open • Supplementary information

MAKETVCVTGANGFIGSWIIRTLIEKGYTKIHASIYPGSDPTHLLQLPGSDSKIKIFEADLLDSDAISRAIDGCAG
VFHVASPCTLDPPVDPEKELVEPAVKGTINVLEAAKRFNVRRVVITSSISALVPNPNWPEKVPVDESSWSDLDFCK
SRQKWYPISKTLAEKAAWEFSEKHGTNIVTIHPSTCLGPLLQPNLNASCAVLLQLLQGSTETQEHHWLGVVHVKDV
AKGHVMLFETPDASGRFLCTNGIYQFSEFAALVSKLFPEFAVHKFDKETQPGLTSCNDAAKRLIELGLVFTAVEDA
VKETVQSLRDKGFL
>AtCCR-like3(At2g33590)
MADVHKGKVCVTGAGGFLGSWVVDLLLSKDYFVHGTVRDPDNEKYAHLKKLEKAGDKLKLFKADLLDYGSLQSAIA
GCSGVFHVACPVPPASVPNPEVELIAPAVDGTLNVLKACIEANVKRVVYVSSVAAAFMNPMWSKNQVLDEACWSDQ
EYCKKTENWYCLAKTRAESEAFEFAKRTGLHLVSVCPTLVLGPILQQNTVNASSLVLLKLLKEGFETRDNQERHLV
DVRDVAQALLLVYEKAEAEGRYICTSHTVKEEIVVEKLKSFYPHYNYPKKYIDAEDRVKVSSEKLQKLGWTYRPLE
ETLVDSVESYRKAKLVD
>AtCCR-like4(At2g33600)
MAVVQKGKVCVTGAGGFLGSWVVNHLLSRDYFVHGTVRDPGNEKYAHLKKLDKAGDKLKLFKADLLNYGSLQSAIA
GCSGVFHVACPVPSASVPNPEVDLIAPAVDGTLNVLKACVEAKVKRVVYVSSVSAVAMNPMWSKSQVLDETAWSDQ
DYCKKTENWYSLSKTRAESEAFEFAKRTGLDLVSVCPTLVLGPVLQQHTVNASSLVLLKLLKEGYESRNNQERHLV
DVRDVAQALLLVYEKAEAEGRYICIGHTVREQEVAEKLKSLYLNYNYPKRYIEADGKVKVSSEKLQKLGWTYRPLE
ETLVDSVESYRKAKLVD
>AtCCR-like5(At5g58490)
MLTDEREVVCVTGASGCIGSWLVHQLLLRGYSVHATVKNLQDEKETKHLEGLEGAATRLHLFEMDLLQYDTVSAAI
NGCSGVFHLASPCIVDEVQDPQKQLLDPAVKGTINVLTAAKEASVKRVVVTSSISAITPSPNWPADKIKNEECWAA
EDYCRQNGLWYPLSKTLAEKAAWEFAEEKGLDVVVVNPGTVMGPVIPPSLNASMHMLLRLLQGCTETYENFFMGSV
HFKDVALAHILVYEDPYSKGRHLCVEAISHYGDFVAKVAELYPNYNVPKLPRETQPGLLRDKNASKKLIDLGLKFI
SMEEIIKEGVESLKSKGFIS
>HvCCR1(BAK07589)
MTVVDAAAAVAQELPGHGQTVCVTGAAGYIASWLVKLLLERGYTVKGTVRNPDDPKNAHLKALDGAAERLVLCKAD
LLDYDAICAAVEGCHGVFHTASPVTDDPEQMVEPAVRGTEYVIDAAADAGTVRRVVFTSSIGAVTMDPNRGPDVVV
DESCWSDLEFCKKTKNWYCYGKAVAEQAAWEKARARGVDLVVVNPVLVVGPLLQPTVNASAAHILKYLDGSARKYA
NAVQAYVDVRDVAGAHLRVFEAPQASGRYLCAERVLHRQDVVHILAKLFPEYPVPTRCSDEVNPRKQPYKMSNQKL
QDLGLKFTPVNDSLYETVKSLQEKGHLPVPRKDILAPQLDGATA
>HvCCR2(BAJ97892)
MTVGGEATGDGQTVCVTGAGGYIGSWIVKLLLEKGYAVRGTVRNPDDAKNAHLRALAGAAERLVLCKADLLDADAL
RAAIAGCHGVFHTASPVTDDPEEMVEPAVRGTRYVIDAAAESGTVRRVVLTSSIGAVAMDPSRAPDAVVDESCWSD
LEFCKKTKNWYCYGKTVAEREAWEAAAARGVDLVVVNPVLVQGPALQPAVNASLTHVLKYLDGSAKTYANAVQAYV
HVRDTAAAHVVVFEAPAAAGRYLCVADGAVLHREDVVTILRKFFPEYPIPSRCSDSVNPRKRPYKMSNQRLRELGL
EFTPVAQCLYDTVVSFQEKGILPVPPAPAQPAMKEIN
>HvCCR3(BAK01264)
MGIDRANTANCVATGRGRTVCVTGAGGFIASWLVKLLLEKGYAVHGTVRNPDDVARNAHLRGLEGAAERLTLFRVD
LLDKESLVAAFRGCQGVFHTACPVTDDPEKMIEPAVSGTRNVINAAAEVGGIRRVVMTSSIGAVYMDPRRSPDEEA
DETCWSDLEFCKNTKNWYCYAKTVAEQAAWELAKERKLDLVVINPSLVLGPLLQTAVNASTWHIAKYLDGSVQTCA
NAAQAYVHVRDVADAHARAYETPEAHGRYLCAGRTLHRAEVCRTLAKFFPEYPVPMRCKEGTGEMKKGCRFSSRRI
MELGVGITPASQCLYDIVISLQDKGILPRRGADMS
>HvCCR4(BAJ89483)
MAAVVCVTGAGGFIGSWIVKLLLARGYAVRGTSRRADDPKNAHLWALDGAAERLTMVQVDLLDRASLRAAFHGCDG
VIHTASPMHDTPEEIIEPVITGTLNVVEMAAGAGVRRVVLSSTIGTMYMNPHRDPDAPLDDSCWSDLDYCKQTKNW

Biology Open • Supplementary information

YCYAKTIAERGAWEAARSLGLDLAVVIPVVTLGELLQPSMNTSTKHILKYLTGEAKAYVNESHAYVHVKDAAEAHV
RVLQAPNAGGRRYVCAERTLHRGELCRILAGLFPEYPIPTRCKDEVNPPKKGYKYTNQPLKDLGMKFTPVQEYLYE
AVNSLQEKGFIKKASGTKVLASRGGSPPQNSPAPMFMSKL
>OsCCR1(Os09g25150)
MTVVVVADDAAAAAAAAQQQEELPPGHGQTVCVTGAAGYIASWLVKLLLERGYTVKGTVRNPDDPKNAHLKALDGA
DERLVLCKADLLDYDSIRAAVDGCHGVFHTASPVTDDPEQMVEPAVRGTEYVIKAAAEAGTVRRVVFTSSIGAVTM
DPNRGPDVVVDESCWSDLEFCKKTKNWYCYGKAVAEQEACKAAEERGVDLVVVSPVLVVGPLLQPTVNASAVHILK
YLDGSAKKYANAVQAYVDVRDVAAAHVRVFEAPEASGRHLCAERVLHREDVVHILGKLFPEYPVPTRCSDEVNPRK
QPYKMSNKKLQDLGLHFIPVSDSLYETVKSLQEKGHLPVLSKEIPEELNGVPA
>OsCCR2(Os08g34280)
MTVIDGAVAADAGGAAAAVVQPGNGQTVCVTGAAGYIASWLVKLLLEKGYTVKGTVRNPDDPKNAHLKALDGAGER
LVLCKADLLDYDAICRAVAGCHGVFHTASPVTDDPEQMVEPAVRGTEYVINAAAEAGTVRRVVFTSSIGAVTMDPN
RGPDVVVDESCWSDLDYCKETRNWYCYGKAVAEQAAWEAARRRGVELVVVNPVLVIGPLLQPTVNASVAHILKYLD
GSASKFANAVQAYVDVRDVAAAHLLVFESPSAAGRFLCAESVLHREGVVRILAKLFPEYPVPTRCSDEKNPRKQPY
KMSNQKLRDLGLEFRPASQSLYETVKCLQEKGHLPVLAAEKTEEEAGEVQGGIAIRA
>OsCCR3(Os08g17500)
MGVEKTTANGGSGAAAVSGGGRTVCVTGAGGFIASWLVKLLLEKGYAVRGTVRNPDDAAKNAHLMALAGAAERLTL
VRAELLDKESLAAAFAGCEGVFHTASPITDDPEKMIEPAVSGARNVITAAADAGGVRRVVMTSSIGAVYMGGGGGE
EVDETCWSDLDHCRDTGNWYCYAKTVAEQAAWELAKERRLDLVVVNPSLVLGPLLQRGVNASTWHVLKYLDGSART
YADAAQAYVHVRDVADAHARAYESPAARGRYLCAGRTLHRGEVCRILAALFPGYPVPTRCKGDAGETAEGCRFSSR
KLAELGVAVMPASQCLYDTVVSLQDKGLLPFVPAAAMP
>OsCCR4(Os09g04050)
MPTDETAAAAPATTALSGHGCTVCVTGAGGFIASWLVKRLLEKGYTVRGTVRNPMDPKNDHLRALDGAGERLVLLR
ADLLDPDSLVAAFTGCEGVFHAASPVTDDPEKMIEPAIRGTRYVITAAADTGIKRVVFTSSIGTVYMNPYRDPNKP
VDDTCWSDLEYCKRTENWYCYAKTVAEQGAWEVARRRGVDLVVVNPVLVLGPLLQATVNASTEHVMKYLTGSAKTY
VNAAQAYVHVRDVAEAHVRVYDCGGARGRYICAESTLHRGDLCRALAKLFPEYPVPSRCKDEAAPPVKGYLFSNQR
LRDLGMDFVPVRQCLYETVRSLQDKGLLPVLPPTADDHHHPSS
>OsCCR5(Os02g08420)
MAAAVVCVTGAGGFIGSWIVKLLLARGYAVRGTSRRADDPKNAHLWALDGAAERLTMVSVDLLDRGSLRAAFAGCH
GVIHTASPMHDDPEEIIEPVITGTLNVVEVAADAGVRRVVLSSTIGTMYMDPRRDPDSPLDDSFWSDLDYCKNTKN
WYCYAKTIAERKAWEVARGRGVDMAVVIPVVVLGELLQPGMNTSTKHILKYLTGEAKTYVNESHAYVHVVDAAEAH
VRVLEAPGAGGRRYICAERTLHRGELCRILAGLFPEYPIPTRCRDEINPPKKGYKFTNQPLKDLGIKFTPVHEYLY
EAVKSLEDKGFIKKTSNTKELHRQSSPPQNSPASMLMSKL
>OsCCR6a(Os02g56680)
MSSNFEANNNNGEKQLVCVTGAGGFIGSWVVKELLIRGYHVRGTARDPADSKNAHLLELEGADQRLSLCRADVLDA
ASLRAAFSGCHGVFHVASPVSNDPDLVPVAVEGTRNVINAAADMGVRRVVFTSSYGAVHMNPSRSPDAVLDETCWS
DYEFCRQTDNLYCCAKMMAEMTATEEAAKRGLELAVVVPSMTMGPMLQQTLNFSSNHVARYLMGTKKSYPNAVAAY
VDVRDVARAHVLVYERPDARGRYLCIGTVLHRAELLRMLRDLFPQYPATAKCEDDGKPMAKPYKFSNQRLKDLGLE
FTPLRKSLHEAVLCMQQKSHLPLIYPVPKRAYL
>OsCCR6b(Os02g56690)
MSCNSAEVAANDGSSNGGEKQQQQEEEVVCVTGAGGFIGSWVVKEHLLRGYRVRGTARDPTKNAHLLALDGAGERL
TLCRADVLDSESLRAAFAGCHGVFHVASPVSNDPNLVPIAVEGTRNVVNAAADMGVRRVVFTSSYGAVHMNPNRSP
DTVLDETCWSDPKFCRQTDVYCYAKTMAEKAAEEEAAKRGVQLAVVLPCVTVGPILHPAINTSINHVVRYLTGAAP
TYPNAVAAYVDVRDVARAHALVYERHDARGRYLCIGAVLHRAHLLQMLKELFPQYPVTSKCKDDGNPMVEPYKFSN

Biology Open • Supplementary information

QRLKDLGFEFTPMRKCLYDAVVCMQQKGHLPLVGTAVPDQNVTSNTSSIV
>OsCCR6c(Os02g56700)
MSSNNSMEANNGDDEKKQEQVVCVTGAGGFIGSWVVKELLLRGYRVRGTARDPRKNAHLLDLEGAKERLTLCRADV
LDFASLRAAFAGCHGVFHIASPVSKDPNLVPVAIEGTRNVMKAAADMGVRRVVFTSSYGAVHMNPNRSPDAVLDES
CWSDPEFCQREDIYCYAKMMAEKTATEEASRRRLQLAVVVPCVTVGPILQPSVNFSCHHVVRYLTGAAATYPNAVA
AYADVRDVARAHVLVYEHHGARGRYLCIGTVIHRAELLRMLKELFPQYPVTSKCEDEGNQMVKPYKFSNQRLRDLG
LEFTPLRKSLHEAIECLQRKGHLPVVTVAQQRACL
>OsCCR6d(Os02g56720)
MSSISNDNNGDQKRQQQQQLVCVTGAGGFIGSWVVRELLLRGYRVRATVRDPADRKNAHLLALEGAHERLSLRRAD
VLDFAGLLAAFAGCHGVFHVACPLSNRDPELMAVAVDGTRNVMNAAADMGVRRVVFTSSYGAVHMNPNRSPDAVLD
ESCWSDPEFCRQKDMYCYAKTMAEMAATEEAAKRGLELAVVVPSMTMGPMLQRALNLSSTHVANYLTGAKKSYPNA
VAAYVDVRDVARAHALVYERHDARGRYLCIGAVLHRAQLLQMLMDLFPQYTIASKCDDKGKPMVKPYEFSNQRLKD
LGLEFTPLRKSLYDAVMCMQRNGHLPVVLP
>OsCCR7(Os02g56460)
MSSNFEANNNNNGEKQLVCVTGAGGFIGSWVVKELLIRGYHVRGTARDPADSKNAHLLELEGADERLSLCRADVLD
AASLRAAFSGCHGVFHVASPVSNDPDLVPVAVEGTRNVINAAADMGVRRVVFTSSYGAVHMNPNRSPDAVLDETCW
SDYEFCKQTDNLYCCAKMMAEMTATEEAAKRGLELAVVVPSMTMGPMLQQTLNFSTNHVARYLMGTKKSYPNAVAA
YVDVRDVARAHVLVYERPEARGRYLCIGTVLHRAELLRMLRELFPRYPATAKCEDDGKPMAKPYKFSNQRLKDLGL
EFTPLRKSLNEAVLCMQQKGHLPLIYPVPKRAYL
>TaCCR1(ABE01883.1)
MTVVAAAAAAAAQELPGHGQTVCVTGAAGYIASWLVKLLLERGYTVKGTVRNPDDPKNAHLKALDGAAER
LVLCKADLLDYDAICAAVEGCHGVFHTASPVTDDPEQMVEPAVRGTEYVINAAADAGTVRRVGVTSSIGAVTMDPN
RGPDVVVDESCWSDLEFCKKTKNWYCYGKAVAEQAAWEKAAARGVDLVVVNPVLVVGPLLQPTVNASAAHILKYLD
GSAKKYANAVQAYVNVRDVAAAHVRVFEAPGASGRHLCAERVLHREDVVHILGKLFPEYPVPTRCSDEVNPRKQPY
KMSNQKLQDLGLQFTPVNDSLYETVKSLQEKGHLPAPRKDILPAELDGATA
>TaCCR2(AAX08107.1)
MPIEAADNVPAELPGHGRTVCVTGAGGFIASWLVKRLLQKGYNVRGTVRNPVDPKNDHLRAFDGAADRLVLLRADL
MEPETLVEAFTGCEGIFHAASPVTDDPEKMIEPAIRGTKYVITAAADMGIKRVVFTSTIGTVYMNPNRDPSKPVDD
TCWSDLEYCKKTANWYCYAKTVAEQDALETARQRGIELIVVNPVLVLGPLLQPTVNASTEHVMKYLTGSAKTYVNA
AQAYVHVKDVAEAHVRVYEAPGAHGRYICAEGTTLHRGELCRVLCKLFPEYPVPTKCKDEVNPPVKGYKFTNQRLK
DLGMEFVPVLQSIYETVKSLQEKGMLPVLPPGDDVRDNLHEQLMMK
PAQLLRN
>ZmCCR1(NP001105488.1)
MTVVDAVVSSTDAGAPAAAATAVPAGNGQTVCVTGAAGYIASWLVKLLLEKGYTVKGTVRNPDDPKNAHLKALDGA
AERLILCKADLLDYDAICRAVQGCQGVFHTASPVTDDPEQMVEPAVRGTEYVINAAAEAGTVRRVVFTSSIGAVTM
DPKRGPDVVVDESCWSDLEFCEKTRNWYCYGKAVAEHAAWETARRRGVDLVVVNPVLVVGPLLQATVNASIAHILK
YLDGSARTFANAVQAYVDVRDVADAHLRVFESPRASGRHLCAERVLHREDVVRILAKLFPEYPVPARCSDEVNPRK
QPYKFSNQKLRDLGLQFRPVSQSLYDTVKNLQEKGHLPVLGERTTTEAADKDAPTAEMQQGGIAIRA
>PtCCR(AAF43141.1)
MPVDASSLSGQGQTCVTGAGGFIASWMVKLLLDKGYTVRGTARNPADPNNSHLRELEGAQERLTLCKADLLDYESL
KEAIQGCDGVFHTASPVTDDPEEMVEPAVNGTKNVIIAAAEAKVRRVVFTSSIGAVYMDPNKGPDVVIDESCWSDL
EFCKNTKNWYCYGKAVAEQAAWDMAKEKGVDLVVVNPVLVLGPLFQPTVNASIVHILKYLTGSAKTYANSVQAYVH
VRDVALAHILVFETPSASGRYLCSESVLHRGEVVEILAKFFPEYPIPTKCSDEKNPRKQPYKFSNQKLRDLGFEFT
PVKQCLYETVKSLQERGHLPIPKQAAEESVKIQ

>EgCCR(CAA56103.1)
MPVDALPGSGQTVCVTGAGGFIASWIVKLLLERGYTVRGTVRNPDDPKNGHLRELEGASERLTLYKGDLMDYGSLE
EAIKGCDGVVHTASPVTDDPEQMVEPAVIGTKNVIVAAAEAKVRRVVFTSSIGAVTMDPNRGPDVVVDESCWSDLE
FCKSTKNWYCYGKAVAEKAAWPEAKERGVDLVVINPVLVLGPLLQSTINASIIHILKYLTGSAKTYANSVQAYVHV
KDVALAHVLVLETPSASGRYLCAESVLHRGDVVEILAKFFPEYNVPTKCSDEVNPRVKPYKFSNQKLRDLGLEFTP
VKQCLYETVKSLQEKGHLPVPSPPEDSVRIQG
>LlCCR1(ABL01801.3)
MPAAAPAPTAANTTSSGSGQTVCVTGAGGFIASWIVKLLLERGYTVRGTVRNPDDSKNSHLKELEGAEERLTLHKV
DLLDLESVKAVINGCDGIIHTASPVTDNPEEMVEPAVNGAKNVIIAAAEAKVRRVVFTSSIGAVYMDPSRNIDEVV
DESCWSNLEYCKNTKNWYCYGKAVAEQAAWDEAKARGVDLVVVNPVLVLGPLLQSTMNASTIHILKYLTGSAKTYA
NATQAYVHVKDVALAHVLVYEIPSASGRYLCSESSLHRGELVEILAKYFPEYPIPTKCSDEKNPRAKAYTFSNKRL
KDLGLEFTPVHQCLYDTVKSLQDKGHLPLPTK
>PhCCR1(AHX56186.1)
MRSVSGQVVCVTGAGGFIASWLVKILLEKGYTVRGTVRNPDDPKNGHLRELEGAKERLTLCKADLLDYQSLREAIN
GCDGVFHTASPVTDDPEQMVEPAVIGTKNVINAAAEANVRRVVFTSSIGAVYMDPNRDPETVVDETCWSDPDFCKN
TKNWYCYGKMVAEQAAWEEAKEKGVDLVVINPVLVQGPLLQTTVNASVLHILKYLTGSAKTYANSVQAYVDVKDVA
LAHILLYETPEASGRYLCAESVLHRGDVVEILSKFFPEYPIPTKCSDVTKPRVKPYKFSNQKLKDLGLEFTPVKQC
LYETVKSLQEKGHLPIPTQKDEPIIRIQP
>PtrCCR2(ACC63879.1)
MPVDASSLSGQGQTICVTGAGGFIASWMVKLLLDKGYTVRGTARNPADPKNSHLRGLEGAEERLTLCKADLLDYES
LKEAIQGCDGVFHTASPVTDDPEEMVEPAVNGTKNVIIAAAEAKVRRVVFTSSIGAVYMDPNKGPDVVIDESCWSD
LEFCKNTKNWYCYGKAVAEQAAWDMAKEKGVDLVVVNPVLVLGPLLQPTVNASITHILKYLTGSAKTYANSVQAYV
HVRDVALAHILVFETPSASGRYLCSESVLHRGEVVEILAKFFPEYPIPTKCSDEKNPRKQPYKFSNQKLRDLGFEF
TPVKQCLYETVKSLQEKGHLPIPKQAAEESLKIQ
>ZmCCR2(NP001105715.1)
MPTAEATTPVPPELSSGQGRTVCVTGAGGFIASWLVKRLLEKGYTVRGTVRNPVDPKNDHLRALDGAVDRLVLLRA
DLLDPQSLAEAFSGCDGVFHAASPVTDDPEMMIEPAIRGTRYVMAAADDAGVKRVVFTSSIGTVYMNPYRDPSKPV
DDTCWSDLEYCKNTQNWYCYAKTVAEQGAWEVARKRGLDLVVVNPVLVLGPLLQPTVNASTDHVMKYLTGSATTYV
NAAQAYVHVRDVAEAHVRVYEAPHAHGRYICAESTLHRGDLCRVLAKLFPEYPVPTKCKDQVNPPVVGYKFTNQRL
KDLGMDFVPVLQCLYETVTSLQEKGMLPVLPTKQDHDQLGKS
>PbCAD1
MSSQTSSVNENCLGWAATDESGVLSPYKFTRRYCFKIPEGYPLASAAPLLCAGITVYSPMIRHKMNQDGKSLGVIG
LGGLGHLAVKFGKAFGLNVTVFSTSLSKKEEALSLLGADNFVVSSNQEEMTAGKESYISQLLGRDTGICYADVVFP
RNKHGNAMYPMVPGHEIAGIVQEVGPNVHGFKVGDHVGVGTFVNSCRDCDYCRDGLEISCEKGAIFTYNHVDADGT
ITKGGFSSYIVAHERYCFKIPEGYPLASAAPLLCAGITVYAPMIRHKMKQAGKSLDVIGLGGLGHLAVKFGKAFGL
NVTVFSTSLSKKEEALTLLGANNFVVSSNQEKKTALAKSLDFIVDIN
>PbCAD2
MGGLEVERKTTGWAARDSSGILSPYTYNIRNTGPEDVYVKVLCCGVCHSDLHQIKNDLGMSNYPMVPGHEVVGEVV
EVGSDVTKFRAGDVVGVGLLVGCCRNCSPCKTDNEQYCNKKIWSYNDTYTDGKTTQGGFAGALVADQKFVVKIPEG
MVPEQAAPLLCAGVTVYSPLRHFGLNVSGLTGGILGLGGVGHMGVKIAKAMGHHVTVISSSDKKREEALEHVGADA
FLVSSDATKMQEAADSLDYIIDTVPVGHPLEPYLSLLKLDGKLILMGVINTPLQFVSPMVMLGRKKITGSFVGSMK
ETEEMLEFCKEKGLTSMIEVVKMDYVNTAFERLEKNDVRYRFVVDVAGSNLI
>PbCAD3
MGNPEVERTTTGWAARDASGILSPYTYTIRNTGPEDVYIKVLFCGVCHTDTHQAKNQLGMSHYPLVPGHEVVGKVV

EAGSDVTKFRVGDVVGVGLVVGCCRNCNPCNTDNEQYCNKKIWTYADVYTDGKPTQGGFSGAMVVDQKFVVKVPEG
MVPEQAAPLLCAGVTVYSPLRHFGLNVGGLKGGILGLGGVGHMGVKIAKAMGHHVTVISSSERKREEALEHIGADE
YLISSDATKMQEAADSLDYIIDTVPVGHPLEPYLSLLKLDGKLILMGAINTPLEFLSPMVMLGRKTITGSFVGSMK
ETEEVLQFCKERGLTTMIEVVKMDYINTAFERLEKNDVRYRFVVDVAGSNL

>PbCAD4
MVANEHFVVRIPETLPLDAAAPLLCAGITTYSPLRYFGLDKPGIHVGVVGLGGLGHVAVKFAKAMGVKVTVISTSP
NKKEEAIEHLCADSFLVSRDEDQMQAAMGTLDGIIDTVSAVHPLLPLIGLLKSHGKLVMVGAPEKPLELPVFPLLV
GRKIVAGSGIGGMKETQEMIDFAAKHSITADIEVIPMDYVNIAMERLLKGDVRYRFVIDIGNTLKSTL

>PbCAD5
MSTEQEHPKKAFGWAARDSSGVFSPFKFSRETGDKDVMFKVLYCGICHSDLYIAKNEWGRSNYPLVPGHEIVGVVT
EVGSKVQNFNVGDKVGVGCMVGSCKSCESGSDNLENYYPKSILTYGSKYYDGTTAYGGYSDIMVANEHFVVRGLGH
VAVKFAKAMGVKVTVISTSLNKKDEAIEHLHADSFLVSRNKDQMQVATGTLDGIIDTVSALHPLLPWIGLLKSDEK
LVMVGAPKKPLEFLVFYFACGVHFAIAGRKIVAGNNIEGMKETQEMIDFATKHNITADIEVIPMDYMNIAMEHLLK
ADVRYRFVIDIGNTLKSTL

>PbCAD6
MSTEQEHPKKAFGWAARDSSGVFSPFKFSRRHEIVGVVTEVGSRVQKFNVGDKVGVGCMVGSCTSCENCTNNLENY
CPEWILTYGAKYYDGTTTYGGYSDIMVANEHFVVRIPETLPLDAAAPLLCAGITTYSPLRYFGLDKPGMHVGVVGL
GGLGHVAVKFAKAMGVKVTVISTSPNKKVEAIEHLCADSFLVSRDEDQMQAAMGTLDGIIDTVSAVHPLLPLIGLL
KSHGKLVMVGAPEKPLELPVFPLLAAMGTLDGIIDTVSAVHPLLPLIGLLKSHGKLVMVGAPEKPLELPVFPLLVG
RKIVAGSGIGGMKETQEMIDFAAKHSITADIEVIPMDYVNIAMERLLKGDVRYRFVIDIGNTLKSTL

>PbCAD7
MSSQTSSVIGNCLGWAATDESGVLSPYKFARRNLQNDDVSIRITHCGICYADVFFPRNKHGNAMYPMVPGHEIAGI
VQEVGPNVHGFKVGDHVGVGTFVNSCRDCDYCRDGIEICCEKGAILTYNHVDADGTITKGGFSSYIVAHERYCFKI
PEGYPLASAAPLLCAGITVYSPMIRHKMNQDGKSLGVIGLGGLGHLAVKFGKAFGLNVTVFSTSLSKKEEALSLLG
ADNFVVSSNQEEMTALAKSLDFIIDTASGDHPFDLYMSLLKTAGVLVLVGVPSEVKLSPMSLIIGMKSISGS
GTGGTKQTQEMLDFCAAHKIYPNIEIVPIQYVNEALERLIKRDVKDCFKIPESYPLASAAPLLCAGITVYAPMIRH
KMNQAGNSLGVIGLGGLGHLAVKFGKAFGLNVTVFSTSLSKKEEALTLLGADNFVVSSNQEKKTVKFHKADTTLYG
KMFRGI

>PbCAD8
MVANEHFVVHIPDNLPLDGAAPLLCAGITTYSPLRYFGLDKPGVHVGVVGLGGLGHVAVKFAKAMGVKVTFISTSP
NKKDEAIEHLCADSFLVSRDEDQMQAEMGTLDGIIDTVSAVHPLLPLIGLLKSHGKLVMVGAPEKPLELPVILLLS
GRKIVAGSGIGGMKEKQEMIDFAAKNNITADIEVIPIDYVNIAMERLLKADVDLSLILETH

>PbCAD9
MSSENANKGEDCLGWAARDESGVLSPYKFARRAVGSDDVSIKITHCGVCYAEVVWTRNKHGDATYPLVPGHEIAGI
VQEVGSNVRGFKAGDHVGVGTYINSCRNCDYCNDGLEVYCEKEVVYTYNRLDVDGTMTKGGFSTYIVVCERYCFRI
AESYPLASAAPLLCAGITVYSPMIRHKMNQPGKSLGVIGLGGLGHLAVKFGKAFGLNVTVFSTSTSKKEESLTLLG
ADNFVVSSDQEQMKAMAKSLDFIIDTVSADHSFDMYMSLLKTAGVLVMVGAPNEVKLSPMSLILGMKSISGSVAGG
TKEMQEMLDFCAAQKIYPNIEIVPIQYVNEAIERLIKKDVKYRFVVDIENSLKY

>PbCAD10
MSTEQEHPKKAFGWAARDSSGVFSPFKFLRRETGDKEVMFKVLYCGICHSDLYMAKNEWGRSNYPLVPGHEIVGVV
TEVGSKVQIFNVGDKVGVGCMVGLCKSCESCSDNLENYCPKSIFTYGSKYYDGTTVYGGYSDIMVANEHFVVRGLG
HVAVKFAKAMGVKVTVISTSLNKKDEAIEHLHADSFLVSRNKDQMQDGIIDTVSALHPLLPWIGLLKSDEKLVMVG
APKKPLEFLVFYFACGVHFAIAGRKIVAGNNIEGMKETQEMIDFATKHNITADIEVIPMDYMNIAMEHLLKADVRY
RFVIDIGNTLKSTL

>PbCAD11

MAKAPELEHPVKAFGWAARDTSGHLSPFNFSRRSTGDEDVRFKVLYCGICHTDLHNIKNEWGISLYPMVPGHEIVG

EVTEVGSKVSKVKEGDKVGVGCMVGACHACESCNSNLENYCPKMILTYGSIYADRTVTYGGYSDTMVANERYIVRF

PENLPLDAGAPLLCAGITVYSPLKYFGLAEPGKHVGIVGLGGLGHVGVKFAKAFGAKVTVISTSPSKKDEALKRLG

ADAFVVSRDPQQMQAAIGTLDGIIDTVSAAHPIVPLLGLLKPHGKLILVGVADKPLDLHVFPLIMGRKSVAGSGIG

GMKETQEMIDFAAKHNITAEVEVISMDYVNTAMERLAKNDVRYRFVIDVGNTLAASKD

>PbCAD12

MVESPEQQHPKKAFGWAARDSSGVLSPFKFSRRETGKNDVRFKVLYCGICHTDLHMIKNEWGNSTYPLLPGHEIVG

VVTEVGTKVEKFKVGDHVGVGYMAGSCQSCDSCSTNAANYCPKMIATSGDQYHDGTPTYGGYSDIMVANEHFVVRI

PDSLPLDGAAPLLCAGVTTYSPLRYFGLDKPGMHVGVVGVGGLGHVALKFAKAMGLKVTMISTSPKKKEEAIENLG

ADSFLVSHDEGQMQGAVGTMDGIIDTVSAVHPLLPLVGLLKSNGKLVLLGVPDKPLELPVFPLLMGRKIIAGSVIG

GMKETQEMIDFAAKHNITANIEVISIDYVNTAMKRLVKADVKYRFVIDIRNTLHV

>PbCAD13

MAISPEEHPQKAFGWAARDSSGILYPFRFSRRENADDDVTIKVLYCGVCHSDLHFAKNDWGFTNYPVVPGHEIVGV

VTKTGNNVDKFKVGDRVGVGVIVGSCMKCETCQQDLENYCPRTIFTYNSHYHDQTKTYGGYSDIIVVHHRYVLRFP

DNLAPDAGAPLLCAGITVYSPMKYYGMTEPGKHLGVAGLGGLGHIAVKIGKAFGLKVTVISSSPAKEDEATRRLGA

DSFLLLSDPAKLKGGMGTMDYIIDTVSAVHALAPLIGLLKLNGKLVTVGLPNKPLELPIFPLVLGRKLVGGSDIGG

VKETQEMLDFCAKHNITSDIELIHMDYINTAMERLAKSDVRYRFVIDVASSLTQ

>PbCAD14

MAQTTPNHTQTVSGWAAHDPSGKVTPFVFKRRENGINDVTIKVLYCGICHTDLHFAKNDWGMTMYPVVPGHEITGL

ITKVGSNVKEFKEGDRVGVGCLAATCLECEFCKSSQENYCDQLQFTYNGIFWDGSITYGGYSKMLVADHRYVVHIP

ENLPLDATAPLLCAGVTVFTPMKDHKLHKTTGKKIGVVGLGGLGHVAVKFGKAFGHHVTVISTSPAKEKEAKEHLG

ADDFLVSTDPQQLQKGKRTLDFILNTVSAKHSLGSILELLKVNGTMVVVGAPDQPFELPSFPLIFGKRAVKGSVIG

GISETREMMNLCGQHNITCDIELTTPDKINEALDRVAKNDVRYRFVIDIAAADANARMPSKL

>PbCAD15

MPQEILVAEEQAVKAYGWAARDSSGILSPFHFIRRTTGSNDISMKILYCGICHSDLHLAKDEMGLTIYPIVPGHEI

VGEVTKVGSNVTRFQVGDIAGVGCMVGSCRSCDNCTQDLENYCPKIVWTYNKHQEDGSRTFGGYSDKLVVDQHFAV

RIPDNLPLQGTAPMLCAGITVYSPMRYFGLMEAGKHLGVVGLGGLGHMAVKFAKAVGAKVTVISTSPNKKKEAMEQ

LGVDEFLISHDQEQLQAAKGTMDGIIDTVSAPHPLLSLTEMLKTNGKLILVGAPAQPPELPVFSLMLGRKLIAGSA

IGGMKETQEMIDFAAKHNITADVEVIPMDYVNTALERVAKADIKYRFVIDVANTLNSPY

>PbCAD16

MSEEIPVAEEQAGKAYGWAARDSSGILSPFHFTRRTTGGNDISMKIIYCGICYSDLLFAKNEIGMTIYPIVPGHEI

VGEVTKVGCNVTKFKVGNIAGVGCMVGSCRSCDNCTQDFENYCSKIVWTYNEHYEDGSRTFGGYSDKLVVDEHFAV

RIPDNLSLQGTAPMLCAGITVYSPMKYFGLMEPGKHLGVVGLGGLGHMAVKFAKAVGAKVTVISTSPNKKKEAMEH

LGVDEFLISHDQEQLQADMGSMDGIIDTVSAPHSLSSLIELLKTNGKLILVGLIVQPPELPVFPLILEMLSDESLM

GRKLIAGSTIGGIKETQEMIDFAAKHNITADVEVIPMDYVNTALERVSKADVKYRFVIDVANTLNSPY

>PbCAD17

MVLTPEQEHPNKAFGWAARDSSGVLSPLKFSRRKTGEKDVRFKVLYCGICHSDLHMVKNEWGSSVYPLIPGHEIVG

VVTEVGSKVQKFNVGDKVGVGCMVGSCRSCDSCNNHLENYCPKMILTYGTRNLDGTITYGGYSDIMVADEHFIVRI

PDNLPLDGAAPLLCAGITTYSPLRYFGLDKPGMHVGVVGLGGLGHLAVKFAKAMGVKVTVISTSPNKKEEAIEHLH

ADSFLVSRDQDQMQAAMGTLDGIIDTVSALHPLVPLIGLLKSHGKLVMVGAPEKPLELPVFPLIMGRKIVGGSCIG

GMKETQEMIDFSAKHKITADIEVIPIDYLNTAMERLVKADVRYRFVIDIGNTLISSS

>PbCAD18

MAKSPEQEHPKKAFGWAARDSSGVLSPFKFSRRETGEKDVTFKVLYCGICHSDLHTIKSEWGITTYPLVPGHEIVG

TVTEVGNKVQKFKVGDKVGVGCLVGSCQSCDNCSKNLENYCPKMIQTYGSKYHDGTMTHGGYSDIMVADENFIVRI
PDKLPLDGAAPLLCAGITTYSPLRLHGLDKPGMHIGVVGLGGLGHAAVKFAKAFGVKVTVISTSPNKKEEAINRLK
ADSFLVSREQDQMQAAMGTMDGIIDTVSAVHALLPLLGLLKSNGKLVMVGLPEKPFELPIFPLVAGGKIVAGSCIG
GLKETQEMIDFAGKHNITADIEVIPIDYLNTAMERLAKADVRYRFVIDIGNTLKSAA

>PbCAD19

MSSDGANENCVGYAARDPSGVLSHYKFNRRALGADDVSIRITHCGVCYADVIWSRNKHGDSKYPLVPGHEIAGVVK
EVGSNCHRFKVGDHVGVGTYVNSCRDCEYCNEGLEVDCVKGSVYTFNAVDADGTITKGGYSSHIVVYERYCFKIPD
NYPLASAAPLLCAGITVYAPMKRHKMNQPGKSLGVIGLGGLGHMAVKFGKAFGLNVTVLSTSISKKEEALSQLGAD
KFVVSSDQEQMASLVKSFDFLIDTASGDHPFDPYMALLKTGGTLALVGFPSEVKFSPASLNLGRKAISGSVTGGTK
DTQEMIDFCAAQGIHPMIEIIPIQYATEALERLVKKDVKYRFVIDIESSLK

>PbCAD20

MSSQTSRVNGNCLGWAATDESGVLSPYKFTRRNLQNDDVSIRITHCGICYADVVFPRNKHGYANYPMVPGHEIAGI
VQEVGPNVHGFKVGDHVGVGTFVNSCRDCDYCRDGLEISCEKGIVFTYNHVDADGTITKGGFSSYIVAHERYCFKI
PEGYPLASAAPLLCAGITVYSPMIRHKMNQDGKSLGVIGLGGLGHLAVKFGKAFGLNVTVFSTSLSKKEEALSLLG
ADNFVVSSNQEEMTALAKSLDFIIDTASGDHPFDLYMSLLKTAGVLVLVGAPTEVKLSPGSLIGGMKSISGSAAGG
TKQTQEMLDFCAAHKIYPNIEIVPIQYVNEALERMIKRDVKYRFVIDIENSLK

>PbCAD21

MTIYPIVPGHEIVGEVTKVGCNVTKFKVGDIAGVGCMVGSCRSCNNCTQDLENYCPKMVWTYNKHHEDGSRTFGGY
SDKLVVDEHFAVQIPDNLLLQGTAPMLCAGITVYSPMRYFGLMEPGKHLGVVGLGGLGHMAVKFAKAVGAKVTVIS
TSPNKKKEAVEQLGVDEFLISHDQEQLQVAMGTMDGIIDTVSAPHPLLPLTELLKTNGKLILVGAPIQPPELPVFP
LILGRKLIAGSAIGGMKETQEMIDFAAKHNITADVEVIPMDYVHTAFERIAKADVKYRFVIDVANTLNSPY

>PbCAD22

MFKVLYCGICHLDLHMAKNEGGTSNNPLVPGHEIVGVVTEVRSKLQKFNVGDKVGVRCMVGSCKSCESCSDNLENY
CPKSILNYGSKYYDGTTIYGGYSDIMVANEHFVVCIPDNLPLDRAAPLLCVGITTYSPLRYFGLDKPGMHVGVVGL
GGLGHVAVKFAKAMVVKVTVISTSPNKKDEAIEHLHADSFLVSHNKRSNAATGTLDGIIDTVSALHPLLPLIGLLK
SHGKLVMVGAPEKPLELPIFPLLMGRKIVAGSATGGMKETQEMIDFVAKHNITVDIEVIPIDYVNIAMERLLKADV
RY

>PbCAD23

MKDHNLHTPTGKKIGVVGLGGLGHVAVKFGKAFGHQVTVISTSPAKEKEAKEHLGDDDFLVSTDPQQLQKGKRALN
FILNTVSAKHSLGPILELLKVMEVWWLWVHRTSHLNCLHFLLYLARKRAVKGSMIGGINEIKEMMDLCGKHKITCE
IELTMPDKINEALDCVAKNDFRYRFVIDIAADPNANAQISPNFRASEC

>PbCAD24

MFKVLYCGICHSDLHMAKNEGGTSNYPLVPRHEIVGVVTEVGSKVQKFNVGDKIGVGCMVGSCKSCESCSDNLENY
CPKSILTYGSKYYDGTTIYGGYFDIMVANEHFGVRIPLDRAAPLLCAGITTYSPLRYLGLDKPGMHVGVVGLGGLG
HVAVKFAKAMVVKVTVISTSPNKKDKAIEHLHADSHKIVGVVTEVGSKVQKFNVGDKIGVGCMVGSCKSCESCSDN
LENYCPKSILTYGSKYYDGTTIYGGYSDIMVANEHFVVRIPDNLPLDRTAHLLCAGITTYSPLRYFGLDKPGMHVG
VVGLGGLGHVAVKFAKAMVVKVTVISTSPNKKDESIEHLHTDSFLVSRNKRSDAATGTLDGIIDTISVLHPLLPLI
GLLKSHGKLVMVGAPEKPLELPIFPLLVGSKIVAGSATGGMKETQEMIDFVAKHNITVDIEVIPIDYVNIAIERLL
KADVRYRFVIDIGNTLKSTV

>PbCAD25

MNRETGAEDVMLKVLYCGVDHTDLHQMRNEIHSTNYPLVPGFVVRIPENLAPDQAAPLLCAGVTAYSPLKQFMGSN
RVLRAGILGLGGVGHLGVIIAKAMGHHVTVISSSDKKRQEALDILGADAFLVSSNAAEMEGAANSLDYILDTVPAF
HPLGSYLSLLTVDGKLIVVAAVPKPLQFDAVDLILGKKAIAGSFIGSMDETQEILEFWAEKGLKSFIEVVKMDYVN
KAFERMERNDVRFRFVLDVAGSNLE

>PbCAD26

MVANERYIVRFPENLPLDAGAPLPCAGITVYSPLKYFGLSEPGKHVGIVGLGGLGHTGVKFAKAFGSKVSVISTSP

GKKDEALKKLGADSFVVSCDQEQMKAARGTLDGIIGRVSAAHPIVPLLGLLKPHGKLILVGVPVKPLNLHVFPLIM

ESLADSGIGGMKETQEMIDFAAKHNITAEVEVISMDYVNTAMERLAKNDVRYRFVIDVANTLAAREA

>OsCAD1 (Os10g11810)

MAAECGSGNCDAWAARDPSGILSPYKFNRRAVQSDDVSLRITHCGVCYADVAWTRNILNNSMYPLVPGHEIAGVVT

EVGADVKSFKVGDHVGVGTYVNSCRDCENCNSSLENYCSQHVFTFNGVDTDGTVTKGGYSTHIVVHERYCFKIPDG

YPLEKAAPLLCAGITVYSPMMRHNMNQPGKSLGVIGLGGLGHMAVKFGKAFGLKVTVISTSESKRKEAIDLLGADN

FVVSSDENQMETLKSSLNFIIDTASGDHPFDPYLTLLKVGGVMALLSFPSEIKVHPANLNLGGRSLSGSVTGGTKD

IQEMINFCAANKIYPDIEMIKIDYINEALQRLVDRDVRFRFVIDIENSFK

>OsCAD2(Os02g09490)

MGSLAAEKTVTGWAARDASGHLTPYNYTLRKTGPEDVVVKVLYCGICHTDIHQAKNHLGASKYPMVPGHEVVGEVV

EVGPEVTKYSAGDVVGVGVIVGCCRECHPCKANVEQYCNKRIWSYNDVYTDGRPTQGGFASAMVVDQKFVVKIPAG

LAPEQAAPLLCAGLTVYSPLKHFGLMSPGLRGGVLGLGGVGHMGVKVAKSMGHHVTVISSSARKRGEAMDDLGADA

YLVSSDAAAMAAAGDSLDYIIDTVPVHHPLEPYLALLKLDGKLILMGVINQPLSFISPMVMLGRKAITGSFIGSMA

ETEEVLNFCVDKGLTSQIEVVKMDYVNQALERLERNDVRYRFVVDVAGSNIDDADAPPA

>OsCAD3(Os10g29470)

MAPTAASAAAGEEQQAVGLAARDSSGHLSPFAISRRSTGDDDVAIKILFCGICHSDLHCIKNEWKHSIYPLVPGHE

IAGVVTEVGKNVTRFKAGDRVGVGCMVNSCRSCESCNNGFENHCPEGVFTYNSVDKDGTVTYGGYSSMVVVHERFV

VMFPEAMPLDVGAPLLCAGITVYTPMKYHGLNAPGKHVGVLGLGGLGHVAVKFARAFGLKVTVISSSPGKKREALE

RLGADAFVVSSSAEEMEAARSTMDGVINTVSANTPMAPYLALLKPNGKMILVGLPENPLEVPPFSLVHGNRTLAGS

NIGGMADTQEMIELAAKHGVTADIEVIGADDVNTAMERLAKADVRYRFVIDVGNTLHAAAAE

>OsCAD4(Os11g40690)

MAAECGSGNCDAWAARDPSGILSPYKFNRREVQSEDVSLRITHCGVCYADVIWTRNMFNDSIYPLVPGHEIAGVVT

EVGADVKGFKVGDHDCENCNSSLENHCSKCVVTYNSVDSDGTVTKGGYSSHILVHQRYCFKIPADYPLSKAAPLLC

AGITVYTPMIRHNMNQPGKSLGVIGLGGLGHMAVKFGKAFGLKVTVFSTSESKREEAINLLGADNFVISSDENQME

SLKSSLHFIIDTASGDHQFDPYLSLLKVGGVMVLLSFPSEIKVHPENLNLAARSLAGSVTGGTKDIQEMINFCAAN

NVYPDIEMIKIDYVNEALQRLINRDVRFRFVIDIENSFK

>OsCAD5(Os08g16910)

MAPTAAAGLAARDASGHLSPLTISRRSTGDDDVVIKILYCGICHSDLHSIKNEWKNATYPLVPGHEIAGVVTEAGK

NVTKFKGGDKVGVGCMVNSCHSCDSCNQGLENHCPGVIFTYNSVDKDGTVTYGGYSSMVVVHERFVVRFPEAMPLD

KGAPLLCAGITVYSPMKYHGLNVPSKHVGVLGLGGLGHVAVKFAKAFGMTVTVISSSPGKRQEALERLGADAFVVS

KNADEMNAATGTMDGIINTVSANIPIAPLLGLLKPNGKMILVGLPEKPMEIPPFALVASKHGVTAEIEVIGADYVN

TAMERLAKADVRYRFVIDIGNTLKDAIE

>OsCAD6(Os04g15920)

MEVTPNHTQTVSGWAAMDESGKIVPFVFKRRENGVDDVTIKVKYCGMCHTDLHFIHNDWGITMYPVVPGHEITGVV

TKVGTNVAGFKVGDRVGVGCIAASCLDCEHCRRSEENYCDKVALTYNGIFWDGSITYGGYSGMLVAHKRFVVRIPD

TLPLDAAAPLLCAGITVYSPMKQHGMLQADAAGRRLGVVGLGGLGHVAVKFGKAFGLHVTVISTSPAKEREARENL

KADNFVVSTDQKQMQAMTRSLDYIIDTVAATHSLGPILELLKVNGKLVLVGAPEKPVELPSFPLIFGKRTVSGSMT

GGMKETQEMMDICGEHNITCDIEIVSTDRINDALARLARNDVRYRFVINVGGDSKL

>OsCAD7(Os04g52280)

MAPTTTATAAAEQAPPPQHTRKAVGLAAHDDSGHLTPIRISRRKTGDDDVAIKVLYCGICHSDLHTIKNEWRNAVY

PVVAGHEITGVVTEVGKNVARFKAGDEVGVGCMVNTCGGCESCRDGCENYCSGGVVFTYNSVDRDGTRTYGGYSDA

VVVSQRFVVRFPSSAGGGAGAALPLDSGAPLLCAGVTVYAPMRQHGLCEAGKHVGVVGLGGLGHVAVKFARAFGMR

Biology Open • Supplementary information

VTVISTSPVKRQEALERLGADGFIVSTNASEMKAAMGTMHGIINTASASTSMHSYLALLKPKGKMILVGLPEKPLQ
IPTFALVGGGKILAGSCMGSISETQEMIDFAAEHGVAADIELIGADEVNTAMERLAKGDVRYRFVVDIGNTLRSD
>OsCAD8(Os09g23530)
MEHDGTAALGWAARDASGHLSPFSFTRRVQEEDDVTIKVLYCGICHTDLHTIKNEWGNAMYPVVPGHEIVGVVAGV
GAGVTRFKAGDTVGVGYFVDSCRACDSCGKGDENYCPTMVITSNGTDYGGATTQGGFSDVMVVRQDYVLRVPASLP
PDGAAPLLCAGVTVYSPMVEYGLNAPGKHLGVVGLGGLGHLGVKFGKAFGMKVTVISSSPAKREEALERLGADAFL
SSRDGEGMAAAAATMDGIIDTVSAGHPLVPLLSLLKPKGQMVVVGAPAAPLQLPAIAIIDGGKRVAGSGGGSVAEC
QAMLDFAGEHGIAADVEVVAMGDVNAALGRLERNDVRYRFVIDVAGTLHAAAAPS
>OsCAD9(Os09g23540)
MSRHFRTHTTSRLTFPSSSGGLAITRLPFSSTSSKLLLQQLSSTSPAAAATAVTITTSSPARNLQRARASAAEQGM
EEHGKAAVGWAARDDSGVLSPYNFSRRAQKDDDVTIKVLYCGICHTDLHVVKNDWGNAMYPVVPGHEIVGVVTGVG
AGVTKFKAGDTVGVGFFVGSCRTCDSCGKGYENYCPTMVITSNGKDYGGAATQGGFSDAIVVNEHYVLRVPAGLPL
DGAAPLLCAGVTVYSPMVIHGLNAPGKHVGVVGLGGLGHVAVKFAKAFGMRVTVISTSPGKRREALEHLGADEFLV
SRDAGQMAAAAGTMDGILNTVSAWHPVAPLFALMKPMAQMVFVGAPTRPLELPAYAIVPGGKGITGNCVGGIRDCQ
AMLDFAGEHGITAEVEVIKMDYVNTAMERLEKNDVRYRFVIDVAGSSLGGSGDDKI
>OsCAD10(Os09g23550)
MSRHFRTHTTSRLTFPSSSGGLAITRLPFSSTSSKLLLQQLSSTSPAAAATAVTITTSSPARNLQRARASAAEQGM
EEHGKAAVGWAARDDSGVLSPYNFSRRAQKDDDVTIKVLYCGICHTDLHIVKNDWGNAMYPVVPGHEIVGVVTGVG
AGVTKFKAGDTVGVGYFVASCRGCECCGNGYENYCAKMVTTCNGVDHDHGGGAATQGGFSDAIVVNEHYVLRVPAG
LPLDSAAPLLCAGVTVYSPMVIHGLNAPGKHVGVVGLGGLGHVAVKFAKAFGMRVTVISTSPGKRQEALEHLGADE
FLVSRDAGQMAAAAATMDGILNTVSAWHPIAPLFSLMKPMAQMVFVGGPTRPLELPAYAIVPGGKGITGNCVGGIR
DCQAMLDFAGEHGITAEVEVIKMDYVNTAMERLEKNDVRYRFVIDVAGSSLAGSGDAKI
>OsCAD11(Os09g23560)
MEHNGTAALGWAARDTSGHLSPFSFTRRVQQEDDVTIKVLYCGICHTDLHIIKNEWGNAMYPVVPGHEIVGVVTGV
GAGVTKFKAGDTVGVGYFVDSCRACDSCGKGYENYCPTMVITSNGTDYGGATTQGGFSDVMVVRQDYVVRVPASLP
PDGAAPLLCAGVTVYSPMVEYGLNGPGKHLGVVGLGGLGHLGVKFGKAFGMKVTVISSSPAKRGEALGRLGADAFL
SSRDGEGMAAAAATMDGIIDTVSAGHPLVPLLSLLKPKGQMVVVGAPAMPLQLPAYAIIEGGKRVAGNGVGSVAEC
QAMLDFAGEHGIAADVEVVAMDAVNAALGRLERNDVRYRFVVDVAGTMHAAAAAA
SS
>OsCAD12(Os03g12270)
MEQQPKMVTGWAARDANGLLSPFSYPLRAKGDEDVVVKILFCGICHSDLSTIKNEWGNAKYPVVPGHEIVGVVAEV
GSSVARFAAGDTVGVGYIASTCRACANCRDGFENYCAGLVPSFNAALPDGATVHGGFSELAVVNQRYVVRIPGGGG
GASPAPLDRLAPLLCAGVTVYCPMRRLGLDRPGVHLGVAGLGGLGHLAVKFGKAFGVKVTVISTSPWKEAEAVERL
GADAFLLSTNAEQMKAAAGTMDGIIDTVSAVHDLTPLITLLRTHGQLVPVGSPGKPVQLALYPLQSDGKSVAGSMI
GGMRDTQEMVDFAVEHGVAAEVEVIGMEDVNGAMERLQKGDVRYRFVIDVANTMARAR
>AtCAD1(AY288079)
MSSSESVENECMCWAARDPSGLLSPHTITRRSVTTDDVSLTITHCGVCYADVIWSRNQHGDSKYPLVPGHEIAGIV
TKVGPNVQRFKVGDHVGVGTYVNSCRECEYCNEGQEVNCAKGVFTFNGIDHDGSVTKGGYSSHIVVHERYCYKIPV
DYPLESAAPLLCAGITVYAPMMRHNMNQPGKSLGVIGLGGLGHMAVKFGKAFGLSVTVFSTSISKKEEALNLLGAE
NFVISSDHDQMKALEKSLDFLVDTASGDHAFDPYMSLLKIAGTYVLVGFPSEIKISPANLNLGMRMLAGSVTGGTK
ITQQMLDFCAAHKIYPNIEVIPIQKINEALERVVKKDIKYRFVIDIKNSLK
>AtCAD2(AY302077)
MVDQNKAFGWAANDESGVLSPFHFSRRENGENDVTVKILFCGVCHSDLHTIKNHWGFSRYPIIPGHEIVGIATKVG
KNVTKFKEGDRVGVGVIIGSCQSCESCNQDLENYCPKVVFTYNSRSSDGTSRNQGGYSDVIVVDHRFVLSIPDGLP

Biology Open • Supplementary information

SDSGAPLLCAGITVYSPMKYYGMTKESGKRLGVNGLGGLGHIAVKIGKAFGLRVTVISRSSEKEREAIDRLGADSF
LVTTDSQKMKEAVGTMDFIIDTVSAEHALLPLFSLLKVNGKLVALGLPEKPLDLPIFSLVLGRKMVGGSQIGGMKE
TQEMLEFCAKHKIVSDIELIKMSDINSAMDRLAKSDVRYRFVIDVANSLLPESSAEILTEQVDHGVSITSRF
>AtCAD3(AY302078)
MVDQNRAFGWAANDESGVLSPFHFSRRENGENDVTVKILFCGVCHSDLHTIKNHWGFSRYPIIPGHEIVGIATKVG
KNVTKFKEGDRVGVGVIIGSCQSCESCNQDLENYCPKVVFTYNSRSSDGTRNQGGYSDVIVVDHRFVLSIPDGLPS
DSGAPLLCAGITVYSPMKYYGMTKESGKRLGVNGLGGLGHIAVKIGKAFGLRVTVISRSSEKEREAIDRLGADSFL
VTTDSQKMKEAVGTMDFIIDTVSAEHALLPLFSLLKVSGKLVALGLLEKPLDLPIFPLVLGRKMVGGSQIGGMKET
QEMLEFCAKHKIVSDIELIKMSDINSAMDRLVKSDVRYRFVIDVANSLLPESSAEILTEHVDHGVSITSRF
>AtCAD4(AY302081)
MGSVEAGEKKALGWAARDPSGVLSPYSYTLRSTGADDVYIKVICCGICHTDIHQIKNDLGMSNYPMVPGHEVVGEV
LEVGSDVSKFTVGDVVGVGVVVGCCGSCKPCSSELEQYCNKRIWSYNDVYTDGKPTQGGFADTMIVNQKFVVKIPE
GMAVEQAAPLLCAGVTVYSPLSHFGLMASGLKGGILGLGGVGHMGVKIAKAMGHHVTVISSSDKKKEEAIEHLGAD
DYVVSSDPAEMQRLADSLDYIIDTVPVFHPLDPYLACLKLDGKLILMGVINTPLQFVTPLVILGRKVISGSFIGSI
KETEEVLAFCKEKGLTSTIETVKIDELNIAFERLRKNDVRYRFVVDVAGSNLVEEAATTTN
>AtCAD5(AY302082)
MGIMEAERKTTGWAARDPSGILSPYTYTLRETGPEDVNIRIICCGICHTDLHQTKNDLGMSNYPMVPGHEVVGEVV
EVGSDVSKFTVGDIVGVGCLVGCCGGCSPCERDLEQYCPKKIWSYNDVYINGQPTQGGFAKATVVHQKFVVKIPEG
MAVEQAAPLLCAGVTVYSPLSHFGLKQPGLRGGILGLGGVGHMGVKIAKAMGHHVTVISSSNKKREEALQDLGADD
YVIGSDQAKMSELADSLDYVIDTVPVHHALEPYLSLLKLDGKLILMGVINNPLQFLTPLLMLGRKVITGSFIGSMK
ETEEMLEFCKEKGLSSIIEVVKMDYVNTAFERLEKNDVRYRFVVDVEGSNLDA
>AtCAD6(AY302075)
MERLSGEKEQSVEAFGWAARDSSGHLSPFVFSRRKTGEEEVRVKVLYCGICHSDLHCLKNEWHSSIYPLVPGHEII
GEVSEIGNKVSKFNLGDKVGVGCIVDSCRTCESCREDQENYCTKAIATYNGVHHDGTINYGGYSDHIVVDERYAVK
IPHTLPLVSAAPLLCAGISMYSPMKYFGLTGPDKHVGIVGLGGLGHIGVRFAKAFGTKVTVVSSTTGKSKDALDTL
GADGFLVSTDEDQMKAAMGTMDGIIDTVSASHSISPLIGLLKSNGKLVLLGATEKPFDISAFSLILGRKSIAGSGI
GGMQETQEMIDFAAEHGIKAEIEIISMDYVNTAMDRLAKGDVRYRFVIDISNTLAATRS
>AtCAD7(AY302079)
MGKVLEKEAFGLAAKDESGILSPFSFSRRATGEKDVRFKVLFCGICHTDLSMAKNEWGLTTYPLVPGHEIVGVVTE
VGAKVKKFNAGDKVGVGYMAGSCRSCDSCNDGDENYCPKMILTSGAKNFDDTMTHGGYSDHMVCAEDFIIRIPDNL
PLDGAAPLLCAGVTVYSPMKYHGLDKPGMHIGVVGLGGLGHVAVKFAKAMGTKVTVISTSERKRDEAVTRLGADAF
LVSRDPKQMKDAMGTMDGIIDTVSATHPLLPLLGLLKNKGKLVMVGAPAEPLELPVFPLIFGRKMVVGSMVGGIKE
TQEMVDLAGKHNITADIELISADYVNTAMERLAKADVKYRFVIDVANTMKPTP
>AtCAD8(AY302080)
MGKVLQKEAFGLAAKDNSGVLSPFSFTRRETGEKDVRFKVLFCGICHSDLHMVKNEWGMSTYPLVPGHEIVGVVTE
VGAKVTKFKTGEKVGVGCLVSSCGSCDSCTEGMENYCPKSIQTYGFPYYDNTITYGGYSDHMVCEEGFVIRIPDNL
PLDAAAPLLCAGITVYSPMKYHGLDKPGMHIGVVGLGGLGHVGVKFAKAMGTKVTVISTSEKKRDEAINRLGADAF
LVSRDPKQIKDAMGTMDGIIDTVSATHSLLPLLGLLKHKGKLVMVGAPEKPLELPVMPLIFERKMVMGSMIGGIKE
TQEMIDMAGKHNITADIELISADYVNTAMERLEKADVRYRFVIDVANTLKPNPNL
>AtCAD9(AY302076)
MAKSPETEHPNKVFGWGARDKSGVLSPFHFSRRDNGENDVTVKILFCGVCHTDLHTIKNDWGYSYYPVVPGHEIVG
IATKVGKNVTKFKEGDRVGVGVISGSCQSCESCDQDLENYCPQMSFTYNAIGSDGTKNYGGYSENIVVDQRFVLRF
PENLPSDSGAPLLCAGITVYSPMKYYGMTEAGKHLGVAGLGGLGHVAVKIGKAFGLKVTVISSSSTKAEEAINHLG
ADSFLVTTDPQKMKAAIGTMDYIIDTISAVHALYPLLGLLKVNGKLIALGLPEKPLELPMFPLVLGRKMVGGSDVG

Biology Open • Supplementary information

GMKETQEMLDFCAKHNITADIELIKMDEINTAMERLAKSDVRYRFVIDVANSLSPP
>PtoCAD1(KF145200)
MGSLETERKIVGWAATDSTGHLAPYTYSLRDTGPEDVFIKVISCGICHTDIHQIKNDLGMSHYPMVPGHEVVGEVV
EVGSDVTKFKAGDVVGVGVIVGSCKNCHPCKSELEQYCNKKIWSYNDVYTDGKPTQGGFAESMVVDQKFVVRIPDG
MSPEQAAPLLCAGLTVYSPLKHFGLKQSGLRGGILGLGGVGHMGVKVAKAMGHHVTVISSSDKKREEAMEHLGADE
YLVSSDVESMQKAADQLDYIIDTVPVVHPLEPYLSLLKLDGKLILMGVINTPLQFVSPMVMLGRKSITGSFIGSMK
ETEEMLEFCKEKGLASMIEVIKMDYINTAFERLEKNDVRYRFVVDVAGSKLIP
>PtoCAD2(KF145201)
MSKSPEEEHPVKAFGWAARDQSGHLSPFNFSRRATGEEDVRFKVLYCGICHSDLHSIKNDWGFSMYPLVPGHEIVG
EVTEVGSKVKKVNVGDKVGVGCLVGACHSCESCANDLENYCPKMILTYASIYHDGTITYGGYSDHMVANERYIIRF
PDNMPLDGGAPLLCAGITVYSPLKYFGLDEPGKHIGIVGLGGLGHVAVKFAKAFGSKVTVISTSPSKKEEALKNLG
ADSFLVSRDQEQMQAAAGTLDGIIDTVSAVHPLLPLFGLLKSHGKLILVGAPEKPLELPAFSLIAGRKIVAGSGIG
GMKETQEMIDFAAKHNITADIEVISTDYLNTAMERLAKNDVRYRFVIDVGNTLAATKP
>PtoCAD3(KF145202)
MAQTTPNHTQTVSGWAALDSSGKVVPYTFKRRENGVNDVTIEIMYCGICHTDLHFAKNDWGISMYPVVPGHEITGI
ITKVGSNVNKFRLGDRVGVGCLAASCLECDFCKSSHENYCDQIQLTYNGIFWDGSITYGGYSKFLVADHRYVVRIP
ENLPMDATAPLLCAGITVFSPFKDSNLVDTPGKRVGVVGLGGLGHVAVKFGKAFGHHVTVISTSPSKEKEARERLG
ADDFIVSTNAQELQAARRTLDFIVDTVSAKHSLGPVLELLKVNGTLAVVCAPDQPMELPAFPLIFGKRSVRGSMTG
STSETQEMLDVCGKHNITCDIELVKTDNINEAWDRLARNDVRYRFVIDIAGKSSNL
>PtoCAD5(KF145203)
MVAKLPEEEHPKQAFGWAARDQSGLLSPFKFSRRATAEKDVAFKVLYCGMCHSDLHMAKNEWGVTQYPLVPGHEIV
GIVTEVGSKVEKFKVGDKVGVGCMVGSCRSCDSCHDNLENYCPKMILTYGAKYYDGTTTYGGYSDLMVAEEHFIVR
IPDNLSLEAGAPLLCAGITVYSPLRYFGLDKPGMHVGVVGLGGLGHVAVKFAKAMGVKVTVISTSPNKKQEAVEIL
GADSFLVSRDQDQMQSAMGTLDGIIDTVSAVHPMVPLFTLLKSHGKLVLVGAPEKPLELPAFPLIMGRKMVGGSCI
GGMTETQEMIDFAAKHNITADVEVIPMDYVNTAMERILKGDVRYRFVIDIAKPLNP
>PtoCAD6(KF145199)
MAEKSYEEEHPTRAFGWAARDQSGVLSPFKFSRRSTGEKDVRFKVLYCGICHSDLHMAKNEWGTSMYPLVPGHEIV
GEVTEVGSKVEKFKVGDKVGVGCMVGSCHSCDSCNSDLENYCPKMILTYSTKYHDGTTTYGGYSDSMVTDEHFVVR
IPDNLPLDAAAPLLCAGITVYSPLRFFNLDKPGMHVGVVGLGGLGHVAVKFAKAMGVKVTVISTSPKKKQEALEHL
GADSFLVSRDQDEMQAAMGTMDGVIDTVSAMHPILPLISLLKTQGKLVLVGAPEKPLELPVFPLIMGRKIVGGSCI
GGMKETQEMIDFAAKNNITADIEVISMDYVNTAMERLLKTDVRYRFVIDIGNTMKI
>PtoCAD7(KJ159966)
MADKLPEEEHPKPAFGWAARDQSGLLSPFKFSRRATGEKDVAFKVLYCGICHSDLHMVKNEWGATQYPLIPGHEIV
GVVTEVGSEVEKFKVGDKVGVGCMVGSCRTCDSCQNDLENYCSKKILTYGARYYDGTVTYGGYSDSMVADEHFIVR
IPDNLPPDAGAPLLCAGITVYSPLRYFGLDKPGMHVGIVGLGGLGHVAVKFARAMGVKVTVISTSPDKKQEALENL
GADSFLVSRDQDQMQAAMGTLDGIIDTVSAVHPLLPLVALLKSHGKLVLVGAPEKPLELPVSPLIIGRKTVGGSCF
GGIKETQEMIDFAAEHKITADIEVIPMDYVNTAMERVLKTDVRYRFVIDVGKTLKPDV
>PtoCAD8(KF145195)
MAAKSCQDGHPAEAFGWAARDHSGVLSPFKFSRRATGEKDVAIKVLFCGICHSDLHMIKNEWGISSYPVVPGHEIV
GQVTGVGSKVEKFKVGDKVGVGYMVGSCQSCDSCHDDLENYCPETIVTSGGKYHDGATTYGGFSDIMVADEHYVIR
IPENLPLDAGAPLLCAGITVYSPLKYYGLDKPGMHVGVVGLGGLGHVAVKFAKAMGIKVTVISTSPKKKQEALEHL
GAHSFLVSRDPDQMQAAMGTMDGIIDTVSTMHPLFPLIGLLKTQGKLVLVGAPEKPLELPVFPLITGRKIVGGSSI
GGIKETQEMIDFAAKNDITADVEVIPMDYVNTALERLSKSDVRYRFVIDIGNTLKI
>PtoCAD9(KF145196)

Biology Open • Supplementary information

MAKSPEEEHPHKAFGWAAKDSSGVLSPFHFTRRDNGVEDVTIKILYCGVCHSDLHAAKNEWGFSRYPLVPGHEIVG
IVTKIGSNVKKFKVDDQVGVGVMVNSCKSCEYCDQDSENYCPKMIFTYNAQNYDGTKTYGGYSDTIVVDHHFVLRI
PDSMPADGAAPLLCAGITVYSPMKYYGMTEPGKHLGIVGLGGLGHVAVKIGKAFGLKVTVISSSSRKEREALDRLG
ADSFLVSSDPEKMKAAFGTMDYIIDTVSAVHALAPLLSLLKTNGKLVTLGLPEKPLELPIFPLVLGRKLVGGSDIG
GMKETQEMLDFCAKHNITSDVEVIRMDQINTAMERLAKSDVRYRFVTDVANSLSQSQL
>PtoCAD12(KJ159967)
MSSEGVKDDCLAWAAGDPSGVLSPYKFSRRALGKDDVSLKITHCGVCYADVIWSKNKHGDSRYPLVPGHEIAGIVK
EIGSSVSNFKVGDHVGVGTYVNSCRECEYCNDKEEVSCEKGSVFTFNGIDADGSITKGGYSSYIVVHERYCFRIPD
GYPLASAAPLLCAGITVYNPMMRHKMNQPGKSLGVIGLGGLGHMAVKFGKAFGLKVTVLSTSVSKKEEALSVLGAD
NFVITSDQAQMKALYKSLDFIIDTASGDHPFDPYLSLLKTAGVFVLVGFPSEVKFSPASLNIGMKTVAGSITGGTR
VIQRCGLLCC
>ZmCAD1(AJ005702)
MGSASRKVVGWAARDATGHSYSYTRNTGDVVVKVYCGICHTDIHAKNHGASKYMVGHVVGVVVGVAKYGVGDVVGV
GVIVGCCRCSCKANVYCNKKIWSYNDVYTDGRTGGASTMVVDKVVKIAGAAACAGVTVYSKHGTTGRGGIGGGVGH
MGVKVAKAMGHHVTVISSSSKKRAAMDHGADAYVSSDAAAMGAADSDYIIDTVVHHYAKDGKVGVIGSVSMVMGRK
AITGSIGSIDTAVCVDKGTSIVVKMGYVNARRNDVRYRVVDVAGSNVAAAAADAASN
>TaCAD1(GU563724)
MGSVDASTTVTGWAARDATGHSYRYTRKTGDVVKVKYCGICHTDVHVKNDGASKYMVGHVVGVVVGVSKRAGDVVG
VGVIVGCCRDCRCKANVYCNKKIWSYNDVYTDGKTGGASAMVVDKVVKIAGAAACAGVTVYSKHGMTGRGGIGGGV
GHMGVKVAKSMGHHVTVISSSNKKRAAMDDGADAYVSSDTDMAAAADSDYIIDTVAKHYAKMDGKVMGVIASVSMV
MGRKTITGSIGSMDTVCVDKGTSIVVKMDYVNARRNDVRYRVVDVGGSNIDAA
>CsCAD3(HQ880208)
MAKAHVAGWAARDTSGVSKSRRATGTDVTKVYSGMCHSDHTIKNWGNTNYSGHIVGVVTVGSKVKKVGDKVGVGCM
VGSCHSCDNCANDNYCKMTYNSIYYDGTTYGGYSNIMVADHVIRIDSDASACAGITTYSKHGDGMHVGVVGGGGHA
AVKAKAGVKVTVISTSNKKKAIRGADSDSRDTMVVMGTMDGIIDTVSAHIGKSHGKIVGAKVMGRKIVAGSCIGGI
KTMIDAAKHNITADIVIMDYVNTARVKADVRYRVIDIGNTKIA
>PtSAD(AF273256)
MSKSPEEEHPVKAFGWAARDQSGHLSPFNFSRRATGEEDVRFKVLYCGVCHSDLHSIKNDWGFSMYPLVPGHEIVG
EVTEVGSKVKKVNVGDKVGVGCLVGACHSCESCANDLENYCPKMILTYASIYHDGTITYGGYSDHMVANERYIIRF
PDNMPLDGGAPLLCAGITVYSPLKYFGLDEPGKHIGIVGLGGLGHVAVKFAKAFGSKVTVISTSPSKKEEALKNFG
ADSFLVSRDQEQMQAAAGTLDGIIDTVSAVHPLLPLFGLLKSHGKLILVGAPEKPLELPAFSLIAGRKIVAGSGIG
GMKETQEMIDFAAKHNITADIEVISTDYLNTAMERLAKNDVRYRFVIDVGNTLAATKP
>BdCAD5(XP_003570974.1)
MGSIASEERTVTGWAARDADGHLSPYTYTLRKTGPEDVLVKVLYCGICHTDIHQAKNHLGASKYPMVPGHEVVGEV
VEVGGEVSKYSAGDVVGVGVIVGCCRECRPCKASVEQYCNKRIWSYNDVYTDGRPTQGGFSSAMVVDQKFVVRIPA
GLAPEQAAPLLCAGVTVYSPLKHFGLMSPGLRGGILGLGGVGHMGVKVAKSMGHHVTVISSSGKKRAEAMDDLGAD
AYLVSSDADQMAAAADSLDYVIDTVPVKHPLEPYLSLLKMDGKLVLMGVIGEPLSFVSPMVMLGRKSITGSFIGSM
EETEEVLKFCVEKGLTSQIEVVKMDYLNEALERLERNDVRYRFVVDVAGSKIEDAA
>GmCAD1(HQ651807)
MAKSTVAGWAASDTSGTAHSRRNGVDDVTKICGVCHSDHTKNDWGTTYVVGHIVGVVTKVGNNVKNKVGDKVGVGV
IVSCKCSCDSYCRVTYNSYYDGTRTKGGYSNIMVVHRYVRNDAGACAGITVYSMKYYGMTGKHGVAGGGGHVAIKA
KAGKVTVISSSNKAAIDRGADVSSDAKMKAAGTMDYIIDTISAVHSIGKNGKVTVGNKIVAGRKIGGSNGGKTMDC
GKHNITADIIKMDINTAMRSRADVKYRVIDVASSSS
>ShCAD1(L36823)

MASSKAGWAAKDASGHSHTRRNADDVTKIYCGVCHSDHTVKNDWGTTYVVGHIAGIVTKVGSNVTKKGDRVGVGVI
VDSCCCCDSYCKVTYNSYKGTRTGGYSDVVVHRVDNDAGACAGITVYSMKYYGMTGKHGVAGGGGHVAIKGKAGKV
TVISSSNKSAIDVGADSSSDKMKAATGTMDYIIDTISAVHSVSGKNGKVTVGSKIVAGRKIGGSNGGKTMDCGKHN
IAANIIKMDINTAIRSKADVKYRVIDVANSSSSNM

>ShCAD3(L36456)

MASVHRKAGWAARDSSGSNSRRDIGDVAVYCGICHTDHMAKNDGNSIYYVGHVIGIVAVGSKVKYKVGDKVGVGYV
SCRSCNCIDNNYCKHITGDKHIDGTTTYGGYSDSMVVDHVTRIGDGCGSSSCWGYSHSKYYGDKGHVGVVGGGGHM
VAKAKTHGKITVISTSTKKAIKNGADSVSRDDMAKTDGIIDTVSADHSIVIGKSHGKVIGAIKIGRKVGGTVGGKT
MIDSKHNVKIVVMDYVNIAMRAKADVKYRVIDVANTKTS

>MtCAD1(KEH44099)

MGSIEVAERTTVGLAAKDPSGILTPYTYTLRNTGPDDVYIKIHYCGVCHSDLHQIKNDLGMSNYPMVPGHEVVGEV
LEVGSNVTRFKVGEIVGVGLLVGCCKSCRACDSEIEQYCNKKIWSYNDVYTDGKITQGGFAESTVVEQKFVVKIPE
GLAPEQVAPLLCAGVTVYSPLSHFGLKTPGLRGGILGLGGVGHMGVKVAKAFGHHVTVISSSDKKKKEALEDLGAD
SYLVSSDTVGMQEAADSLDYIIDTVPVGHPLEPYLSLLKIDGKLILMGVINTPLQFVTPMVMLGRKSITGSFVGSV
KETEEMLEFWKEKGLSSMIEIVTMDYINKAFERLEKNDVRYRFVVD
VKGSKFED

>NtCAD(CAA44217)

MGSLDVEKSAIGWAARDPSGLLSPYTYTLRNTGPEDVQVKVLYCGLCHSDLHQVKNDLGMSNYPLVPGHEVVGKVV
EVGADVSKFKVGDTVGVGLLVGSCRNCGPCKREIEQYCNKKIWNCNDVYTDGKPTQGGFANSMVVDQNFVVKIPEG
MAPEQAAPLLCAGITVYSPFNHFGFNQSGFRGGILGLGGVGHMGVKIAKAMGHHVTVISSSNKKRQEALEHLGADD
YLVSSDTDKMQEAADSLDYIIDTVPVGHPLELYLSLLKIDGKLILIGVINTPLQFISPMVMLGRKSITGSFIGSMK
ETEEMLDFCKEKGVTSQIEIVKMDYINTAMERLEKNDVSYRFVVDV
AGSKLDQ

>Bmr6(Sb04g005950)

MGSLASERKVVGWAARDATGHLSPYTYTLRNTGPEDVVVKVLYCGICHTDIHQAKNHLGASKYPMVPGHEVVGEVV
EVGPEVSKYGVGDVVGVGVIVGCCRECSPCKANVEQYCNKKIWSYNDVYTDGRPTQGGFASTMVVDQKFVVKIPAG
LAPEQAAPLLCAGVTVYSPLKAFGLTAPGLRGGIVGLGGVGHMGVKVAKAMGHHVTVISSSSKKRAEAMDHLGADA
YLVSTDAAAMAAAADSLDYIIDTVPVHHPLEPYLSLLRLDGKHVLLGVIGEPLSFVSPMVMLGRKAITGSFIGSID
ETAEVLQFCVDKGLTSQIEVVKMGYVNEALERLERNDVRYRFVVDVAGSNVEEDAADAPSN

>PviCAD1(GU045611)

MGSLASERTVVGWAARDAAGHLSPYTYTVRNTGPEDVVVKVLYCGICHTDIHQAKNHLGASKYPMVPGHEVVGEVV
EVGPEVSKHRVGDVVGVGVIVGCCRECRPCKANVEQYCNKRIWSYNDVYTDGRPTQGGFASTMVVDQKFVVPIPAG
LAPEQAAPLLCAGVTVYSPLKHFGLTAPGLRGGILGLGGVGHMGVKVAKALGHHVTVISSSSRKRAEAMDDLGADA
YLVSSDAGAMAAAADSLDYIIDTVPVHHPLEPYLALLRLDGKHVLLGVVGEPLSFVSPMVMLGRKSVTGSFIGSVD
ETAELLRFCVDKGLTSQIEVVKMGYVNEALERLERNDVRYRFVVDVAGSNIEEQAAAAGAPAN

Biology Open • Supplementary information

Table S12 Primers for qRT-PCR and gene cloning.

Name	Primer sequence (5'-3')	Application
q-PbCAD2	F: AAGGAAACTGAGGAGATGCTTGAAT<br>R: TACTTTATTAAATAAGATTGCTGCCG	qRT-PCR
q-PbCAD3	F: AGAGGATGGACAACTATGATCGAAG<br>R: GAATTCAGTCTTACATGAACGTGTT	
q-PbCAD1	F: CAAGAAAAAAAGACGGCCCTGGCTA<br>R: TTCGAAGCGAAATATCAATTTATGT	
q-PbCAD7	F: ATTTCTGGTAGCGGAACGGGAGGTA<br>R: TCACACCCAGTGAATTTCCGGCCTG	
q-PbCAD9	F: TATGAAATCGATATCTGGTAGCGTG<br>R: CTTGTTCAATATTTCAGGGAGTTCT	
q-PbCAD11	F: TGCGGAGGTTGAAGTTATCTCAATG<br>R: TTTCCCTTCTTGAGTCTTCAATCTT	
q-PbCAD19	F: TTTTATGGCCTTCATTTCAGGGAGC<br>R: TTTTATGGCCTTCATTTCAGGGAGC	
q-PbCAD20	F: CAAGAGGGATGTGAAGTACCGTTTT<br>R: TCAAAGAGAAATATCGATTCCTCAT	
q-PbCAD25	F: GAAGATGTAATGTTGAAGGTTTTGT<br>R: GAACTGCTTCAATGGACTGTAGGCT	
q-PbCAD26	F: GAATGATGTAAGATACCGATTTGTC<br>R: TCCCCTTTGATTTGTCCAACTCCGA	
Tubulin	F: AGAACAAGAACTCGTCCTAC<br>R: GAACTGCTCGCTCACTCTCC	
q-PbCCR1	F: AGAGCAGAATCATACAAGTTCACAA<br>R: TTTTATAAGCAAGATCTAATTTTAA	
q-PbCCR2	F: CGTCCGGAAACAAAGCTAATAAACA<br>R: GGTTCCTCTAACAGTGTAGCCTCTTTCG	
q-PbCCR3	F: CTAATAACCAAGATGCCTGCCGATA<br>R: GGTTCCTCTAACAGTGTAGCCTCTTTCG	
q-PbCCR-like7	F: AAGAACGGAGCGAAGAAG<br>R: AACAGCATCCTTGATAATTTGAT	
q- PbCCR-like9	F: CAAATCAGCAACAGGTAG<br>R: ACTCAGGGTAAAGTTCAG	
q-PbCCR-like11	F: TAGGCACTTGTGTTTGGA	

Biology Open • Supplementary information

	R: GAACCGTGTACTCAGGATAA	
q-PbCCR-like12	F: CCAGTTGAAGATGCTGTG	
	R: TTTGAGTTTGATGGTGATATTTC	
***PbCAD2*-ZH**	F: GAAGATCTGATGGGCGGCCTTGAAGTGGAGAG	
	R: GGACTAGTAATAAGATTGCTGCCGGCCACA	
***PbCAD3*-ZH**	F: GAAGATCTGATGGGCAACCCGGAAGTGGAGAGAA	
	R: GGACTAGTAAGATTGCTACCGGCCACATCG	
***PbCCR1*-ZH**	F: GAAGATCTATGCCTGCCGTTAGCTCGTCAGTTT	**Vector construction**
	R: GGACTAGTAGATTGAATTTTAATGGAATCT	
***PbCCR2*-ZH**	F: CATGCCATGGAAATGCCTGCCGATAGCTCATCAGTCT	
	R: GGACTAGTTAAGCAAGATCTAATTTTAATGGAA	
***PbCCR3*-ZH**	F: GAAGATCTATGCCTGCCGATAGCTCATCAGTTT	
	R: GGACTAGTAGATTGAATTTTAATGGAATCTTGT	

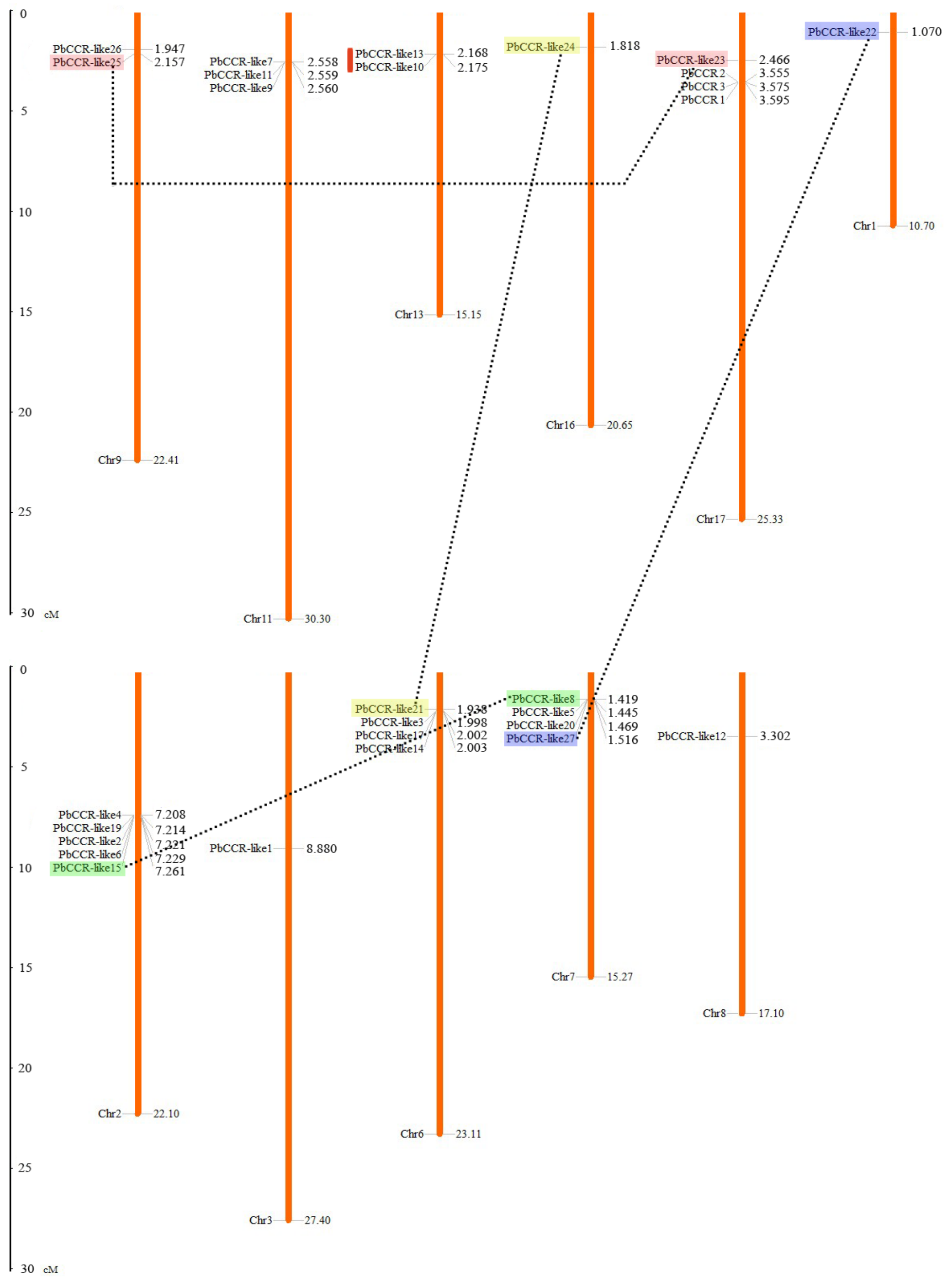

Fig. S1. Chromosomal locations and gene duplications of *PbCCR*s on eleven chromosomes. Genes involved in segmental duplication are joined by dashed lines, and the red lines indicate tandem duplications. The number corresponding to each gene name represents its position along the chromosome, and the number corresponding to each chromosome name represents the total length of the chromosome.

Biology Open • Supplementary information

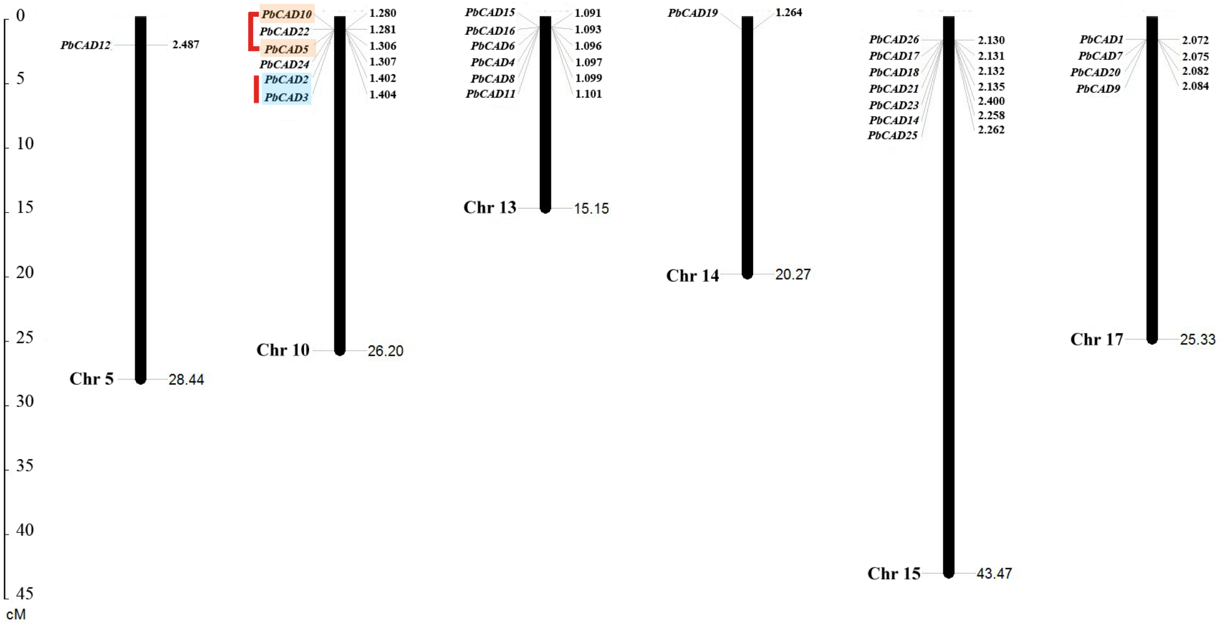

Fig. S2. Chromosomal locations and gene duplications of *PbCAD*s on six chromosomes. Genes involved in tandem duplication are joined by red lines. The number corresponding to each gene name represents its position along the chromosome, and the number corresponding to each chromosome name represents the total length of the chromosome.

Biology Open • Supplementary information

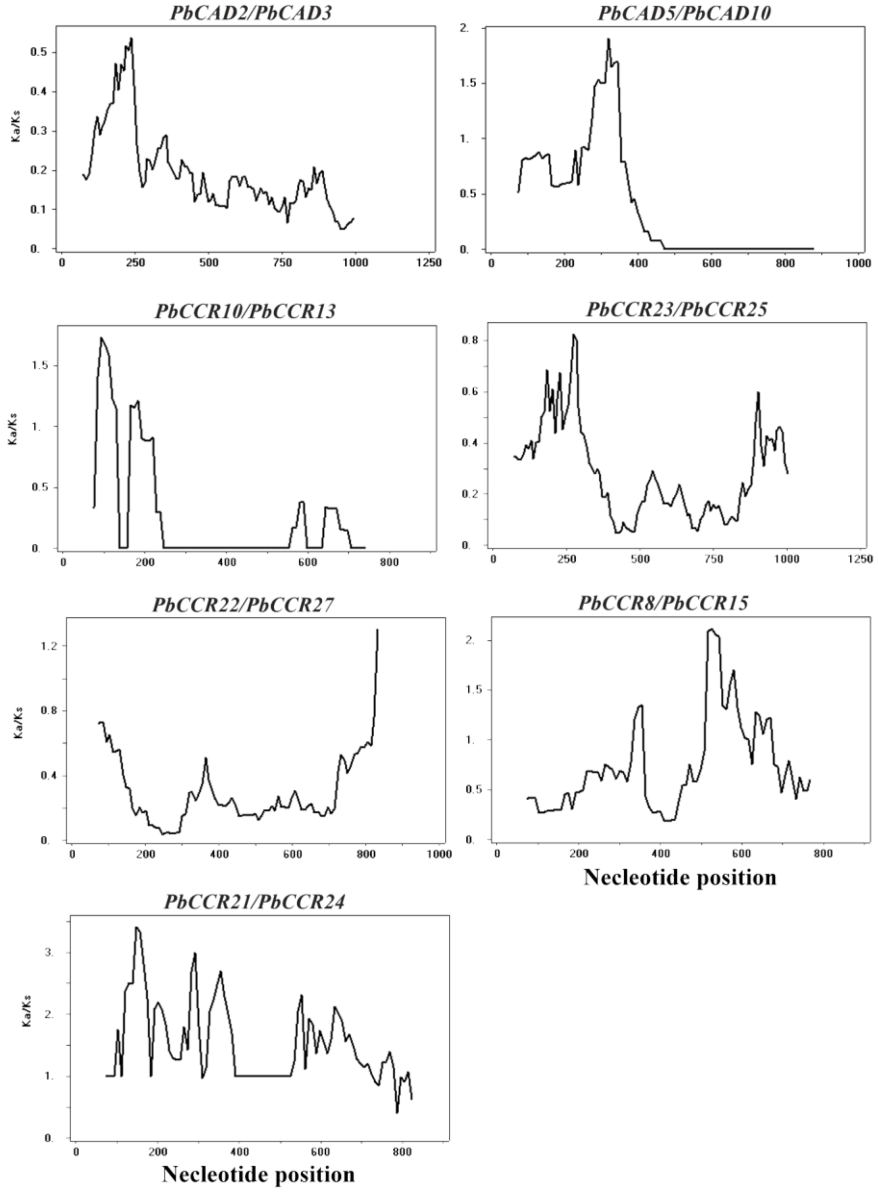

Fig. S3. Sliding window plots of duplicated CCR/CAD genes. The window size was 150 bp, and the step size was 9 bp. The X-axis represents the nucleotide position of the gene pair, and the Y-axis represents the ratio of Ka/Ks.

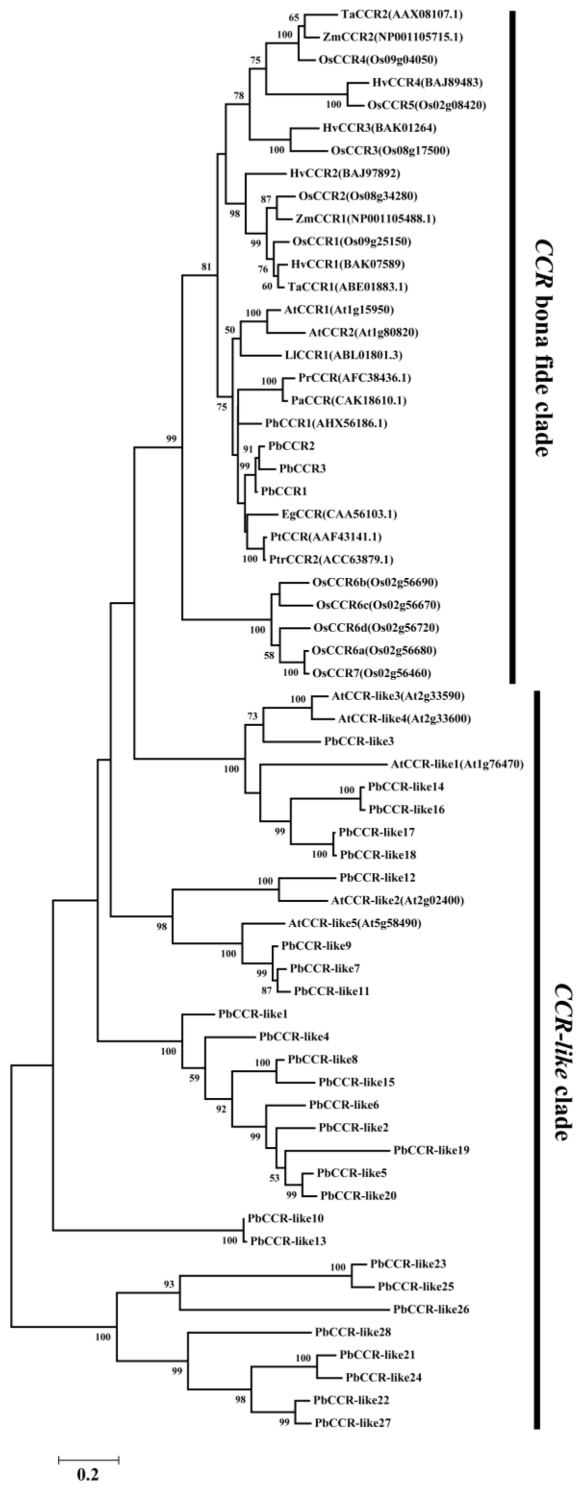

Fig. S4. CCR/CCR-like phylogenetic ML tree based on the full protein sequences of various plants.

Biology Open • Supplementary information

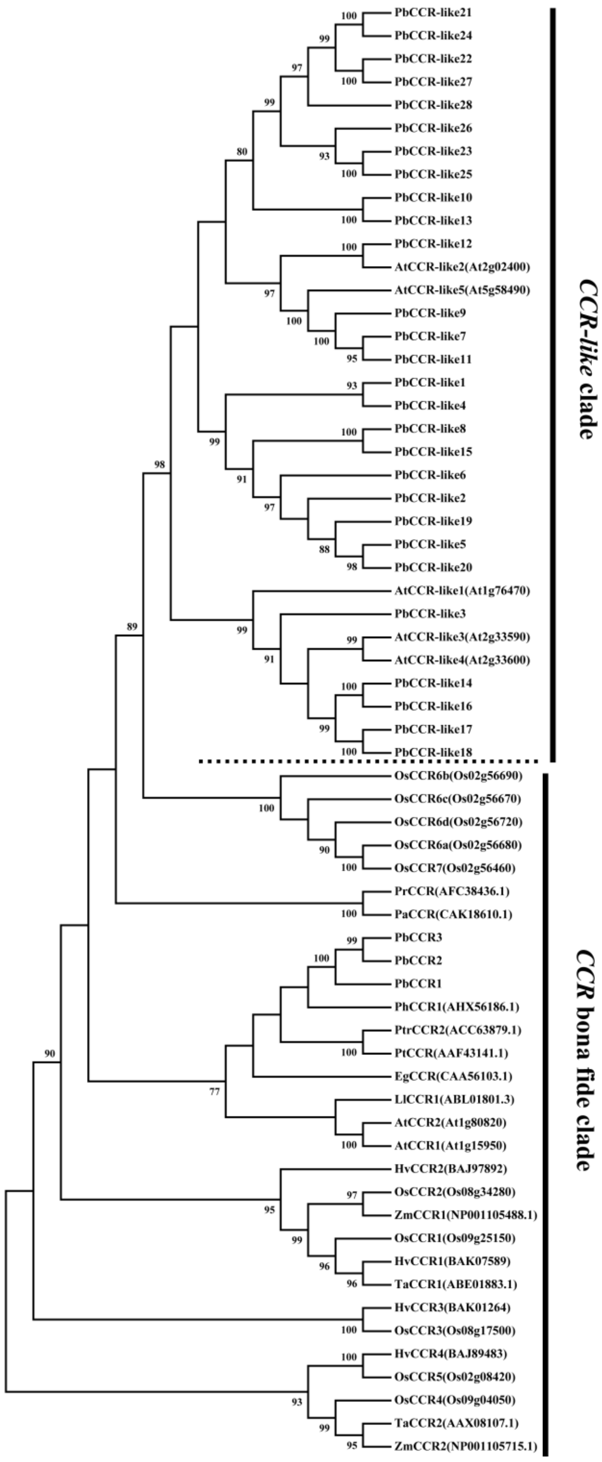

Fig. S5. CCR/CCR-like phylogenetic MP tree based on the full protein sequences of various plants.

Biology Open • Supplementary information

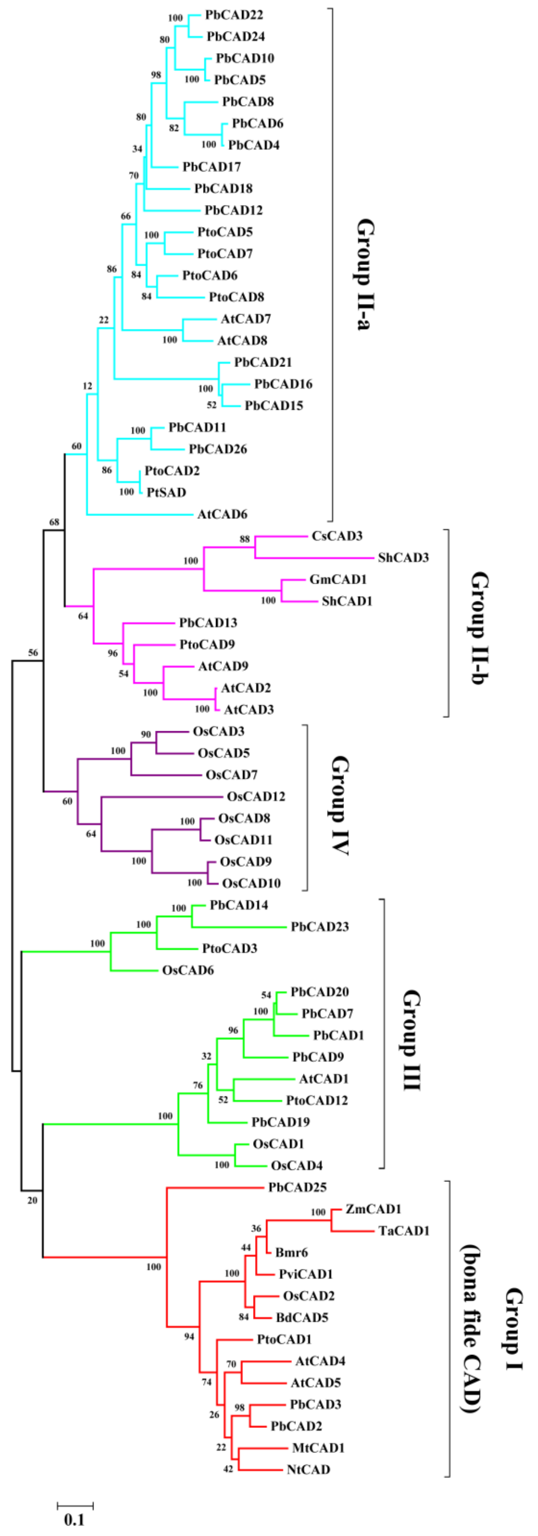

Fig. S6. CADs phylogenetic ML tree based on the full protein sequences of various plants.

Biology Open • Supplementary information

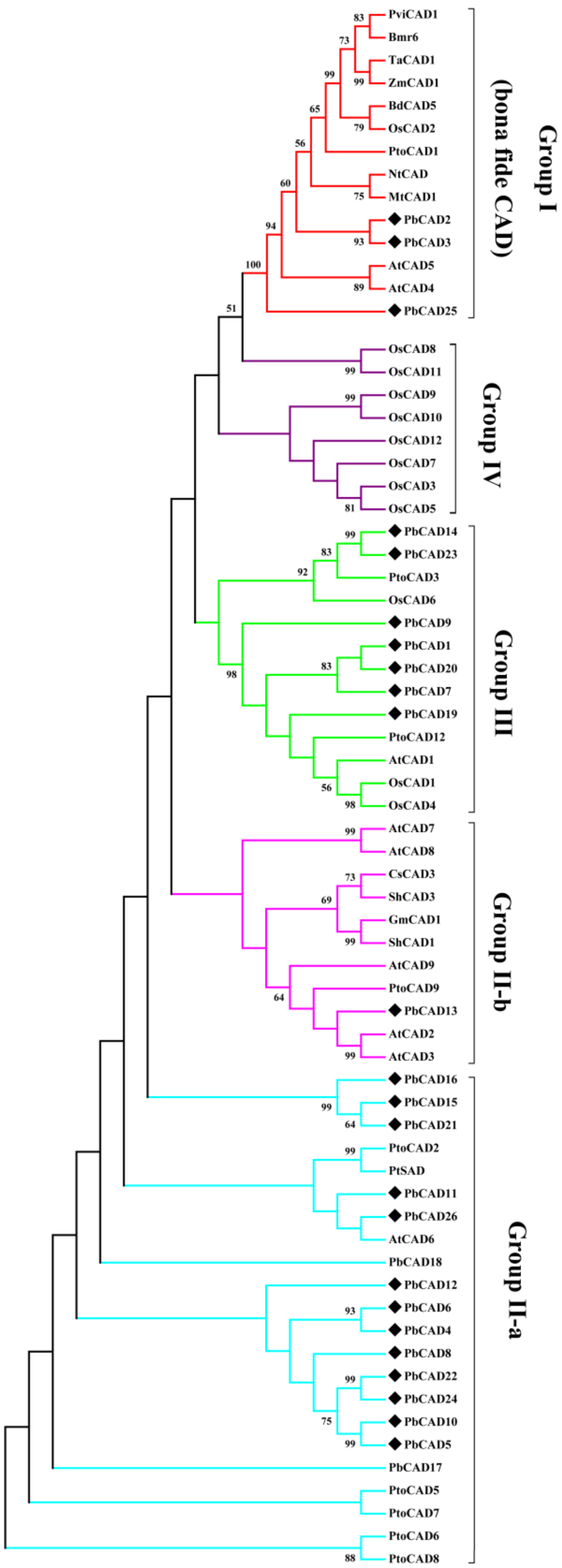

Fig. S7. CADs phylogenetic MP tree based on the full protein sequences of various plants.

Biology Open • Supplementary information

Iden/Sim (%/%)	At CCR1	At CCR2
PbCCR1	75/88	71/87
PbCCR2	74/87	70/86
PbCCR3	72/88	71/88
PbCCR-like1	43/66	42/65
PbCCR-like2	42/68	42/68
PbCCR-like3	41/65	42/66
PbCCR-like4	41/63	38/62
PbCCR-like5	41/56	41/55
PbCCR-like6	13/18	13/18
PbCCR-like7	39/55	38/54
PbCCR-like8	39/53	39/54
PbCCR-like9	35/56	34/55
PbCCR-like10	35/61	35/61
PbCCR-like11	35/58	35/57
PbCCR-like12	39/56	36/54
PbCCR-like13	32/48	31/48
PbCCR-like14	37/56	34/56
PbCCR-like15	35/49	35/50
PbCCR-like16	37/52	37/51
PbCCR-like17	33/47	34/47
PbCCR-like18	31/45	32/46
PbCCR-like19	19/33	19/31
PbCCR-like20	21/31	21/31
PbCCR-like21	20/37	21/38
PbCCR-like22	22/39	21/38
PbCCR-like23	22/38	22/38
PbCCR-like24	20/36	20/38
PbCCR-like25	21/36	20/36
PbCCR-like26	22/37	22/37
PbCCR-like27	21/47	21/48
PbCCR-like28	17/54	17/56

Fig. S8. Sequence identity and similarity among *Arabidopsis* and pear CCR protein homologs.

Iden/Sim (%/%)	At CAD1	At CAD2	At CAD3	At CAD4	At CAD5	At CAD6	At CAD7	At CAD8	At CAD9
PbCAD1	38/56	25/52	25/52	25/54	25/55	26/55	25/56	25/52	26/53
PbCAD2	41/62	47/65	47/65	75/85	80/88	48/65	49/69	49/65	51/69
PbCAD3	43/67	45/67	46/67	73/84	77/87	47/69	50/72	49/70	50/73
PbCAD4	30/60	35/59	35/59	30/60	30/61	41/61	45/62	46/61	40/61
PbCAD5	37/69	45/69	45/69	42/68	40/68	52/74	58/74	58/71	50/73
PbCAD6	38/61	44/61	44/60	40/60	38/59	51/67	55/69	56/69	50/66
PbCAD7	53/61	38/60	37/59	35/57	35/61	37/58	36/58	36/57	36/59
PbCAD8	27/60	32/57	31/57	28/58	27/60	35/59	42/60	42/59	36/59
PbCAD9	69/83	48/68	48/68	44/67	46/70	49/69	51/72	49/70	50/73
PbCAD10	37/61	45/64	44/63	42/62	40/61	51/68	57/69	58/67	50/67
PbCAD11	46/56	56/64	56/64	52/61	52/61	67/75	69/75	68/74	65/75
PbCAD12	46/71	57/74	56/73	50/72	48/71	62/77	71/86	69/80	63/81
PbCAD13	48/66	67/75	67/75	48/64	50/64	58/72	61/72	62/72	76/83
PbCAD14	45/62	52/67	51/67	43/62	41/58	49/63	53/63	54/64	56/70
PbCAD15	47/64	55/67	54/67	46/65	48/65	59/71	62/72	60/70	60/74
PbCAD16	47/64	52/66	52/66	47/65	48/63	57/70	59/72	60/70	57/72
PbCAD17	48/65	58/70	58/70	50/66	49/65	66/76	73/83	72/79	64/77
PbCAD18	48/64	58/69	58/69	48/65	48/64	63/74	72/83	73/81	65/76
PbCAD19	75/86	46/62	46/62	44/61	45/61	49/63	48/65	47/62	48/66
PbCAD20	71/82	46/62	46/62	46/62	46/63	48/62	47/63	47/63	48/65
PbCAD21	42/65	48/68	47/68	40/66	42/66	50/70	55/72	54/72	52/73
PbCAD22	38/65	47/68	47/68	42/68	41/68	51/71	61/77	61/77	51/73
PbCAD23	21/49	24/52	24/53	20/51	19/50	22/50	26/50	25/51	24/50
PbCAD24	30/73	35/70	35/70	31/73	30/73	37/73	47/74	47/74	38/73
PbCAD25	26/70	33/67	33/67	44/69	46/71	35/70	35/71	36/70	34/70
PbCAD26	28/59	32/58	32/58	29/60	30/60	40/60	41/61	41/61	38/60

Fig. S9. Sequence identity and similarity among *Arabidopsis* and pear CAD protein homologs.

Biology Open • Supplementary information

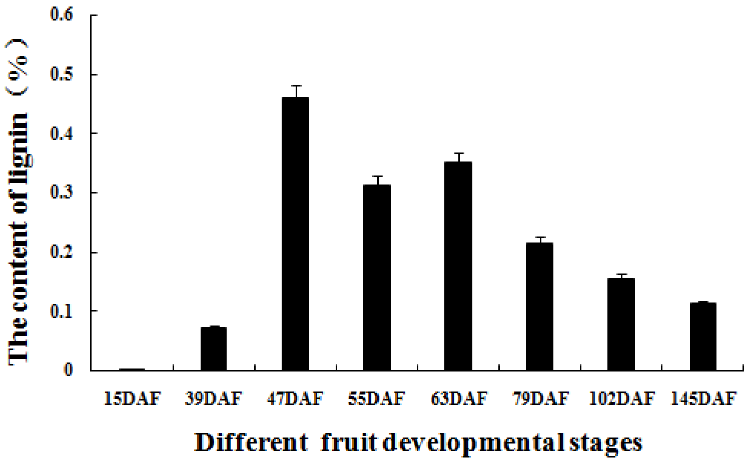

Fig. S10. Lignin content in flesh during *Pyrus bretschneideri* cv. Dangshan Su fruit development. Error bars represent three replicates.

Biology Open • Supplementary information
